# Implementation of corticosteroids in treatment of COVID-19 in the ISARIC WHO Clinical Characterisation Protocol UK: prospective, cohort study

**DOI:** 10.1016/S2589-7500(22)00018-8

**Published:** 2022-03-22

**Authors:** Fiina Närhi, S Ramani Moonesinghe, Susan D Shenkin, Thomas M Drake, Rachel H Mulholland, Cara Donegan, Jake Dunning, Cameron J Fairfield, Michelle Girvan, Hayley E Hardwick, Antonia Ho, Gary Leeming, Jonathan S Nguyen-Van-Tam, Riinu Pius, Clark D Russell, Catherine A Shaw, Rebecca G Spencer, Lance Turtle, Peter J M Openshaw, J Kenneth Baillie, Ewen M Harrison, Malcolm G Semple, Annemarie B Docherty, ISARIC4C investigators, J Kenneth Baillie, Malcolm G Semple, Peter JM Openshaw, Gail Carson, Beatrice Alex, Petros Andrikopoulos, Benjamin Bach, Wendy S Barclay, Debby Bogaert, Meera Chand, Kanta Chechi, Graham S Cooke, Ana da Silva Filipe, Thushan de Silva, Annemarie B Docherty, Gon¸alo dos Santos Correia, Marc-Emmanuel Dumas, Jake Dunning, Tom Fletcher, Christopher A Green, William Greenhalf, Julian Griffin, Rishi K Gupta, Ewen M Harrison, Julian A Hiscox, Antonia YW Ho, Peter W Horby, Samreen Ijaz, Say Khoo, Paul Klenerman, Andrew Law, Matthew Lewis, Sonia Liggi, Wei Shen Lim, Lynn Maslen, Alexander J Mentzer, Laura Merson, Alison M Meynert, Shona C Moore, Mahdad Noursadeghi, Michael Olanipekun, Anthonia Osagie, Massimo Palmarini, Carlo Palmieri, William A Paxton, Georgios Pollakis, Nicholas Price, Andrew Rambaut, David L Robertson, Clark D Russell, Vanessa Sancho-Shimizu, Caroline Sands, Janet T Scott, Louise Sigfrid, Tom Solomon, Shiranee Sriskandan, David Stuart, Charlotte Summers, Olivia V Swann, Zoltan Takats, Panteleimon Takis, Richard S Tedder, AA Roger Thompson, Emma C Thomson, Ryan S Thwaites, Lance CW Turtle, Maria Zambon, Hayley Hardwick, Chloe Donohue, Fiona Griffiths, Wilna Oosthuyzen, Cara Donegan, Rebecca G Spencer, Lisa Norman, Riinu Pius, Thomas M Drake, Cameron J Fairfield, Stephen R Knight, Kenneth A Mclean, Derek Murphy, Catherine A Shaw, Jo Dalton, Michelle Girvan, Egle Saviciute, Stephanie Roberts, Janet Harrison, Laura Marsh, Marie Connor, Sophie Halpin, Clare Jackson, Carrol Gamble, Daniel Plotkin, James Lee, Gary Leeming, Andrew Law, Murray Wham, Sara Clohisey, Ross Hendry, James Scott-Brown, Victoria Shaw, Sarah E McDonald, Seán Keating, Katie A. Ahmed, Jane A Armstrong, Milton Ashworth, Innocent G Asiimwe, Siddharth Bakshi, Samantha L Barlow, Laura Booth, Benjamin Brennan, Katie Bullock, Benjamin WA Catterall, Jordan J Clark, Emily A Clarke, Sarah Cole, Louise Cooper, Helen Cox, Christopher Davis, Oslem Dincarslan, Chris Dunn, Philip Dyer, Angela Elliott, Anthony Evans, Lorna Finch, Lewis WS Fisher, Terry Foster, Isabel Garcia-Dorival, Philip Gunning, Catherine Hartley, Rebecca L Jensen, Christopher B Jones, Trevor R Jones, Shadia Khandaker, Katharine King, Robyn T. Kiy, Chrysa Koukorava, Annette Lake, Suzannah Lant, Diane Latawiec, Lara Lavelle-Langham, Daniella Lefteri, Lauren Lett, Lucia A Livoti, Maria Mancini, Sarah McDonald, Laurence McEvoy, John McLauchlan, Soeren Metelmann, Nahida S Miah, Joanna Middleton, Joyce Mitchell, Shona C Moore, Ellen G Murphy, Rebekah Penrice-Randal, Jack Pilgrim, Tessa Prince, Will Reynolds, P. Matthew Ridley, Debby Sales, Victoria E Shaw, Rebecca K Shears, Benjamin Small, Krishanthi S Subramaniam, Agnieska Szemiel, Aislynn Taggart, Jolanta Tanianis-Hughes, Jordan Thomas, Erwan Trochu, Libby van Tonder, Eve Wilcock, J. Eunice Zhang, Lisa Flaherty, Nicole Maziere, Emily Cass, Alejandra Doce Carracedo, Nicola Carlucci, Anthony Holmes, Hannah Massey, Lee Murphy, Sarah McCafferty, Richard Clark, Angie Fawkes, Kirstie Morrice, Alan Maclean, Nicola Wrobel, Lorna Donelly, Audrey Coutts, Katarzyna Hafezi, Louise MacGillivray, Tammy Gilchrist, Kayode Adeniji, Daniel Agranoff, Ken Agwuh, Dhiraj Ail, Erin L. Aldera, Ana Alegria, Sam Allen, Brian Angus, Abdul Ashish, Dougal Atkinson, Shahedal Bari, Gavin Barlow, Stella Barnass, Nicholas Barrett, Christopher Bassford, Sneha Basude, David Baxter, Michael Beadsworth, Jolanta Bernatoniene, John Berridge, Colin Berry, Nicola Best, Pieter Bothma, David Chadwick, Robin Brittain-Long, Naomi Bulteel, Tom Burden, Andrew Burtenshaw, Vikki Caruth, David Chadwick, Duncan Chambler, Nigel Chee, Jenny Child, Srikanth Chukkambotla, Tom Clark, Paul Collini, Catherine Cosgrove, Jason Cupitt, Maria-Teresa Cutino-Moguel, Paul Dark, Chris Dawson, Samir Dervisevic, Phil Donnison, Sam Douthwaite, Andrew Drummond, Ingrid DuRand, Ahilanadan Dushianthan, Tristan Dyer, Cariad Evans, Chi Eziefula, Chrisopher Fegan, Adam Finn, Duncan Fullerton, Sanjeev Garg, Sanjeev Garg, Atul Garg, Effrossyni Gkrania-Klotsas, Jo Godden, Arthur Goldsmith, Clive Graham, Elaine Hardy, Stuart Hartshorn, Daniel Harvey, Peter Havalda, Daniel B Hawcutt, Maria Hobrok, Luke Hodgson, Anil Hormis, Michael Jacobs, Susan Jain, Paul Jennings, Agilan Kaliappan, Vidya Kasipandian, Stephen Kegg, Michael Kelsey, Jason Kendall, Caroline Kerrison, Ian Kerslake, Oliver Koch, Gouri Koduri, George Koshy, Shondipon Laha, Steven Laird, Susan Larkin, Tamas Leiner, Patrick Lillie, James Limb, Vanessa Linnett, Jeff Little, Mark Lyttle, Michael MacMahon, Emily MacNaughton, Ravish Mankregod, Huw Masson, Elijah Matovu, Katherine McCullough, Ruth McEwen, Manjula Meda, Gary Mills, Jane Minton, Mariyam Mirfenderesky, Kavya Mohandas, Quen Mok, James Moon, Elinoor Moore, Patrick Morgan, Craig Morris, Katherine Mortimore, Samuel Moses, Mbiye Mpenge, Rohinton Mulla, Michael Murphy, Megan Nagel, Thapas Nagarajan, Mark Nelson, Lillian Norris, Matthew K. O'Shea, Igor Otahal, Marlies Ostermann, Mark Pais, Carlo Palmieri, Selva Panchatsharam, Danai Papakonstantinou, Hassan Paraiso, Brij Patel, Natalie Pattison, Justin Pepperell, Mark Peters, Mandeep Phull, Stefania Pintus, Jagtur Singh Pooni, Tim Planche, Frank Post, David Price, Rachel Prout, Nikolas Rae, Henrik Reschreiter, Tim Reynolds, Neil Richardson, Mark Roberts, Devender Roberts, Alistair Rose, Guy Rousseau, Bobby Ruge, Brendan Ryan, Taranprit Saluja, Matthias Schmid, Aarti Shah, Prad Shanmuga, Anil Sharma, Anna Shawcross, Jeremy Sizer, Manu Shankar-Hari, Richard Smith, Catherine Snelson, Nick Spittle, Nikki Staines, Tom Stambach, Richard Stewart, Pradeep Subudhi, Tamas Szakmany, Kate Tatham, Jo Thomas, Chris Thompson, Robert Thompson, Ascanio Tridente, Darell Tupper-Carey, Mary Twagira, Nick Vallotton, Rama Vancheeswaran, Lisa Vincent-Smith, Shico Visuvanathan, Alan Vuylsteke, Sam Waddy, Rachel Wake, Andrew Walden, Ingeborg Welters, Tony Whitehouse, Paul Whittaker, Ashley Whittington, Padmasayee Papineni, Meme Wijesinghe, Martin Williams, Lawrence Wilson, Sarah Sarah, Stephen Winchester, Martin Wiselka, Adam Wolverson, Daniel G Wootton, Andrew Workman, Bryan Yates, Peter Young

**Affiliations:** a College of Medicine and Veterinary Medicine, University of Edinburgh, Edinburgh, UK; b Department for Targeted Intervention, Division of Surgery and Interventional Science, University College London, London, UK; c Health Services Research Centre, National Institute for Academic Anaesthesia, Royal College of Anaesthetists, London, UK; d Geriatric Medicine, University of Edinburgh, Edinburgh, UK; e Centre for Medical Informatics, University of Edinburgh, Edinburgh, UK; f The Breathe Hub, University of Edinburgh, Edinburgh, UK; g Usher Institute, Centre for Inflammation Research, Queen's Medical Research Institute, University of Edinburgh, Edinburgh, UK; h The Roslin Institute, University of Edinburgh, Edinburgh, UK; i Department of Biostatistics, University of Liverpool, Liverpool, UK; j NIHR Health Protection Research Unit in Emerging and Zoonotic Infections, Institute of Infection and Global Health, University of Liverpool, Liverpool, UK; k Faculty of Medicine, Imperial College London, London, UK; l MRC-University of Glasgow Centre for Virus Research, University of Glasgow, Glasgow, UK; m Division of Epidemiology and Public Health, University of Nottingham, Nottingham, UK; n UK Department of Health and Social Care, Field Epidemiology Service, London, UK

## Abstract

**Background:**

Dexamethasone was the first intervention proven to reduce mortality in patients with COVID-19 being treated in hospital. We aimed to evaluate the adoption of corticosteroids in the treatment of COVID-19 in the UK after the RECOVERY trial publication on June 16, 2020, and to identify discrepancies in care.

**Methods:**

We did an audit of clinical implementation of corticosteroids in a prospective, observational, cohort study in 237 UK acute care hospitals between March 16, 2020, and April 14, 2021, restricted to patients aged 18 years or older with proven or high likelihood of COVID-19, who received supplementary oxygen. The primary outcome was administration of dexamethasone, prednisolone, hydrocortisone, or methylprednisolone. This study is registered with ISRCTN, ISRCTN66726260.

**Findings:**

Between June 17, 2020, and April 14, 2021, 47 795 (75·2%) of 63 525 of patients on supplementary oxygen received corticosteroids, higher among patients requiring critical care than in those who received ward care (11 185 [86·6%] of 12 909 *vs* 36 415 [72·4%] of 50 278). Patients 50 years or older were significantly less likely to receive corticosteroids than those younger than 50 years (adjusted odds ratio 0·79 [95% CI 0·70–0·89], p=0·0001, for 70–79 years; 0·52 [0·46–0·58], p<0·0001, for >80 years), independent of patient demographics and illness severity. 84 (54·2%) of 155 pregnant women received corticosteroids. Rates of corticosteroid administration increased from 27·5% in the week before June 16, 2020, to 75–80% in January, 2021.

**Interpretation:**

Implementation of corticosteroids into clinical practice in the UK for patients with COVID-19 has been successful, but not universal. Patients older than 70 years, independent of illness severity, chronic neurological disease, and dementia, were less likely to receive corticosteroids than those who were younger, as were pregnant women. This could reflect appropriate clinical decision making, but the possibility of inequitable access to life-saving care should be considered.

**Funding:**

UK National Institute for Health Research and UK Medical Research Council.

## Introduction

During the COVID-19 pandemic, several large-scale trials have attempted to identify life-saving therapies. Currently, the most robust recommendations are for corticosteroids.^1^

Dexamethasone is a readily available, commonly used, and cheap intervention^2^ that decreased all-cause 28-day mortality in adult patients (aged ≥18 years) admitted to hospital with COVID-19 by an absolute risk reduction of 2·8% in the open-label Randomized Evaluation of COVID-19 Therapy (RECOVERY) trial.^3^ Greater benefit was found in patients on higher levels of respiratory support and, importantly, there was possibility of harm in those not receiving oxygen therapy.^3^ On June 16, 2020, when preliminary results from RECOVERY were published,^4^ UK clinical guidelines were updated to recommend low-dose oral or intravenous corticosteroids—dexamethasone, prednisolone, methylprednisolone, or hydrocortisone—to patients with COVID-19 requiring supplementary oxygen.^5^

On Sept 2, 2020, the Rapid Evidence Appraisal for COVID-19 (REACT) meta-analysis of critically ill patients with COVID-19 showed lower mortality associated with administration of corticosteroids than with standard care (summary odds ratio 0·66).^6^ The next day, the UK National Institute for Health and Care Excellence (NICE) and WHO published guidelines recommending oral or intravenous administration of 6 mg dexamethasone for up to 10 days to patients with severe or critical COVID-19, defined by oxygen saturation (SpO_2_) of less than 90%, a respiratory rate of more than 30 breaths per min, sepsis, septic shock, acute respiratory distress syndrome, mechanical ventilation, vasopressor therapy, or signs of severe respiratory distress.^7^ Months later, on April 8, 2021, NICE updated their guideline to recommend corticosteroids for all patients requiring supplementary oxygen, noting “the need for clear and unambiguous terminology”.^8^

**Research in context**

**Evidence before this study**

We searched PubMed for primary research articles documenting changes in rates of corticosteroid administration in COVID-19 over time, published between June 16, 2020, and July 8, 2021, with no language restrictions and using the search terms (“SARS-CoV-2” OR “COVID-19”) AND (“corticosteroids” OR “steroids” OR “dexamethasone”). Of the 1985 studies identified, most focused on the relationship between corticosteroids and outcome, led by the RECOVERY trial. We found no studies that documented changes in corticosteroid administration over time or those that identified differential administration between patient groups. Understanding the implementation of corticosteroids and identifying groups of patients who are systematically less likely to be prescribed steroids are essential to ensure that this cheap and effective medication is available to all eligible patients.

**Added value of this study**

Although randomised controlled trials have convincingly shown the mortality benefit of corticosteroids for patients on supplemental oxygen, no studies have previously looked at whether this evidence has translated into clinical practice. We have shown that the majority of patients on oxygen in UK hospitals have been prescribed corticosteroids. However, we found that corticosteroid administration was significantly lower for patients older than 70 years, and this low uptake could not be explained by other factors associated with lower rates of steroid administration, such as dementia, clinical frailty, and treatment escalation decisions.

**Implications of all the available evidence**

The quick implementation of one of the only mortality-reducing therapies for COVID-19 has been very successful in the UK. However, the potential inequities in administration that we have found are extremely important for individual, local, and global health-care communities to evaluate their practices in treating COVID-19 and to ensure all eligible patients receive the best available treatment.

A modelling study estimated that corticosteroids could save approximately 12 000 lives between July and December, 2020, in the UK.^9^ However, for this treatment to save lives it has to be successfully implemented. As implementation of a new intervention relies on behavioural change, it benefits from an understanding of baseline behaviours and detailed and explicit recommendations that consider factors relevant to decision making.^10^ Common challenges include complexity of the intervention and difficulty gaining consensus with colleagues.^10^ Success in implementing a new intervention is usually defined as an uptake of 80–90%.^11^

As of May, 2021, only an intermediate report from October, 2020, from the International Severe Acute Respiratory and Emerging Infections Clinical Characterisation Protocol UK (ISARIC4C CCP-UK) investigators had explored corticosteroid uptake in the UK, estimating that 55% of adult patients on oxygen in hospital had received corticosteroids.^12^ It is unknown how the changing guidelines and potential barriers have affected the transition from evidence to practice. We aimed to evaluate the implementation of corticosteroids into UK clinical practice since the announcement of the RECOVERY trial findings.

## Methods

### Study design and participants

We did an audit of clinical implementation using data from the ISARIC WHO Clinical Characterisation Protocol UK (CCP-UK), a prospective, observational cohort study representing nearly half of UK COVID-19 hospital admissions.^13^ We included patients aged 18 years or older who were admitted to 237 acute general hospitals with PCR-confirmed COVID-19 (or with high likelihood of disease if supporting PCR results were not available) from assumed community-acquired infection between March 16, 2020, and April 14, 2021. Community-acquired infection was defined as symptom onset 5 days or less after admission to hospital. Patients who received any supplementary oxygen at any point during their hospital stay were included in analyses.

Patient data, including patient demographics, comorbidities, treatments, complications, and outcomes, were recorded into the secure REDCap database. The detailed study protocol and ethics approval are described elsewhere.^13^ Under the Control of Patient Information notice 2020 for urgent public health research, processing of demographic and routine clinical data from medical records for research does not require consent in England and Wales.^14^ In Scotland, a waiver for consent was obtained from the Public Benefit and Privacy Panel.^15^ Reporting of this study conforms to the STROBE statement.^16^

### Variables

We collected information using the ISARIC4C case report form for key variables, including age (categorised into <50 years, 50–59 years, 60–69 years, 70–79 years, and ≥80 years, corresponding to the ISARIC4C Mortality Score^17^ categories), sex, self-reported ethnicity, deprivation (according to the Index of Multiple Deprivation, with the first quintile being the least deprived and the fifth quintile the most deprived), Clinical Frailty Score (scores of 1–2 indicate fit, 3–4 indicate vulnerable, but not frail, 5–6 indicate initial signs of frailty but with some degree of independence, and 7–9 indicate severe or very severe frailty),^18^ and comorbidities (presence of any one or more of hypertension, chronic cardiac disease non-asthmatic chronic pulmonary disease, asthma, type 1 diabetes, type 2 diabetes, obesity, chronic neurological disease, dementia, chronic kidney disease, moderate or severe liver disease, mild liver disease, malignancy, rheumatological disease, pre-admission immunosuppressants including corticosteroids, and HIV/AIDS). Physiological parameters of the ISARIC4C Mortality Score (SpO_2_, respiratory rate, C-reactive protein [CRP], blood urea nitrogen, Glasgow Coma Scale score)^17^ within 24 h of hospital admission were used as markers of illness severity. Admitting hospitals were mapped to the NHS regions to create the NHS region variable. These are reported for the patients with moderate or severe COVID-19 to account for the differences in patient cohort severity and thus eligibility for corticosteroid therapy. The ISARIC4C case report form collected information on whether or not patients had an admission to an intensive care or high dependency unit (these were not separated). We further categorised patients in critical care by receipt of invasive ventilation. The any oxygen variable represents patients who were recorded to receive any supplementary oxygen or any invasive ventilation (this was assumed to involve supplementary oxygen), or to have daily fraction FiO_2_ of more than 0·21 on any day of hospital admission. Corticosteroid administration, maximum level of respiratory support (no oxygen, supplemental oxygen, non-invasive ventilation, or invasive mechanical ventilation), and critical care admission were recorded at any timepoint during admission. We have provided further detail on variables in the appendix (pp 1–2).

Patients were recorded to have symptoms at admission if they reported any one or more of the following: cough, fever, sore throat, runny nose, ear pain, wheezing, chest pain, myalgia, joint pain, fatigue, shortness of breath, disturbance or loss of taste, lower chest wall indrawing, headache, altered consciousness or confusion, seizures, abdominal pain, vomiting or nausea, diarrhoea, conjunctivitis, skin rash, skin ulcers, lymphadenopathy, bleeding, or anosmia. If none of these were reported, patients were considered to be asymptomatic.

Corticosteroid treatment was recorded as a free text entry, which we interpreted using an algorithm described in the appendix (p 1) and checked manually. Dexamethasone and hydrocortisone, and prednisolone in pregnant women, are recommended by NICE,^8^ but methylprednisolone is also considered an appropriate alternative to these drugs.^1^ We created a variable, any corticosteroid, signifying administration of any of these corticosteroids during the admission; patients were therefore stratified by those who received once-daily 6 mg dexamethasone orally or intravenously, those who received any dose or frequency of dexamethasone, those who received any corticosteroid, and those who received none.

### COVID-19 severity

Since the initial guidance considered all patients requiring supplementary oxygen to be eligible for corticosteroid treatment,^5^ we primarily investigated corticosteroid administration in patients recorded to have received supplementary oxygen. To account for the severity criteria added in September, 2020, we further categorised these patients into those with mild COVID-19, moderate COVID-19, or severe COVID-19 by clinical severity on hospital admission. Severe COVID-19 reflects the WHO criteria:^2^ SpO_2_ of less than 90% or a respiratory rate of more than 30 breaths per min. Moderate COVID-19 includes patients with clinical respiratory distress (respiratory rate >20 breaths per min) and SpO_2_ threshold for supplementary oxygen (<94%).^19^ Patients not fulfilling these criteria might have transiently received oxygen at admission with minimal respiratory distress, whom we categorised as having mild COVID-19. The appendix (pp 16–20) shows the severity stratification of patients by various severity markers, demographics, and comorbidities.

### Outcomes

We defined two time-based cohorts: admissions between March 16 and June 16, 2020 (baseline cohort), and between June 17, 2020, and April 14, 2021 (after the RECOVERY press release^4^ and allowing 28-day follow-up; second cohort).

The primary outcome was the proportion of patients in the second cohort who received any oral or intravenous corticosteroid (dexamethasone, prednisolone, hydrocortisone, or methylprednisolone). Patients who received supplementary oxygen were considered eligible, but we also explored the effect of the COVID-19 severity criteria. The secondary outcome was the change in administration of corticosteroids before (ie, in the baseline cohort) and after the publication of the RECOVERY trial results (ie, the second cohort), in relation to the RECOVERY trial and NICE guidance. The second cohort was further characterised and studied for factors associated with corticosteroid administration.

### Statistical analysis

Continuous data are summarised as median (IQR) and categorical data as frequency (percentage). We used statistical disclosure control measures to protect patient confidentiality and anonymity. Cells with fewer than five patients were either removed or replaced with not applicable or merged with other levels manually (not applicable could be 0 or suppressed).

We modelled corticosteroid use with a multiple logistic regression model, adjusting for demographic variables and variables previously shown to be associated with disease severity: age, sex, ethnicity, deprivation quintile, and comorbidities (hypertension, chronic cardiac disease, non-asthmatic chronic pulmonary disease, asthma, diabetes, chronic neurological disease, dementia, chronic kidney disease, moderate or severe liver disease, mild liver disease, malignancy, rheumatological disease, pre-admission immunosuppressants, and HIV/AIDS), with the aim to explain corticosteroid implementation. We also adjusted for time (week of admission) and the COVID-19 severity criteria. The model was built using a parsimonious criterion-based approach.^20^ This approach incorporated geographical clustering of patients through the inclusion of a random effect at the level of the admitting hospital, checking for all first-order interactions, including the effect of time (week of admission as linear and quadratic terms), and final model selection by the Akaike information criterion. We report *C* statistics as measures of model discrimination. We set statistical significance at 5%. We checked that the data were coded correctly, which identified the potential erroneous coding of all patients in some hospitals with an intensive care unit admission. We excluded these patients from the analysis. We identified missing values within each variable and analysed the patterns of missingness. We visually checked for associations between missing and observed data. For patients whose postcodes were missing, we used the average Index of Multiple Deprivation rank, weighted by population in each lower super output area for a given hospital catchment area. We considered comorbidity indicated as unknown as no comorbidity. We considered corticosteroid administration reported missing as no corticosteroid and did a sensitivity analysis of patients with a yes versus no entry.

We acknowledge the missing data in all our tables but did not perform any imputation for missing data, as given the nature of the study, the analysis team had concerns around interpretation if the corticosteroid administration were to be imputed.

We checked data for patterns of missingness, indicate missing data in the study tables, and describe missing data in the appendix (pp 6–29). The denominator for explanatory variables, such as comorbidities, is the number of patients with completed entries. The proportion of patients receiving any corticosteroid is reported as a percentage of the total number of participants, with the denominator including participants with missing corticosteroid entry.

We did sensitivity analyses: complete case analysis excluding patients with missing corticosteroid administration data, patients with recorded PCR-positive COVID-19, and a cohort excluding participants with an outcome of death or palliative discharge recorded within 2 days of admission to account for patients who might have been identified as palliative from the beginning and therefore not started on corticosteroid treatment. Frailty was only collected from Aug 1, 2020, onwards, and we did a sensitivity analysis for patients with frailty recorded.

We did an interrupted time-series analysis using segmented regression, with the timepoint of interest being June 16, 2020, when the RECOVERY trial findings and subsequent UK clinical guidance were published.^4^, ^5^ The primary question was whether a change in absolute values (intercept) existed after the timepoint of interest. We considered time periods before (from March 16, 2020, to June 16, 2020; baseline cohort) and after (from June 17, 2020, and April 14, 2021; second cohort), for which we tested and reported trend lines with estimated 95% CIs. We investigated two subgroups with lower corticosteroid prescribing rates: patients with moderate or severe COVID-19 who were 80 years or older, or pregnant.

We undertook sensitivity analyses to evaluate the validity of these criteria relative to other reported severity markers,^17^ including CRP and fraction of inspired oxygen (FiO_2_). Aiming to avoid analysis of patients not requiring oxygen, and for whom there is a conditional recommendation against the use of corticosteroids,^8^ we modelled the factors associated with corticosteroid administration only in patients with moderate or severe COVID-19.

We analysed data using R (version 3.6.3), with packages including tidyverse, finalfit, and gridExtra.

### Role of the funding source

The funders of this study had no role in the study design, data collection, data analysis, data interpretation, writing of the report, or the decision to submit for publication.

## Results

**Figure 1:**
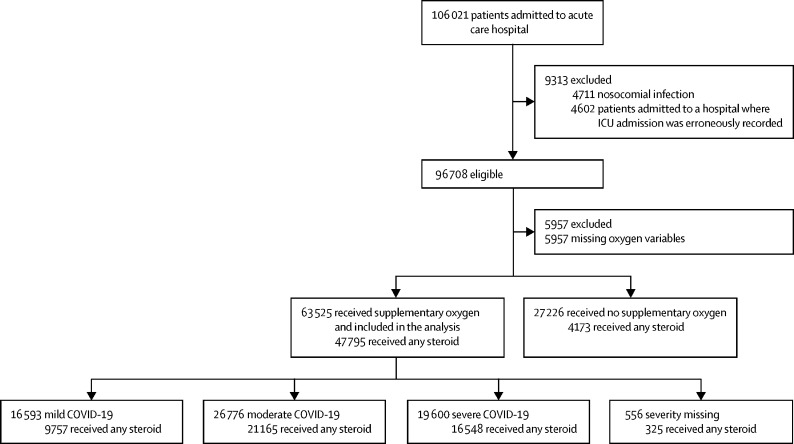
Trial profile ICU=intensive care unit.

ISARIC WHO CCP-UK enrolled 106 021 patients between June 17, 2020, and April 14, 2021 (figure 1). Of 96 708 eligible patients with COVID-19, 63 525 (65·7%) received supplementary oxygen and were included in analyses (figure 1; appendix pp 6–10). Of patients receiving oxygen, 16 593 (26·1%) had mild COVID-19, 26 776 (42·2%) had moderate COVID-19, and 19 600 (30·9%) had severe COVID-19 (figure 1).

**Figure 2:**
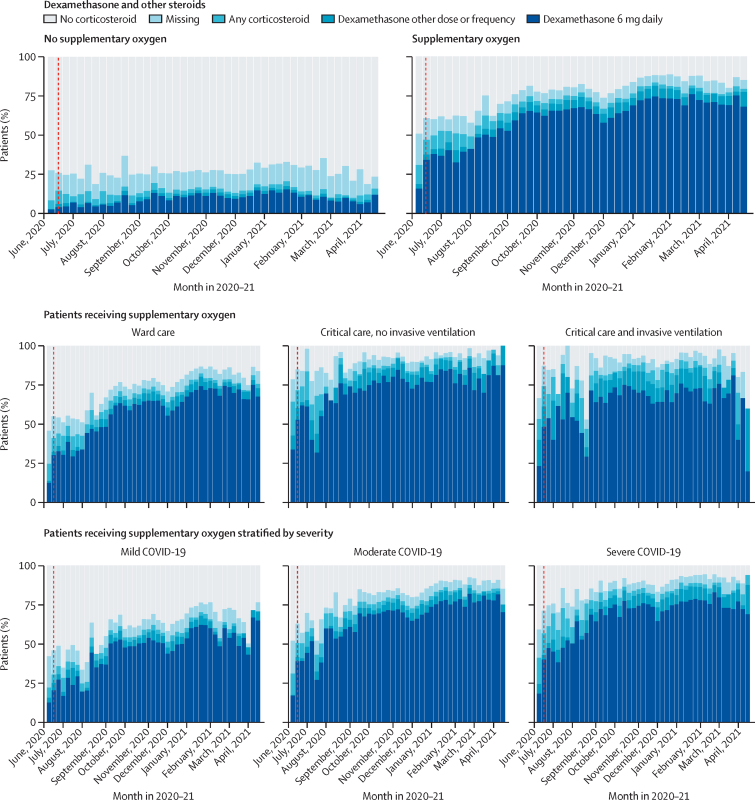
Corticosteroid administration in patients between the June 8, 2020, and April 14, 2021, stratified by supplementary oxygen, level of care, and COVID-19 severity at hospital admission (A) All patients stratified by no or any supplementary oxygen. (B) Patients with any supplementary oxygen stratified by level of care: ward, those with critical care admission but without invasive ventilation and those with critical care admission and invasive ventilation. (C) Patients with any supplementary oxygen stratified by severity of illness. The red dashed line indicates June 16, 2020, when RECOVERY results were published.

**Figure 3:**
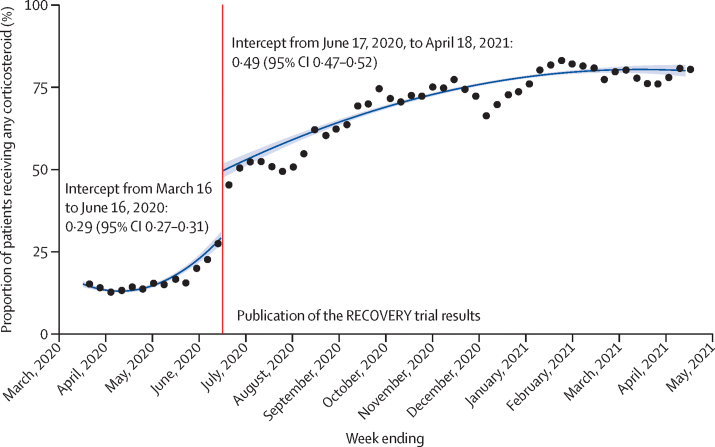
Fitted lines of the linear regression model for corticosteroid administration to patients who required oxygen, admitted to hospital between March 16, 2020, and April 18, 2021 Points represent weekly percentage of patients receiving any corticosteroid (dexamethasone, hydrocortisone, prednisolone, or methylprednisolone). The red vertical line represents the publication of the RECOVERY trial results and initial guidelines for steroid administration on June 16, 2020. Shaded areas around the fitted lines represent 95% CIs.

The proportion of patients on oxygen receiving corticosteroids increased from June, 2020, across levels of care and COVID-19 severity categories, with a clear step up in corticosteroid administration occurring immediately, and a subsequent continuing improvement plateauing in October, 2020 (figure 2). The intercepts of the segmented regression for daily proportion of patients on oxygen receiving any corticosteroid before and after June 16, 2020, showed a significant increase from 0·29 (95% CI 0·27–0·31) to 0·49 (0·47–0·52; p<0·0001; figure 3) This result parallels the observed increase in weekly proportion of patients receiving corticosteroids after publication of the RECOVERY trial results (appendix pp 49–50),^4^ which was similar across NHS regions (appendix p 51).

Of the 63 525 patients on oxygen, 42 442 (66·8%) received 6 mg dexamethasone once daily, 47 795 (75·2%) received any corticosteroid, 13 251 (20·9%) received no corticosteroid, and 2479 (3·9%) had missing corticosteroid data (table 1). Of the 47 795 patients who received any corticosteroid, 42 442 (88·8%) received 6 mg dexamethasone once daily, 4313 (9·0%) received another or unknown dose or frequency of dexamethasone, 558 (1·2%) received hydrocortisone, 456 (1·0%) received prednisolone, and 26 (0·1%) received methylprednisolone. Patients who received any corticosteroid were younger than those who received none (table 1).

**Table 1:** Baseline characteristics of patients admitted between June 17, 2020, and April 14, 2021, who received supplementary oxygen at any point in their admission, stratified by corticosteroid administration

		**6 mg once-daily dexamethasone group (n=42 442)**	Any corticosteroid group*(n=47 795)	**No corticosteroid group (n=13 251)**	**Missing corticosteroid group (n=2479)**
**Patient demographics**					
PCR-positive COVID-19		38 424 (90·5%)	43 287 (90·6%)	11 927 (90·0%)	1265 (51·0%)
Age on admission, years (n=63 525)		68·0 (55·7–79·3)	68·1 (55·7–79·4)	77·8 (64·3–85·9)	70·5 (56·6–81·2)
Age, years					
	<50	6584 (15·5%)	7431 (15·5%)	1418 (10·7%)	358 (14·4%)
	50–59	7729 (18·2%)	8625 (18·0%)	1244 (9·4%)	408 (16·5%)
	60–69	8602 (20·3%)	9567 (20·0%)	1652 (12·5%)	449 (18·1%)
	70–79	9517 (22·4%)	10 786 (22·6%)	3088 (23·3%)	592 (23·9%)
	≥80	10 010 (23·6%)	11 386 (23·8%)	5849 (44·1%)	672 (27·1%)
Sex (n=63 435)					
	Female	17 285/42 380 (40·8%)	19 558/47 723 (41·0%)	6483/13 238 (49·0%)	1039/2474 (42·0%)
	Male	25 095/42 380 (59·2%)	28 165/47 723 (59·0%)	6755/13 238 (51·0%)	1435/2474 (58·0%)
Ethnicity					
	White	29 880/36 897 (81·0%)	33 663/41 515 (81·1%)	10 304/11 659 (88·4%)	1551/1821 (85·2%)
	South Asian	2972/36 897 (8·1%)	3325/41 515 (8·0%)	518/11 659 (4·4%)	61/1821 (3·3%)
	East Asian	229/36 897 (0·6%)	248/41 515 (0·6%)	41/11 659 (0·4%)	11/1821 (0·6%)
	Black	982/36 897 (2·7%)	1111/41 515 (2·7%)	202/11 659 (1·7%)	46/1821 (2·5%)
	Other ethnic minority	2834/36 897 (7·7%)	3168/41 515 (7·6%)	594/11 659 (5·1%)	152/1821 (8·3%)
Pregnancy (only recorded for women aged 12–55 years)					
	No	3498/3582 (97·7%)	3931/4051 (97·0%)	772/937 (82·4%)	165/183 (90·2%)
	Yes	84/3582 (2·3%)	120/4051 (3·0%)	165/937 (17·6%)	18/183 (9·8%)
NHS region					
	East of England	4101/42 164 (9·7%)	4856/47 484 (10·2%)	1775/13 172 (13·5%)	170/2453 (6·9%)
	London	3260/42 164 (7·7%)	3634/47 484 (7·7%)	625/13 172 (4·7%)	120/2453 (4·9%)
	Midlands	8986/42 164 (21·3%)	10 346/47 484 (21·8%)	3104/13 172 (23·6%)	311/2453 (12·7%)
	North East and Yorkshire	6774/42 164 (16·1%)	7703/47 484 (16·2%)	1883/13 172 (14·3%)	186/2453 (7·6%)
	Northern Ireland	58/42 164 (0·1%)	61/47 484 (0·1%)	NA	NA
	North West	8172/42 164 (19·4%)	9042/47 484 (19·0%)	2477/13 172 (18·8%)	366/2453 (14·9%)
	Scotland	946/42 164 (2·2%)	1002/47 484 (2·1%)	196/13 172 (1·5%)	86/2453 (3·5%)
	South East	5336/42 164 (12·7%)	5914/47 484 (12·5%)	1826/13 172 (13·9%)	946/2453 (38·6%)
	South West	3436/42 164 (8·1%)	3679/47 484 (7·7%)	940/13 172 (7·1%)	255/2453 (10·4%)
	Wales	1095/42 164 (2·6%)	1247/47 484 (2·6%)	346/13 172 (2·6%)	13/2453 (0·5%)
**Severity of illness at admission**					
	Oxygen saturation (SpO_2_), % (n=62 726)	92·0% (89·0–95·0)	92·0% (89·0–95·0)	94·0% (92·0–96·0)	93·0% (90·0–95·0)
	Respiratory rate, breaths per min (n=59 739)	22·0 (20·0–28·0)	22·0 (20·0–28·0)	20·0 (18·0–24·0)	22·0 (19·0–26·0)
	C-reactive protein, mg/dL (n=50 470)	93·0 (49·0–156·4)	93·0 (49·0–157·0)	52·2 (18·0–112·0)	87·0 (39·0–150·0)
	Blood urea nitrogen, mg/dL (n=50 591)	6·5 (4·7–9·7)	6·6 (4·7–9·8)	7·3 (5·0–11·5)	6·8 (4·8–10·6)
Glasgow coma scale†					
	15	37 408/40 675 (92·0%)	41 738/45 724 (91·3%)	10 919/12 467 (87·6%)	2006/2274 (88·2%)
	<15	3267/40 675 (8·0%)	3986/45 724 (8·7%)	1548/12 467 (12·4%)	268/2274 (11·8%)
Highest fraction of inspired oxygen (FiO_2_) on day of hospital admission (n=59 026)		0·32 (0·24–0·50)	0·32 (0·24–0·50)	0·21 (0·21–0·28)	0·32 (0·24–0·50)
Severity criteria					
	Mild COVID-19	8575/42 155 (20·3%)	9757/47 470 (20·6%)	6095/13 095 (46·5%)	741/2404 (30·8%)
	Moderate COVID-19	19 092/42 155 (45·3%)	21 165/47 470 (44·6%)	4608/13 095 (35·2%)	1003/2404 (41·7%)
	Severe COVID-19	14 488/42 155 (34·4%)	16 548/47 470 (34·9%)	2392/13 095 (18·3%)	660/2404 (27·5%)
**Comorbidities**					
Any comorbidity					
	No	6766/40 263 (16·8%)	7446/45 376 (16·4%)	1299/12 608 (10·3%)	293/1671 (17·5%)
	Yes	33 497/40 263 (83·2%)	37 930/45 376 (83·6%)	11 309/12 608 (89·7%)	1378/1671 (82·4%)
	Hypertension	18 304/39 059 (46·9%)	20 633/43 959 (46·9%)	6155/12 033 (51·2%)	680/1501 (45·3%)
	Chronic cardiac disease	10 268/39 118 (26·2%)	11 650/44 069 (26·4%)	4578/12 173 (37·6%)	406/1558 (26·1%)
	Non-asthmatic chronic pulmonary disease	6540/39 133 (16·7%)	7581/44 087 (17·2%)	2388/12 154 (19·6%)	261/1549 (16·8%)
Asthma		6374/39 121 (16·3%)	7256/44 078 (16·5%)	1594/12 112 (13·2%)	230/1541 (14·9%)
Type 1 diabetes		797/38 651 (2·1%)	894/43 555 (2·1%)	314/11 964 (2·6%)	53/1520 (3·5%)
Type 2 diabetes		10 737/38 651 (27·8%)	12 035/43 555 (27·6%)	3131/11 964 (26·2%)	391/1520 (25·7%)
Obesity		7008/34 893 (20·1%)	7846/39 170 (20·0%)	1217/10 484 (11·6%)	222/1369 (16·2%)
Chronic neurological disease		3460/38 932 (8·9%)	3992/43 848 (9·1%)	1696/12 051 (14·1%)	143/1543 (9·3%)
Dementia		2989/38 847 (7·7%)	3472/43 763 (7·9%)	2058/11 920 (17·3%)	122/1527 (8·0%)
Chronic kidney disease		5329/39 017 (13·7%)	6059/43 943 (13·8%)	2506/12 119 (20·7%)	219/1553 (14·1%)
Moderate or severe liver disease		490/38 802 (1·3%)	573/43 711 (1·3%)	315/11 984 (2·6%)	24/1536 (1·6%)
Mild liver disease		583/38 697 (1·5%)	661/43 578 (1·5%)	238/11 930 (2·0%)	27/1534 (1·8%)
Malignancy		3017/38 938 (7·7%)	3627/43 860 (8·3%)	1518/12 041 (12·6%)	123/1542 (8·0%)
Rheumatological disease		4339/38 828 (11·2%)	4982/43 754 (11·4%)	1788/11 998 (14·9%)	149/1529 (9·7%)
Pre-admission immunosuppressants, including corticosteroids		4288/39 393 (10·9%)	5264/44 314 (11·9%)	1075/12 131 (8·9%)	134/1419 (9·4%)
HIV/AIDS		127/38 118 (0·3%)	155/42 942 (0·4%)	45/11 742 (0·4%)	3/1510 (0·2%)

Data are n (%), median (IQR), or n/N %). Percentages are from complete cases and read vertically. Missing data from explanatory variables are omitted here and included in the appendix (pp 11–15). Percentages might not sum to 100 due to rounding. NA=not applicable (0 patients or suppressed).

* The any corticosteroid group includes 42 442 (88·8%) of 47 795 patients who received oral or intravenous dexamethasone 6 mg once daily, 3895 (8·1%) who received another or unknown dose or frequency of dexamethasone, 509 (1·1%) who received hydrocortisone, 406 (0·8%) who received prednisolone, and 24 (0·1%) who received methylprednisolone.

† A score of 15 indicates a fully awake state, and a score of less than 15 indicates any deficit in either the eye, motor, or verbal response used to assess conscious level.

Patients receiving any corticosteroid had a higher CRP than patients who did not receive corticosteroids, among other severity markers on admission (table 1). Patients receiving corticosteroids were more likely to have asthma and obesity, but less likely to have most other comorbidities, including chronic cardiac disease, chronic kidney disease, dementia, and chronic neurological disease (table 1). Of 44 314 patients receiving any corticosteroid, 5264 (11·9%) received some immunosuppressant medication (eg, oral corticosteroids or disease-modifying antirheumatic drugs) pre-admission (table 1).

Corticosteroid administration was higher among patients admitted to critical care (11 185 [86·6%] of 12 909) or requiring invasive ventilation (4882 [85·2%] of 5730) than in patients receiving ward-level care (36 415 [72·4%] of 50 278; figure 2; appendix pp 11–15). Of 27 226 patients not recorded to have received supplementary oxygen at any point during the hospital stay 4173 (15·3%) received corticosteroids (appendix pp 6–10).

Corticosteroid administration increased with higher COVID-19 severity: 9757 (58·8%) of 16 593 patients with mild COVID-19, 21 165 (79·0%) of 26 776 patients with moderate COVID-19, and 16 548 (84·4%) of 19 600 patients with severe COVID-19 received corticosteroids (figure 1)*.*

Among 46 376 patients with moderate or severe COVID-19, 37 713 (81·3%) received any corticosteroid. Patients not receiving corticosteroids were older than those receiving corticosteroids (table 2). As among patients who received supplementary oxygen, patients with moderate or severe COVID-19 who had any corticosteroid had lower frequencies of chronic heart disease, chronic kidney disease, chronic neurological disease, and dementia than those who had no corticosteroid. In addition, of 591 patients with moderate or severe liver disease, 421 (71·2%) received corticosteroids. Rates of corticosteroid administration ranged from 4735 (74·8%) of 6333 patients in South East NHS regions to 3126 (88·1%) of 3550 patients in London NHS regions (table 2).

**Table 2:** Patients who received oxygen at any point in their admission and classified as having moderate or severe COVID-19 between June 17, 2020, and April 14, 2021, stratified by corticosteroid administration

		Any corticosteroid group*(n=37 713)	**No corticosteroid group (n=7000)**	**Missing corticosteroid group (n=1663)**
**Patient demographics**				
PCR-positive COVID-19		34 158/41 212 (82·9%)	6259/41 212 (15·2%)	795/41 212 (1·9%)
Age on admission, years (n=46 376)		67·3 (55·4–78·5)	78·0 (66·0–86·0)	69·6 (56·4–79·7)
Age, years				
	<50	6006/6867 (87·5%)	619/6867 (9·0%)	242/6867 (3·5%)
	50–59	7037/7951 (88·5%)	626/7951 (7·9%)	288/7951 (3·6%)
	60–69	7807/9041 (86·4%)	922/9041 (10·2%)	312/9041 (3·5%)
	70–79	8543/10 690 (79·9%)	1733/10 690 (16·2%)	414/10 690 (3·9%)
	≥80	8320/11 827 (70·3%)	3100/11 827 (26·2%)	407/11 827 (3·4%)
Sex				
	Female	15 141/19 125 (79·2%)	3317/19 125 (17·3%)	667/19 125 (3·5%)
	Male	22 518/27 186 (82·8%)	3674/27 186 (13·5%)	994/27 186 (3·7%)
Ethnicity				
	White	26 261/32 734 (80·2%)	5458/32 734 (16·7%)	1015/32 734 (3·1%)
	South Asian	2793/3119 (89·5%)	280/3119 (9·0%)	46/3119 (1·5%)
	East Asian	209/246 (85·0%)	28/246 (11·4%)	9/246 (3·7%)
	Black	912/1044 (87·4%)	100/1044 (9·6%)	32/1044 (3·1%)
	Other ethnic minority	2606/3020 (86·3%)	298/3020 (9·9%)	116/3020 (3·8%)
Pregnant (only recorded for women aged 12–55 years)				
	No	3159/3597 (87·8%)	328/3597 (9·1%)	110/3597 (3·1%)
	Yes	84/155 (54·2%)	61/155 (39·4%)	10/155 (6·5%)
NHS region				
	East of England	3728/4765 (78·2%)	918/4765 (19·3%)	119/4765 (2·5%)
	London	3126/3550 (88·1%)	331/3550 (9·3%)	93/3550 (2·6%)
	Midlands	8021/9888 (81·1%)	1648/9888 (16·7%)	219/9888 (2·2%)
	North East and Yorkshire	5997/7132 (84·1%)	984/7132 (13·8%)	151/7132 (2·1%)
	Northern Ireland	50/52 (96·2%)	NA	NA
	North West	7146/8661 (82·5%)	1269/8661 (14·7%)	246/8661 (2·8%)
	Scotland	759/914 (83·0%)	97/914 (10·6%)	58/914 (6·3%)
	South East	4735/6333 (74·8%)	1012/6333 (16·0%)	586/6333 (9·3%)
	South West	2862/3507 (81·6%)	487/3507 (13·9%)	158/3507 (4·5%)
	Wales	1044/1265 (82·5%)	210/1265 (16·6%)	11/1265 (0·9%)
**Severity of illness at admission**				
Oxygen saturation (SpO_2_), % (n=46 217)		91·0% (88·0–93·0)	92·0% (89·0–94·0)	91·0% (88·0–93·0)
Respiratory rate, breaths per min (n=44 608)		24·0 (21·0–28·0)	23·0 (20·0–28·0)	24·0 (20·0–28·0)
C-reactive protein, mg/dL (n=38 643)		99·0 (53·0–164·0)	63·0 (24·3–127·0)	96·0 (47·0–163·0)
Blood urea nitrogen, mg/dL (n=38 396)		6·6 (4·8–9·9)	7·7 (5·2–12·1)	7·0 (4·9–10·6)
Glasgow coma scale†				
	15	33 136/40 165 (82·5%)	5643/40 165 (14·0%)	1386/40 165 (3·5%)
	<15	3198/4375 (73·1%)	1003/4375 (22·9%)	174/4375 (4·0%)
Highest fraction of inspired oxygen (FiO_2_) (n=43 696)		0·32 (0·24–0·60)	0·24 (0·21–0·32)	0·32 (0·24–0·60)
**Comorbidities**				
Any comorbidity				
	No	5957/6760 (88·1%)	587/6760 (8·7%)	216/6760 (3·2%)
	Yes	30 253/37 425 (80·8%)	6187/37 425 (16·5%)	985/37 425 (2·6%)
	Hypertension	16 382/20 242 (80·9%)	3371/20 242 (16·7%)	489/20 242 (2·4%)
	Chronic cardiac disease	9105/11 967 (76·1%)	2578/11 967 (21·5%)	284/11 967 (2·4%)
	Non-asthmatic chronic pulmonary disease	6668/8599 (77·5%)	1718/8599 (20·0%)	213/8599 (2·5%)
	Asthma	5953/7072 (84·2%)	947/7072 (13·4%)	172/7072 (2·4%)
	Type 1 diabetes	693/875 (79·2%)	145/875 (16·6%)	37/875 (4·2%)
	Type 2 diabetes	9645/11 731 (82·2%)	1798/11 731 (15·3%)	288/11 731 (2·5%)
Obesity		6674/7563 (88·2%)	726/7563 (9·6%)	163/7563 (2·2%)
	Chronic neurological disease	2985/4000 (74·6%)	919/4000 (23·0%)	96/4000 (2·4%)
	Dementia	2559/3788 (67·6%)	1144/3788 (30·2%)	85/3788 (2·2%)
	Chronic kidney disease	4617/6134 (75·3%)	1366/6134 (22·3%)	151/6134 (2·5%)
	Mild liver disease	522/645 (80·9%)	105/645 (16·3%)	18/645 (2·8%)
	Moderate or severe liver disease	421/591 (71·2%)	154/591 (26·1%)	16/591 (2·7%)
	Malignancy	2742/3615 (75·9%)	795/3615 (22·0%)	78/3615 (2·2%)
	Rheumatological disease	3869/4960 (78·0%)	974/4960 (19·6%)	117/4960 (2·4%)
	Pre-admission immunosuppressants, including corticosteroids	4299/5079 (84·6%)	678/5079 (13·3%)	102/5079 (2·0%)
	HIV/AIDS	117/141 (83·0%)	21/141 (14·9%)	<5/141 (<5%)

Data are n/N (%) or median (IQR). Proportions read horizontally. Percentages might not sum to 100 due to rounding.

* The any corticosteroid group includes 33 580 (89·0%) of 33 713 patients who received oral or intravenous dexamethasone 6 mg once daily, 3366 (8·9%) who received another or unknown dose or frequency of dexamethasone, 392 (1·0%) who received hydrocortisone, 353 (0·9%) who received prednisolone, and 22 (0·1%) who received methylprednisolone.

† A score of 15 indicates a fully awake state, and a score of less than 15 indicates any deficit in either the eye, motor, or verbal response used to assess conscious level.

**Figure 4:**
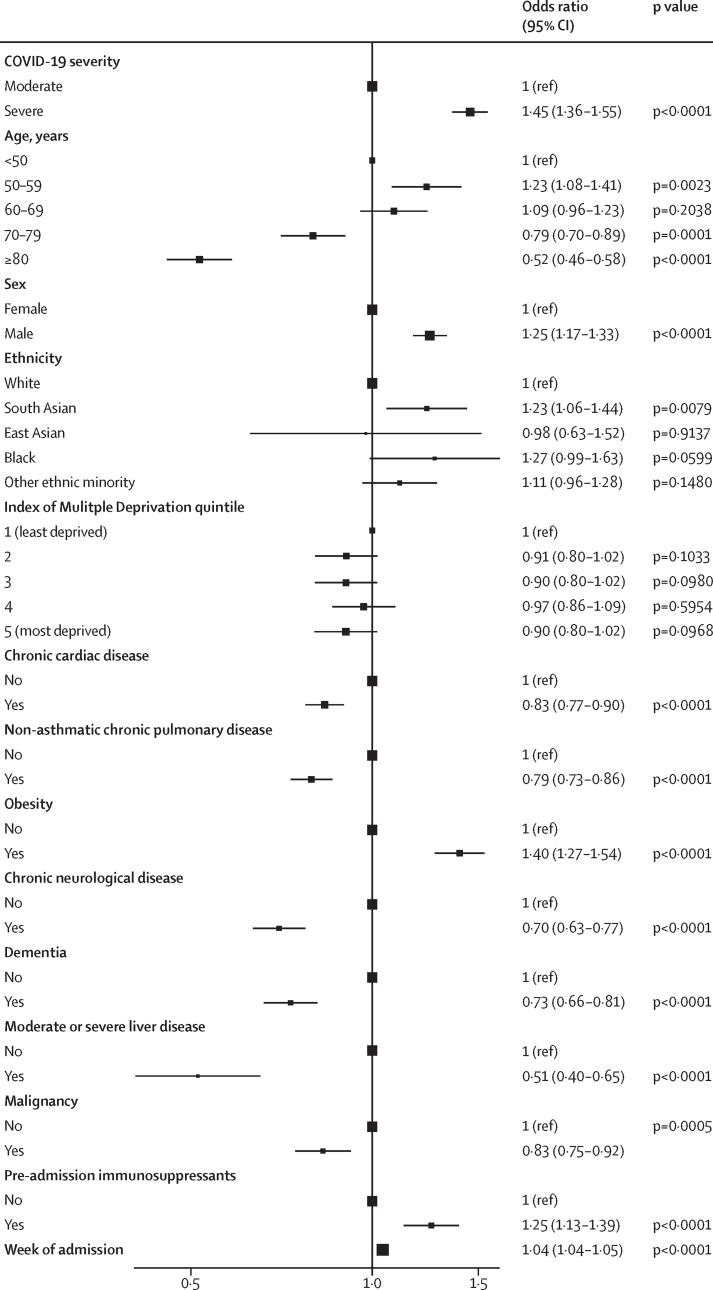
Multivariable multilevel regression model of any corticosteroid administration among 30 670 patients with moderate or severe COVID-19 receiving supplementary oxygen and admitted to hospital between June 16, 2020, and April 14, 2021 All variables included in the univariable and multivariable multilevel model are shown in the appendix (pp 32–34).

Univariable and multivariable associations with corticosteroid administration are described in the appendix (pp 32–34). After adjusting for sex, deprivation quintile, ethnicity, comorbidities, and admitting hospital, increasing age was strongly associated with patients not receiving corticosteroids (figure 4). Other factors associated with lower rates of corticosteroid administration included chronic cardiac disease, non-asthmatic chronic pulmonary disease, chronic neurological disease, dementia, moderate or severe liver disease, and malignancy. We found no association with diabetes and rate of corticosteroid administration. Severe illness, male sex, obesity, and pre-admission immunosuppressants were associated with higher rates of corticosteroid administration. No relevant first-order interactions were identified or incorporated into the models. Week of admission (both linear and quadratic term) did not contribute significantly. C statistic, as a measure of model discrimination, was 0·743 (95% CI 0·736–0·751).

These results were consistent in the sensitivity analyses in which we excluded patients with missing corticosteroid (appendix pp 35–37), in patients with a recorded PCR-positive COVID-19 (appendix pp 38–40), and in which we excluded patients with death or palliative discharge within 2 days of admission (appendix pp 41–43). For the subgroup of 16 902 patients with recorded clinical frailty, significant associations with lower rates of corticosteroid prescribing included age 80 years or older (adjusted OR 0·80 [95% CI 0·66–0·97], p=0·021), increasing clinical frailty, and the aforementioned comorbidities (appendix pp 44–48).

Corticosteroids were administered to 8320 (70·3%) of 11 827 patients aged 80 years or older with moderate or severe COVID-19 (table 3). Rates of corticosteroid administration varied across the NHS regions from 1071 (61·0%) of 1756 patients in NHS South East to 416 (80·3%) of 518 patients in NHS London (table 3). Rates increased with level of care: 6381 (68·0%) of 9387 patients on oxygen alone, 1320 (85·4%) of 1546 patients on non-invasive ventilation, 456 (85·4%) of 534 patients in critical care, and 64 (82·1%) of 78 patients invasively ventilated. Within this older cohort, the proportion receiving corticosteroids decreased with increasing Clinical Frailty Scale^17^ score, but less so with comorbidities: the pattern in specific comorbidities mirrored the overall cohort.

**Table 3:** Subgroup analysis of patients aged 80 years or older with moderate or severe COVID-19, admitted to hospital between June 17, 2020, and April 14, 2021

		Any corticosteroid group*(n=8320)	**No corticosteroid group (n=3100)**	**Missing corticosteroid group (n=407)**
**Patient demographics**				
Age on admission, years (n=11 827)		85·7 (82·8–89·5)	86·9 (83·5–90·8)	86·4 (83·6–90·3)
Sex				
	Female	3865/5699 (67·8%)	1623/5699 (28·5%)	211/5699 (3·7%)
	Male	4442/6111 (72·7%)	1473/6111 (24·1%)	196/6111 (3·2%)
Ethnicity				
	White	6666/9556 (69·8%)	2607/9556 (27·3%)	283/9556 (3·0%)
	South Asian	328/389 (84·3%)	59/389 (15·2%)	2/389 (0·5%)
	East Asian	26/31 (83·9%)	4/31 (12·9%)	1/31 (3·2%)
	Black	97/123 (78·9%)	24/123 (19·5%)	2/123 (1·6%)
	Other ethnic minority	323/420 (76·9%)	85/420 (20·2%)	12/420 (2·9%)
NHS region				
	East of England	1026/1490 (68·9%)	436/1490 (29·3%)	28/1490 (1·9%)
	London	416/518 (80·3%)	96/518 (18·5%)	6/518 (1·2%)
	Midlands	1900/2692 (70·6%)	734/2692 (27·3%)	58/2692 (2·2%)
	North East and Yorkshire	1379/1901 (72·5%)	491/1901 (25·8%)	31/1901 (1·6%)
	North West	1462/2004 (73·0%)	490/2004 (24·5%)	52/2004 (2·6%)
	Scotland	126/168 (75·0%)	34/168 (20·2%)	8/168 (4·8%)
	South East	1071/1756 (61·0%)	508/1756 (28·9%)	177/1756 (10·1%)
	South West	649/905 (71·7%)	211/905 (23·3%)	45/905 (5·0%)
	Wales	227/310 (73·2%)	82/310 (26·5%)	1/310 (0·3%)
**Severity of illness on admission**				
Oxygen saturation (SpO_2_), % (n=11 809)		92·0% (88·0–94·0)	92·0% (89·0–94·0)	92·0% (88·0–93·0)
Respiratory rate, breaths per min (n=11 409)		24·0 (20·0–28·0)	22·0 (20·0–27·0)	24·0 (20·0–27·0)
C-reactive protein, mg/dL (n=9346)		89·0 (45·0–152·0)	60·2 (24·0–123·0)	77·0 (35·0–135·9)
Blood urea nitrogen, mg/dL (n=9408)		9·6 (6·9–13·9)	9·5 (6·7–14·8)	9·4 (6·6–14·3)
Glasgow coma scale†				
	15	6722/9453 (71·1%)	2409/9453 (25·5%)	322/9453 (3·4%)
	<15	1275/1903 (67·0%)	562/1903 (29·5%)	66/1903 (3·5%)
Highest fraction of inspired oxygen (FiO_2_; n=10 984)		0·28 (0·24–0·40)	0·24 (0·21–0·32)	0·32 (0·24–0·40)
Severity criteria				
	Moderate COVID-19	4841/7182 (67·4%)	2076/7182 (28·9%)	265/7182 (3·7%)
	Severe COVID-19	3479/4645 (74·9%)	1024/4645 (22·0%)	142/4645 (3·1%)
**Comorbidities**				
Any comorbidity				
	No	343/454 (75·6%)	92/454 (20·3%)	19/454 (4·2%)
	Yes	7697/10 868 (70·8%)	2921/10 868 (26·9%)	250/10 868 (2·3%)
	Hypertension	4848/6742 (71·9%)	1762/6742 (26·1%)	132/6742 (2·0%)
	Chronic cardiac disease	3908/5568 (70·2%)	1551/5568 (27·9%)	109/5568 (2·0%)
	Non-asthmatic chronic pulmonary disease	2095/2925 (71·6%)	762/2925 (26·1%)	68/2925 (2·3%)
	Asthma	1000/1369 (73·0%)	337/1369 (24·6%)	32/1369 (2·3%)
	Type 1 diabetes	133/181 (73·5%)	41/181 (22·7%)	7/181 (3·9%)
	Type 2 diabetes	2183/3001 (72·7%)	756/3001 (25·2%)	62/3001 (2·1%)
	Obesity	458/611 (75·0%)	139/611 (22·7%)	14/611 (2·3%)
	Chronic neurological disease	961/1433 (67·1%)	440/1433 (30·7%)	32/1433 (2·2%)
	Dementia	1755/2681 (65·5%)	866/2681 (32·3%)	60/2681 (2·2%)
	Chronic kidney disease	2281/3205 (71·2%)	852/3205 (26·6%)	72/3205 (2·2%)
	Mild liver disease	66/93 (71·0%)	27/93 (29·0%)	0
	Moderate or severe liver disease	62/102 (60·8%)	36/102 (35·3%)	4/102 (3·9%)
	Malignancy	1017/1467 (69·3%)	414/1467 (28·2%)	36/1467 (2·5%)
	Rheumatological disease	1295/1900 (68·2%)	561/1900 (29·5%)	44/1900 (2·3%)
	Pre-admission immunosuppressants, including corticosteroids	906/1163 (77·9%)	228/1163 (19·6%)	29/1163 (2·5%)
	HIV/AIDS	14/21 (66·7%)	7/21 (33·3%)	0
Clinical frailty‡				
	1–2	164/196 (83·7%)	26/196 (13·3%)	6/196 (3·1%)
	3–4	1207/1539 (78·4%)	300/1539 (19·5%)	32/1539 (2·1%)
	5–6	2082/2885 (72·2%)	753/2885 (26·1%)	50/2885 (1·7%)
	7–9	1250/1938 (64·5%)	646/1938 (33·3%)	42/1938 (2·2%)
	Missing	3617/5269 (68·6%)	1375/5269 (26·1%)	277/5269 (5·3%)
**Level of care and escalation plans**				
Level of respiratory support				
	Oxygen alone	6381/9387 (68·0%)	2669/9387 (28·4%)	337/9387 (3·6%)
	High-flow nasal cannula	555/816 (68·0%)	218/816 (26·7%)	43/816 (5·3%)
	Non-invasive ventilation	1320/1546 (85·4%)	201/1546 (13·0%)	25/1546 (1·6%)
	Invasive ventilation	64/78 (82·1%)	12/78 (15·4%)	2/78 (2·6%)
Critical care admission				
	Yes	456/534 (85·4%)	65/78 (12·2%)	13/78 (2·4%)
	No	..	..	..
	Not indicated	3630/5176 (70·1%)	1514/5176 (29·3%)	32/5176 (0·6%)
	Not appropriate	3310/4308 (76·8%)	985/4308 (22·9%)	13/4308 (0·3%)
	Missing	1380/2343 (58·9%)	601/2343 (25·7%)	362/2343 (15·5%)
Death within 2 days of admission				
	Death or palliative discharge within 2 days of admission	407/600 (67·8%)	175/600 (29·2%)	18/600 (3·0%)
	Longer length of hospital stay or survival	7913/11 227 (70·5%)	2925/11 227 (26·1%)	389/11 227 (3·5%)

Data are median (IQR), n/N (%), or n (%). Proportions read horizontally. Proportions read horizontally. Percentages might not sum to 100 due to rounding.

* The any corticosteroid group includes 7305 (87·8%) of 8320 patients who received oral or intravenous dexamethasone 6 mg once daily, 780 (9·4%) who received another or unknown dose or frequency of dexamethasone, 120 (1·4%) who received hydrocortisone, 114 (1·4%) who received prednisolone, and fewer than five who received methylprednisolone.

† A score of 15 indicates a fully awake state, and a score of less than 15 indicates any deficit in either the eye, motor, or verbal response used to assess conscious level.

‡ Clinical frailty reference: scores of 1–2 indicate fit, 3–4 indicate vulnerable, but not frail, 5–6 indicate initial signs of frailty but with some degree of independence, and 7–9 indicate severe or very severe frailty.

Among 4308 in this cohort in whom critical care admission was not considered appropriate, 3310 (76·8%) received corticosteroids. Fewer patients with a Glasgow Coma Scale score of less than 15 received corticosteroid therapy than those with a score of 15 (1275 [67·0%] of 1903 *vs* 6722 [71·1%] of 9453). Of the 11 827 patients aged 80 years or older, 600 (5·1%) died or were deemed palliative within 2 days of hospital admission. These patients had slightly lower rates of corticosteroid administration than those with a longer hospital stay or who survived (407 [67·8%] of 600 *vs* 7913 [70·5%] of 11 227).

Corticosteroid administration rates were lower in pregnant patients with moderate or severe COVID-19 compared with non-pregnant women with moderate or severe COVID-19 aged 18–55 years (84 [54·2%] of 155 *vs* 3159 [87·8%] of 3597). Pregnant and non-pregnant patients with moderate or severe COVID-19 were similar regarding proportions of symptomatic versus asymptomatic patients and moderate versus severe COVID-19 (appendix pp 30–31).

## Discussion

We observed a rapid and significant increase in the administration of corticosteroids since publication of the RECOVERY trial results on June 16, 2020. However, not all patients who fulfilled the criteria of oxygen therapy and additional severity criteria received corticosteroids. Ages of 70 years or older were associated with consistently lower rates of corticosteroid administration. Patients with dementia, chronic neurological disease, chronic cardiac disease, moderate or severe liver disease, and pregnant patients were also less likely to receive corticosteroids, regardless of severity of illness.

Considering the knowing–doing gap between evidence and implementation, which gets averaged at 17 years,^21^ the implementation of this programme has been rapid. This fast implementation could have been facilitated by the timing of the evidence, communication, the simplicity of the intervention, and the global focus on the COVID-19 pandemic. In June, 2020, in-hospital mortality from COVID-19 was around 20–25% overall, and up to 38% among patients receiving invasive mechanical ventilation,^22^ with no proven treatments to reduce mortality. For clinicians, corticosteroids are widely used across hospitals and specialties, and their administration is simple, and the widespread coverage of the trial results in the UK media might have helped to make corticosteroids acceptable among patients. This perceived high benefit-to-risk ratio, familiarity, and simplicity might have aided the achievement of the accepted 80–90% target reliability.^23^

However, this target was not achieved universally. Age of 70 years or older, clinical frailty, and the aforementioned comorbidities were associated with lower rates of corticosteroid administration. There were also regional differences in corticosteroid uptake. In December, 2020, there seemed to be a reduction in corticosteroid prescribing, which could reflect the increase in hospital burden of patients with COVID-19. These discrepancies highlight the importance of process mapping and exploring potential barriers in addition to quantifying rate of change, to better understand the implementation process.^24^

Clinicians might have been hesitant to prescribe corticosteroids in patients with advanced age or multiple comorbidities, especially if care was primarily palliative. We found a significant reduction in corticosteroid administration among patients who were 70 years or older, independent of patient demographics, illness severity, and comorbidities, which persisted when adjusting for frailty in the smaller cohort of patients for whom frailty was recorded. However, we did not find that treatment escalation plans affected the administration of corticosteroids or that patients who died in the first 2 days of hospital admission, or who were potentially on a palliative pathway, had lower rates of corticosteroids prescribed.

Other reasons for lower rates of corticosteroid prescribing in patients aged 70 years or older could include concerns of cognitive adverse effects and initial limited evidence in the RECOVERY trial subgroup analysis,^3^ although the REACT meta-analysis showed benefit in patients older than 60 years.^6^ The known corticosteroid-induced effects on cognition, delirium, and agitation, might have led clinicians to be cautious when prescribing them for patients with pre-existing cognitive decline or delirium.^25^ As the prevalence of delirium increases with age,^26^ clinicians might perceive patients older than 70 years to be at higher risk of such adverse effects. Although delirium was not recorded in this dataset, a Glasgow coma score of less than 15 was associated with lower rates of corticosteroid administration in patients with moderate or severe COVID-19 and in the subgroup of patients aged 80 years or older with moderate or severe COVID-19. This strong independent association of older age and lower rates of corticosteroid administration should be further explored.

Concerns over safety and scarce evidence could also disadvantage patients with diabetes^27^ and pregnant patients. Although we did not see significant differences in corticosteroid administration associated with diabetes, the uptake in pregnant patients was considerably lower than in non-pregnant women of reproductive age. This result could be explained by the limited and changing COVID-19 guidance for the use of corticosteroids in pregnancy.^5^, ^28^ Although the most recent guidance from the Royal College of Obstetricians and Gynaecologists recommends use of corticosteroids in pregnant patients with COVID-19 requiring oxygen supplementation or ventilation,^29^ the initial concern of harm and controversies around the ideal corticosteroid^30^ might have compromised effective implementation. Although there might be a lower threshold to admit pregnant patients resulting in a less unwell cohort overall, the persistent poor uptake in pregnant patients with markers of high illness severity warrants further exploration.

Pre-admission immunosuppressants were associated with higher rates of corticosteroid administration, potentially reflecting indications for pre-existing conditions or lower thresholds to start corticosteroids. Some pre-existing comorbidities for which corticosteroids are occasionally indicated, such as malignancy or rheumatological conditions, were associated with lower rates of corticosteroid administration, which could reflect proven or potential concern about associated immunosuppression. Immunosuppression might present a relative contraindication to corticosteroids; therefore, the risk of secondary infection with corticosteroids should be explored further to ensure safe and appropriate prescribing.^31^ Alternatively, clinicians might perceive there to be less benefit from corticosteroids because of a potential effect of existing immunosuppression.

Corticosteroids are also indicated in exacerbations of asthma and other chronic pulmonary diseases. However, patients with moderate or severe COVID-19 and non-asthmatic chronic pulmonary disease received fewer corticosteroids, which persisted after adjusting for age and comorbidities. This finding could reflect lower baseline SpO_2_ and higher respiratory rates resulting in misclassification of some patients into artificially higher severity groups.

The rates of corticosteroid administration increased with markers of severity, such as high respiratory rate, low SpO_2_, and high CRP, and increasing level of care, but corticosteroids were also administered to patients not recorded to have received supplementary oxygen or without markers of severe COVID-19. Despite the WHO severity criteria,^7^ the initial clinical guidelines and the principal message from the RECOVERY trial recommended corticosteroids to anyone needing supplementary oxygen,^5^ and this message might have persisted as a simple decision aid. Contrarily, both the RECOVERY and the Metcovid trials reported a potential signal to harm among patients not receiving oxygen,^3^, ^32^ which might have persuaded clinicians to err to the side of caution among patients with less severe COVID-19. Despite this signal to harm among patients receiving no oxygen, the rate of corticosteroid administration in the non-oxygen cohort observed here did not change after RECOVERY trial publication. To account for the less clear indication for patients with milder disease, we focused on patients with moderate or severe COVID-19 when identifying the discussed factors independently associated with lower corticosteroid administration.

The comprehensive clinical data collected allowed detailed analysis of subgroups to explore administration of corticosteroids. However, there are some limitations. There were more missing data than would be expected for a prospective cohort study, due to the nature of the pandemic, but this issue was handled using appropriate methods. Although the ISARIC WHO CCP-UK study captured a third of the patients admitted to hospital with COVID-19 in the UK after June 16, 2020, and therefore offers a generalisable estimate of the national uptake of corticosteroids, selection bias might still exist. Fewer hospital admissions before June 16 could be partly contributing to the slight premature increase in corticosteroid administration. We cannot comment on the duration of corticosteroid treatment or supplementary oxygen, or the time to prescription. We were also unable to determine the type of non-critical care ward, which could contribute to differences in rates of corticosteroid prescribing due to a range of skills, preparedness, or knowledge in treating patients with COVID-19. As the indication for the corticosteroid therapy was not recorded, a proportion of patients might have received corticosteroids for other indications; therefore, our corticosteroid administration rates for patients with COVID-19 are probably overestimates of the true administration rate for COVID-19. Similarly, cautions with corticosteroids, such as peptic ulcer disease or risk of gastrointestinal bleeding,^33^ were not collected. Although the severity criteria were robust, derived from commonly used clinical markers on admission, and reflected the current clinical guidelines, they could not capture all criteria for severe or critical COVID-19,^2^ such as clinical trend. Finally, there might be unmeasured confounders that we were either unaware of or unable to measure.

Rates of corticosteroid administration in COVID-19 have increased substantially since the publication of the RECOVERY trial results and updated clinical guidelines, with the greatest rates seen among patients with higher severity of illness. However, this study highlighted a marked difference in corticosteroid uptake based on age, with patients aged 70 years or older much less likely to receive corticosteroids, even after accounting for illness severity, comorbidities, and clinical frailty. The presence of chronic cardiac disease, chronic neurological disease, dementia, or pregnancy was also associated with lower rates of corticosteroid administration. A decision not to administer a cheap, simple, and potentially life-saving therapy such as low-dose corticosteroids in COVID-19 might benefit from a consensus approach, whereby any decision not to adhere to a guideline requires discussion and sense checking with another clinical colleague. This approach might provide reassurance to patients of equitable access and support clinicians in potentially challenging decision making. Future qualitative research should evaluate whether there were systematic barriers or enablers to implementation, overall or in specific subgroups of patients, across a range of institutions, settings, and practitioners. Such work should aim to understand to what extent the results presented here represent appropriate clinical judgement or potentially modifiable barriers to receiving life-saving treatments.

## Data sharing

Data, protocols, and all documentation around this analysis will be made available to academic researchers after authorisation from the ISARIC independent data management and access committee.

## Declaration of interests

ABD reports grants from the UK Department of Health and Social Care (DHSC) during the conduct of the study, and grants from the Wellcome Trust outside the submitted work. JSN-V-T reports grants from the DHSC during the conduct of the study, and is seconded to the DHSC. PJMO reports personal fees from consultancies and from the European Respiratory Society; grants from the UK Medical Research Council (MRC), the MRC Global Challenge Research Fund, the EU, the NIHR Biomedical Research Centre, MRC–GSK, the Wellcome Trust, and the NIHR (Health Protection Research Unit in Respiratory Infections at Imperial College London); and is an NIHR senior investigator outside the submitted work; his role as President of the British Society for Immunology was unpaid but travel and accommodation at some meetings was provided by the society. JKB reports grants from the MRC. MGS reports grants from DHSC, NIHR UK, MRC UK, HPRU in Emerging and Zoonotic Infections, and University of Liverpool, during the conduct of the study; and is chair of the Infectious Diseases Science Advisory Board and minority shareholder of Integrum Scientific, Greensboro NC, and Independent external and non-remunerated member of Pfizer's External Data Monitoring Committee for their mRNA vaccine program(s) outside the submitted work. All other authors declare no competing interests.
