# Supplementary material for: Implementation of corticosteroids in treatment of COVID-19 in the ISARIC WHO Clinical Characterisation Protocol UK: prospective, cohort study
Source: Lancet Digit Health. 2022 Mar 22;4(4):e220–34. doi: 10.1016/S2589-7500(22)00018-8 (PMC8940185; doi:10.1016/S2589-7500(22)00018-8)

THE LANCET
Digital Health

Supplementary appendix

This appendix formed part of the original submission and has been peer reviewed. We post it as supplied by the authors.

Supplement to: Närhi F, Moonesinghe SR, Shenkin SD, et al. Implementation of corticosteroids in treatment of COVID-19 in the ISARIC WHO Clinical Characterisation Protocol UK: prospective, cohort study. *Lancet Digit Health* 2022; **4:** e220–34.

Online supplement

Figure S1 Timeline of evidence and guideline changes.[1–5]

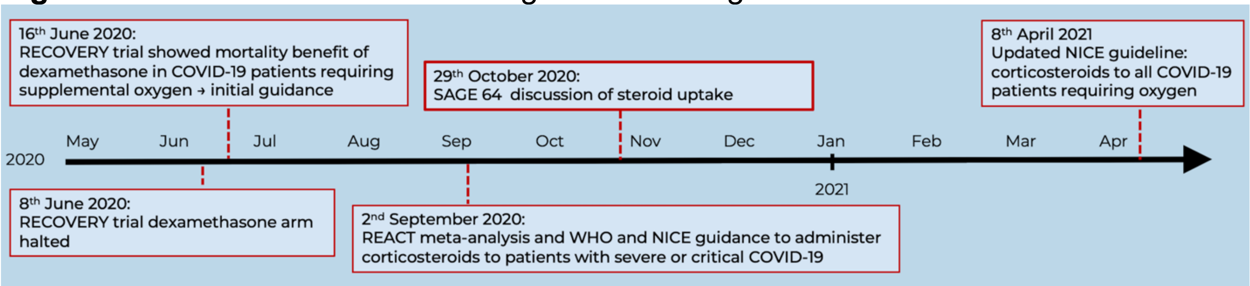

Exclusions

Only acute care hospitals were included. Community hospitals and mental health facilities were excluded given the different population characteristics. We only considered community acquired infection, which was assumed by symptom onset 5 days or less after admission to hospital.
We have included patients with both PCR positive COVID-19 and patients with high likelihood of disease if supporting PCR results were not available.

Variables

Any treatments and respiratory support were recorded at multiple time points in the admission (day 1, day 3, day 6, day 9, day of any admission to critical care and discharge), which aided identification of patients who received any supplementary oxygen as well as further characterisation of the cohort. Highest level of respiratory support was made as a categorical variable, where invasive ventilation (IMV), non-invasive ventilation (NIV), high-flow nasal cannula (HFNC), oxygen only and no oxygen were hierarchical. The ISARIC 4C case report form collected information on whether or not patient had an admission to an intensive care or high dependency unit (these were not separated). This was used to assign the highest level of care where patients with a recorded admission to intensive care/high dependency unit were assigned “critical care”, and those with no such admission were considered to be cared for in the ward. We further categorised patients in critical care by receipt of invasive ventilation. The any oxygen variable represents patients who were recorded to receive any supplementary oxygen, any invasive ventilation (this was assumed to involve supplementary oxygen) or have daily fraction of inspired oxygen above 0.21 on any day of admission.
Physiological parameters of the ISARIC 4C mortality score for COVID-19[6], including peripheral oxygen saturations, respiratory rate, c-reactive protein (CRP), blood urea nitrogen (BUN) and Glasgow Coma Scale (GCS)) recorded on the day of hospital admission were used as markers of illness severity.

Corticosteroid treatment was recorded as a free text entry, which was interpreted using an algorithm that matches the free text to standardised, generic medication names. Mapping the medications to drug classes, we were able to filter corticosteroids specifically for systemic use.

Clinical frailty score was recorded by the research team in the ISARIC 4C case report form from August 2020 onwards with reference to the Dalhousie University Clinical Frailty Score.[7,8] The clinical frailty score was further collapsed to 1-2 (fit), 3-4 (vulnerable, but not frail), 5-6 (initial signs of frailty but with some degree of independence) and 7-9 (severe or very severe frailty).

Deprivation was derived by mapping the patients' post-codes to their corresponding Index of Multiple Deprivation (IMD) using the Office for National Statistics postcode data. Deprivation quintiles were calculated from national data, with the convention of first quintile being the least deprived and the fifth quintile the most deprived. For patients where postcodes were missing, the average IMD rank, weighted by population in each lower super output area for a given hospital catchment area, was used.

Any intensive care or high dependency unit admission during hospitalisation was recorded as yes, no or not known. If "no", reasons "Not indicated" or "Not appropriate" could be given. "Not appropriate" was specified as "Advanced care plan/discussion documented in notes regarding not for escalation of care beyond ward".

Age was categorised into <50 years, 50-59 years, 60-69 years, 70-79 years and 80+, corresponding to the ISARIC4C mortality score categories[6].

Any comorbidity variable represented the presence of any of: hypertension, chronic cardiac disease non-asthmatic chronic pulmonary disease, asthma, type 1 diabetes, type 2 diabetes, obesity, chronic neurological disease, dementia, chronic kidney disease, moderate or severe liver disease, mild liver disease, malignancy, rheumatologic disease, pre-admission immunosuppressants including corticosteroids and/or AIDS/HIV. Comorbidity indicated "unknown" was considered no comorbidity. If all of these comorbidities were missing, any comorbidity was also considered missing.

Patients were recorded to have symptoms at admission if they reported cough, fever, sore throat, runny nose, ear pain, wheezing, chest pain, myalgia, joint pain, fatigue, shortness of breath, disturbance or loss of taste, lower chest wall indrawing, headache, altered consciousness/confusion, seizures, abdominal pain, vomiting/nausea, diarrhoea, conjunctivitis, skin rash, skin ulcers, lymphadenopathy, bleeding and/or anosmia. If none of these were reported, patients were considered asymptomatic.

Admitting hospitals were mapped to the NHS regions to create the NHS region variable. These are reported for the patients with moderate or severe COVID-19 to account for the differences in patient cohort severity and thus eligibility for corticosteroid therapy. (Tables S3-S5)

Missing data

We checked that the data was coded correctly, which identified the potential erroneous coding of all patients in some hospitals with an intensive care unit admission. We excluded these patients from the analysis as per the consort diagram. We identified missing values within each variable and analysed the patterns of missingness with the

finalfit package. We visually checked for associations between missing and observed data.

For patients where postcodes were missing, we used the average IMD rank, weighted by population in each lower super output area for a given hospital catchment area. We considered comorbidity indicated “unknown” as no comorbidity. We considered corticosteroid administration “missing” as “no corticosteroid” and conducted a sensitivity analysis of patients with a yes/no entry.

We acknowledge the missing data in all our tables but did not perform any imputation for missing data. Given the nature of the study, the analysis team had concerns around interpretation, were the corticosteroid administration to be imputed. Accordingly, this was not done.

Bias

Data collected for ISARIC depended on the collection of COVID-19 test reports and staff resources, which may have varied between sites and at times of high clinical workload.

We explored potential selection bias by comparing our results to other reported studies.

Statistical analysis

We did not conduct statistical testing for the overall comparison of the two groups as this would have been subject to multiple testing and differences in invariably all variables would have been statistically significant with the large sample size.

We employed statistical disclosure control (SDC) measures to protect patient confidentiality and anonymity. Cells with N < 5 were either removed or replaced with NA or merged with other levels (NA could be 0 or suppressed). If small cells were ‘Missing’ or ‘Unknown’, these were not removed, as we assumed these occur at random by chance.

The multilevel multivariable regression model is described in the main text. We tested for all first-order interactions including the effect of time as week of admission (both linear and non-linear were tested).

The interrupted time series analysis used segmented linear regression to estimate the effect of the changepoints on the trends in corticosteroid administration[9]. Any corticosteroid was assigned 1 and no corticosteroid or missing corticosteroid 0. A quadratic linear model was fitted to the daily corticosteroid administration rates, which returned the temporal trend in the proportion of patients receiving corticosteroids with 95% confidence intervals (CIs), for before and after the RECOVERY trial publication and the initial guidelines.

Sensitivity analyses

- Complete case analysis excluding patients with corticosteroid missing (Table S9)
- Patients with a definite positive PCR test (Table S10)
- Excluding patients who died or received palliative discharge within two days of hospital admission (Table S11)

- Patients with a recorded clinical frailty score, which was included in the multivariable multilevel regression model (Table S12, Figure S2). Clinical frailty was missing from half the patients: from 53% of patients less than 50 years of age and 45% of patients 80 years or older. Hence this was conducted as a sensitivity analysis rather than included in the final model.

References

1 Horby PW. Low-cost dexamethasone reduces death by up to one third in hospitalised patients with severe respiratory complications of COVID-19 — RECOVERY Trial. https://www.recoverytrial.net/news/low-cost-dexamethasone-reduces-death-by-up-to-one-third-in-hospitalised-patients-with-severe-respiratory-complications-of-covid-19 (accessed Oct 10, 2020).

2 The WHO Rapid Evidence Appraisal for COVID-19 Therapies (REACT) Working Group. Association Between Administration of Systemic Corticosteroids and Mortality Among Critically Ill Patients With COVID-19: A Meta-analysis. *JAMA* 2020; published online Sept 2. DOI:10.1001/jama.2020.17023.

3 Närhi F, Drake T, Harrison E, *et al.* Delayed adoption of corticosteroids as standard of care for hypoxic patients with COVID-19 in the UK. .

4 NICE National Institute for Health and Care Excellence. COVID-19 rapid guideline: Managing COVID-19 v5.0. MAGICapp. 2021. https://app.magicapp.org/#/guideline/5083/section/67629 (accessed April 18, 2021).

5 Whitty C. Dexamethasone in the treatment of COVID-19: Implementation and management of supply for treatment in hospitals. 2020. https://www.cas.mhra.gov.uk/ViewandAcknowledgment/ViewAlert.aspx?AlertID=103054.

6 Knight SR, Ho A, Pius R, *et al.* Risk stratification of patients admitted to hospital with covid-19 using the ISARIC WHO Clinical Characterisation Protocol: Development and validation of the 4C Mortality Score. *BMJ* 2020; **370**: 22.

7 Rockwood K. Clinical Frailty Scale - Geriatric Medicine Research - Dalhousie University. https://www.dal.ca/sites/gmr/our-tools/clinical-frailty-scale.html (accessed June 13, 2021).

8 Rockwood K, Song X, MacKnight C, *et al.* A global clinical measure of fitness and frailty in elderly people. *CMAJ* 2005; **173**: 489–95.

9 Bernal JL, Cummins S, Gasparrini A. Interrupted time series regression for the evaluation of public health interventions: A tutorial. *Int J Epidemiol* 2017; **46**: 348–55.

Table S1. All adult patients admitted to acute care hospital between the 17th of June 2020 and the 14th of April 2021 (n=96 708). Corticosteroid is shortened to steroid here for space. Proportions read vertically.

label	Total N (% complete)	Missing N		Dexamethasone 6 mg daily	Any steroid	No steroid	Missing steroid
Total N (%)				45559 (47.1)	52138 (53.9)	34620 (35.8)	9950 (10.3)
PCR testing	83316 (86.2)	13392	PCR negative	410 (0.9)	487 (0.9)	334 (1.0)	15 (0.2)
			PCR positive	41171 (90.4)	47132 (90.4)	31131 (89.9)	3696 (37.1)
			Probable	10 (0.0)	14 (0.0)	10 (0.0)	2 (0.0)
			Not tested	266 (0.6)	310 (0.6)	180 (0.5)	5 (0.1)
			(Missing)	3702 (8.1)	4195 (8.0)	2965 (8.6)	6232 (62.6)
Age on admission (years)	96708 (100.0)	0	Median (IQR)	68.3 (55.7 to 79.6)	68.5 (55.7 to 79.8)	75.7 (57.9 to 85.2)	73.9 (59.0 to 84.1)
Age	96708 (100.0)	0	<50	7090 (15.6)	8183 (15.7)	6099 (17.6)	1359 (13.7)
			50-59	8167 (17.9)	9197 (17.6)	3341 (9.7)	1279 (12.9)
			60-69	9047 (19.9)	10195 (19.6)	4054 (11.7)	1573 (15.8)
			70-79	10192 (22.4)	11776 (22.6)	7235 (20.9)	2266 (22.8)
			80+	11063 (24.3)	12787 (24.5)	13891 (40.1)	3473 (34.9)
Sex	96581 (99.9)	127	Female	18653 (40.9)	21531 (41.3)	18030 (52.1)	4592 (46.2)
			Male	26840 (58.9)	30529 (58.6)	16557 (47.8)	5342 (53.7)
			(Missing)	66 (0.1)	78 (0.1)	33 (0.1)	16 (0.2)
Pregnant	10901 (11.3)	85807	No	3765 (8.3)	4322 (8.3)	3131 (9.0)	506 (5.1)
			Yes	97 (0.2)	157 (0.3)	1170 (3.4)	94 (0.9)
			Unknown/NA	641 (1.4)	745 (1.4)	564 (1.6)	212 (2.1)
			(Missing)	41056 (90.1)	46914 (90.0)	29755 (85.9)	9138 (91.8)
Ethnicity	83162 (86.0)	13546	White	32053 (70.4)	36765 (70.5)	26223 (75.7)	6507 (65.4)
			South Asian	3202 (7.0)	3616 (6.9)	1643 (4.7)	324 (3.3)
			East Asian	247 (0.5)	272 (0.5)	109 (0.3)	29 (0.3)
			Black	1045 (2.3)	1195 (2.3)	679 (2.0)	136 (1.4)
			Other ethnic minority	3030 (6.7)	3431 (6.6)	1740 (5.0)	493 (5.0)
			(Missing)	5982 (13.1)	6859 (13.2)	4226 (12.2)	2461 (24.7)
IMD quintile	96466 (99.7)	242	1 (least deprived)	5712 (12.5)	6513 (12.5)	4718 (13.6)	1044 (10.5)

			2	7827 (17.2)	8986 (17.2)	6176 (17.8)	1648 (16.6)
			3	9311 (20.4)	10638 (20.4)	7225 (20.9)	2768 (27.8)
			4	10505 (23.1)	11962 (22.9)	7262 (21.0)	2658 (26.7)
			5 (most deprived)	12072 (26.5)	13891 (26.6)	9169 (26.5)	1808 (18.2)
			(Missing)	132 (0.3)	148 (0.3)	70 (0.2)	24 (0.2)
**Severity of illness on admission**							
Oxygen saturation	90121 (93.2)	6587	Median (IQR)	93.0 (89.0 to 95.0)	93.0 (89.0 to 95.0)	96.0 (94.0 to 97.0)	94.0 (92.0 to 96.0)
Respiratory rate	85511 (88.4)	11197	Median (IQR)	22.0 (20.0 to 27.0)	22.0 (19.0 to 28.0)	18.0 (17.0 to 21.0)	20.0 (18.0 to 24.0)
C-reactive protein (mg/dl)	69346 (71.7)	27362	Median (IQR)	91.0 (47.0 to 154.0)	90.0 (46.0 to 154.0)	36.0 (11.0 to 87.0)	65.0 (24.3 to 128.0)
Blood Urea Nitrogen (mg/dl)	70527 (72.9)	26181	Median (IQR)	6.5 (4.7 to 9.7)	6.6 (4.7 to 9.8)	6.5 (4.4 to 10.0)	6.7 (4.7 to 10.3)
Glasgow Coma Scale	86064 (89.0)	10644	Median (IQR)	15.0 (15.0 to 15.0)	15.0 (15.0 to 15.0)	15.0 (15.0 to 15.0)	15.0 (15.0 to 15.0)
Glasgow Coma Scale	86064 (89.0)	10644	15	39953 (92.0)	45312 (91.4)	29271 (91.1)	3911 (89.6)
			<15	3460 (8.0)	4262 (8.6)	2855 (8.9)	453 (10.4)
Highest FiO2	83277 (86.1)	13431	Median (IQR)	0.28 (0.21 to 0.50)	0.28 (0.21 to 0.50)	0.21 (0.21 to 0.21)	0.21 (0.21 to 0.32)
Severity criteria	90578 (93.7)	6130	Mild COVID-19	10625 (23.3)	12753 (24.5)	24130 (69.7)	2910 (29.2)
			Moderate COVID-19	19712 (43.3)	21941 (42.1)	6464 (18.7)	1418 (14.3)
			Severe COVID-19	14821 (32.5)	16975 (32.6)	3054 (8.8)	933 (9.4)
			(Missing)	401 (0.9)	469 (0.9)	972 (2.8)	4689 (47.1)
**Comorbidities**							
Number of comorbidities	96708 (100.0)	0	0	10169 (22.3)	11372 (21.8)	7443 (21.5)	6638 (66.7)
			1	11409 (25.0)	12966 (24.9)	7385 (21.3)	1162 (11.7)
			2+	23981 (52.6)	27800 (53.3)	19792 (57.2)	2150 (21.6)
Any comorbidity	86286 (89.2)	10422	No	7236 (15.9)	8073 (15.5)	4632 (13.4)	616 (6.2)
			Yes	35928 (78.9)	41367 (79.3)	28159 (81.3)	3439 (34.6)
			(Missing)	2395 (5.3)	2698 (5.2)	1829 (5.3)	5895 (59.2)
Hypertension	82776 (85.6)	13932	No	22250 (48.8)	25481 (48.9)	16494 (47.6)	1862 (18.7)
			Yes	19596 (43.0)	22379 (42.9)	14869 (42.9)	1691 (17.0)

			(Missing)	3713 (8.1)	4278 (8.2)	3257 (9.4)	6397 (64.3)
Chronic cardiac disease	83200 (86.0)	13508	No	30819 (67.6)	35212 (67.5)	20923 (60.4)	2510 (25.2)
			Yes	11092 (24.3)	12768 (24.5)	10697 (30.9)	1090 (11.0)
			(Missing)	3648 (8.0)	4158 (8.0)	3000 (8.7)	6350 (63.8)
Non-asthmatic chronic pulmonary disease	83239 (86.1)	13469	No	34909 (76.6)	39721 (76.2)	26999 (78.0)	2959 (29.7)
			Yes	7022 (15.4)	8291 (15.9)	4619 (13.3)	650 (6.5)
			(Missing)	3628 (8.0)	4126 (7.9)	3002 (8.7)	6341 (63.7)
Asthma	83121 (86.0)	13587	No	35088 (77.0)	40088 (76.9)	27643 (79.8)	3035 (30.5)
			Yes	6822 (15.0)	7905 (15.2)	3910 (11.3)	540 (5.4)
			(Missing)	3649 (8.0)	4145 (8.0)	3067 (8.9)	6375 (64.1)
Diabetes	82181 (85.0)	14527	No diabetes	29056 (63.8)	33409 (64.1)	22814 (65.9)	2488 (25.0)
			Type 1 diabetes	881 (1.9)	1007 (1.9)	889 (2.6)	136 (1.4)
			Type 2 diabetes	11456 (25.1)	12980 (24.9)	7494 (21.6)	964 (9.7)
			(Missing)	4166 (9.1)	4742 (9.1)	3423 (9.9)	6362 (63.9)
Obesity	72920 (75.4)	23788	No	29975 (65.8)	34288 (65.8)	24578 (71.0)	2560 (25.7)
			Yes	7288 (16.0)	8226 (15.8)	2838 (8.2)	430 (4.3)
			(Missing)	8296 (18.2)	9624 (18.5)	7204 (20.8)	6960 (69.9)
Chronic neurological disease	82717 (85.5)	13991	No	37918 (83.2)	43304 (83.1)	27222 (78.6)	3167 (31.8)
			Yes	3801 (8.3)	4450 (8.5)	4183 (12.1)	391 (3.9)
			(Missing)	3840 (8.4)	4384 (8.4)	3215 (9.3)	6392 (64.2)
Dementia	82256 (85.1)	14452	No	38243 (83.9)	43689 (83.8)	26164 (75.6)	3104 (31.2)
			Yes	3356 (7.4)	3933 (7.5)	4934 (14.3)	432 (4.3)
			(Missing)	3960 (8.7)	4516 (8.7)	3522 (10.2)	6414 (64.5)
Chronic kidney disease	82992 (85.8)	13716	No	36027 (79.1)	41149 (78.9)	25558 (73.8)	2991 (30.1)
			Yes	5778 (12.7)	6700 (12.9)	5986 (17.3)	608 (6.1)
			(Missing)	3754 (8.2)	4289 (8.2)	3076 (8.9)	6351 (63.8)
Liver disease	82507 (85.3)	14201	No	40479 (88.8)	46299 (88.8)	30060 (86.8)	3409 (34.3)
			Yes	1134 (2.5)	1346 (2.6)	1258 (3.6)	135 (1.4)

			(Missing)	3946 (8.7)	4493 (8.6)	3302 (9.5)	6406 (64.4)
Moderato to severe liver disease	82389 (85.2)	14319	No	41031 (90.1)	46931 (90.0)	30490 (88.1)	3462 (34.8)
			Yes	528 (1.2)	649 (1.2)	778 (2.2)	79 (0.8)
			(Missing)	4000 (8.8)	4558 (8.7)	3352 (9.7)	6409 (64.4)
Mild liver disease	82126 (84.9)	14582	No	40832 (89.6)	46723 (89.6)	30627 (88.5)	3472 (34.9)
			Yes	622 (1.4)	720 (1.4)	523 (1.5)	61 (0.6)
			(Missing)	4105 (9.0)	4695 (9.0)	3470 (10.0)	6417 (64.5)
Malignancy	82708 (85.5)	14000	No	38396 (84.3)	43534 (83.5)	27876 (80.5)	3206 (32.2)
			Yes	3325 (7.3)	4226 (8.1)	3512 (10.1)	354 (3.6)
			(Missing)	3838 (8.4)	4378 (8.4)	3232 (9.3)	6390 (64.2)
Rheumatologic disease	82405 (85.2)	14303	No	36919 (81.0)	42135 (80.8)	26938 (77.8)	3123 (31.4)
			Yes	4661 (10.2)	5487 (10.5)	4338 (12.5)	384 (3.9)
			(Missing)	3979 (8.7)	4516 (8.7)	3344 (9.7)	6443 (64.8)
Pre-admission immunosuppressants, including corticosteroids	83122 (86.0)	13586	No	37620 (82.6)	42309 (81.1)	29547 (85.3)	2862 (28.8)
			Yes	4611 (10.1)	5967 (11.4)	2162 (6.2)	275 (2.8)
			(Missing)	3328 (7.3)	3862 (7.4)	2911 (8.4)	6813 (68.5)
AIDS/HIV	80938 (83.7)	15770	No	40670 (89.3)	46557 (89.3)	30635 (88.5)	3448 (34.7)
			Yes	138 (0.3)	170 (0.3)	119 (0.3)	9 (0.1)
			(Missing)	4751 (10.4)	5411 (10.4)	3866 (11.2)	6493 (65.3)
**Level of care and respiratory support**							
Critical care admission	91567 (94.7)	5141	No	35680 (78.3)	40614 (77.9)	32925 (95.1)	4585 (46.1)
			Yes	9656 (21.2)	11279 (21.6)	1496 (4.3)	668 (6.7)
			(Missing)	223 (0.5)	245 (0.5)	199 (0.6)	4697 (47.2)
Any oxygen	90751 (93.8)		No	2974 (6.5)	4173 (8.0)	21006 (60.7)	2047 (20.6)
			Yes	42442 (93.2)	47795 (91.7)	13251 (38.3)	2479 (24.9)
			(Missing)	143 (0.3)	170 (0.3)	363 (1.0)	5424 (54.5)
Highest level of respiratory support	90764 (93.9)	5944	No oxygen*	2902 (6.4)	4093 (7.9)	20939 (60.5)	2025 (20.4)
			Oxygen alone	26426 (58.0)	29589 (56.8)	11064 (32.0)	1721 (17.3)

			HFNC	2574 (5.6)	2860 (5.5)	709 (2.0)	224 (2.3)
			NIV	9548 (21.0)	10546 (20.2)	930 (2.7)	334 (3.4)
			IMV	3967 (8.7)	4882 (9.4)	617 (1.8)	231 (2.3)
			(Missing)	142 (0.3)	168 (0.3)	361 (1.0)	5415 (54.4)

*Total for the ‘No oxygen’ group does not match the Any oxygen “No”, as we did not assume everyone with non-invasive ventilation received supplementary oxygen.

Table S2. Adult patients admitted to acute care hospital between the 17th of June 2020 and the 14th of April 2021 and receiving oxygen. Corticosteroid is shortened to steroid here for space. Missing data is included in the table. Proportions read vertically.

	Total N (% complete)	Missing N		Dexamethasone 6 mg daily	Any steroid	No steroid	Missing steroid
**Total N (%)**				42442 (66.8)	47795 (75.2)	13251 (20.9)	2479 (3.9)
PCR testing	57466 (90.5)	6059	PCR negative	393 (0.9)	456 (1.0)	162 (1.2)	6 (0.2)
			PCR positive	38424 (90.5)	43287 (90.6)	11927 (90.0)	1265 (51.0)
			Probable	10 (0.0)	14 (0.0)	5 (0.0)	0 (0.0)
			Not tested	248 (0.6)	284 (0.6)	59 (0.4)	1 (0.0)
			(Missing)	3894 (9.2)	3754 (7.9)	1239 (9.4)	1207 (48.7)
Age on admission (years)	63525 (100.0)	0	Median (IQR)	68.0 (55.7 to 79.3)	68.1 (55.7 to 79.4)	77.8 (64.3 to 85.9)	70.5 (56.6 to 81.2)
Age	63525 (100.0)	0	<50	6584 (15.5)	7431 (15.5)	1418 (10.7)	358 (14.4)
			50-59	7729 (18.2)	8625 (18.0)	1244 (9.4)	408 (16.5)
			60-69	8602 (20.3)	9567 (20.0)	1652 (12.5)	449 (18.1)
			70-79	9517 (22.4)	10786 (22.6)	3088 (23.3)	592 (23.9)
			80+	10010 (23.6)	11386 (23.8)	5849 (44.1)	672 (27.1)
Sex	63435 (99.9)	90	Female	17285 (40.7)	19558 (40.9)	6483 (48.9)	1039 (41.9)
			Male	25095 (59.1)	28165 (58.9)	6755 (51.0)	1435 (57.9)
			(Missing)	62 (0.1)	72 (0.2)	13 (0.1)	5 (0.2)
Pregnant	6037 (9.5)	57488	No	3498 (8.2)	3931 (8.2)	772 (5.8)	165 (6.7)
			Yes	84 (0.2)	120 (0.3)	165 (1.2)	18 (0.7)
			Unknown/NA	591 (1.4)	666 (1.4)	153 (1.2)	47 (1.9)
			(Missing)	38269 (90.2)	43078 (90.1)	12161 (91.8)	2249 (90.7)
Ethnicity	54995 (86.6)	8530	White	29880 (70.4)	33663 (70.4)	10304 (77.8)	1551 (62.6)
			South Asian	2972 (7.0)	3325 (7.0)	518 (3.9)	61 (2.5)
			East Asian	229 (0.5)	248 (0.5)	41 (0.3)	11 (0.4)
			Black	982 (2.3)	1111 (2.3)	202 (1.5)	46 (1.9)
			Other ethnic minority	2834 (6.7)	3168 (6.6)	594 (4.5)	152 (6.1)
			(Missing)	5545 (13.1)	6280 (13.1)	1592 (12.0)	658 (26.5)
NHS region	63111 (99.3)	414	East of England	4101 (9.7)	4856 (10.2)	1775 (13.5)	170 (6.9)

			London	3260 (7.7)	3634 (7.7)	625 (4.7)	120 (4.9)
			Midlands	8986 (21.3)	10346 (21.8)	3104 (23.6)	311 (12.7)
			North East and Yorkshire	6774 (16.1)	7703 (16.2)	1883 (14.3)	186 (7.6)
			Northern Ireland	58 (0.1)	61 (0.1)	NA	NA
			North West	8172 (19.4)	9042 (19.0)	2477 (18.8)	366 (14.9)
			Scotland	946 (2.2)	1002 (2.1)	196 (1.5)	86 (3.5)
			South East	5336 (12.7)	5914 (12.5)	1826 (13.9)	946 (38.5)
			South West	3436 (8.1)	3679 (7.7)	940 (7.1)	255 (10.4)
			Wales	1095 (2.6)	1247 (2.6)	346 (2.6)	13 (0.5)
IMD quintile	63355 (99.7)	170	1 (least deprived)	5337 (12.6)	5969 (12.5)	1803 (13.6)	291 (11.7)
			2	7263 (17.1)	8199 (17.2)	2394 (18.1)	376 (15.2)
			3	8663 (20.4)	9722 (20.3)	2875 (21.7)	425 (17.1)
			4	9814 (23.1)	11018 (23.1)	2670 (20.1)	846 (34.1)
			5 (most deprived)	11238 (26.5)	12746 (26.7)	3486 (26.3)	535 (21.6)
			(Missing)	127 (0.3)	141 (0.3)	23 (0.2)	6 (0.2)
**Severity of illness on admission**							
Oxygen saturation	62726 (98.7)	799	Median (IQR)	92.0 (89.0 to 95.0)	92.0 (89.0 to 95.0)	94.0 (92.0 to 96.0)	93.0 (90.0 to 95.0)
Respiratory rate (breaths/min)	59739 (94.0)	3786	Median (IQR)	22.0 (20.0 to 28.0)	22.0 (20.0 to 28.0)	20.0 (18.0 to 24.0)	22.0 (19.0 to 26.0)
C-reactive protein (mg/dl)	50470 (79.4)	13055	Median (IQR)	93.0 (49.0 to 156.4)	93.0 (49.0 to 157.0)	52.2 (18.0 to 112.0)	87.0 (39.0 to 150.0)
Blood Urea Nitrogen (mg/dl)	50591 (79.6)	12934	Median (IQR)	6.5 (4.7 to 9.7)	6.6 (4.7 to 9.8)	7.3 (5.0 to 11.5)	6.8 (4.8 to 10.6)
Glasgow Coma Scale	60465 (95.2)	3060	Median (IQR)	15.0 (15.0 to 15.0)	15.0 (15.0 to 15.0)	15.0 (15.0 to 15.0)	15.0 (15.0 to 15.0)
Glasgow Coma Scale	60465 (95.2)	3060	15	37408 (92.0)	41738 (91.3)	10919 (87.6)	2006 (88.2)
			<15	3267 (8.0)	3986 (8.7)	1548 (12.4)	268 (11.8)
Highest FiO2 on day of admission	59026 (92.9)	4499	Median (IQR)	0.32 (0.24 to 0.50)	0.32 (0.24 to 0.50)	0.21 (0.21 to 0.28)	0.32 (0.24 to 0.50)
Severity criteria	62969 (99.1)	556	Mild COVID-19	8575 (20.2)	9757 (20.4)	6095 (46.0)	741 (29.9)
			Moderate COVID-19	19092 (45.0)	21165 (44.3)	4608 (34.8)	1003 (40.5)
			Severe COVID-19	14488 (34.1)	16548 (34.6)	2392 (18.1)	660 (26.6)

			(Missing)	287 (0.7)	325 (0.7)	156 (1.2)	75 (3.0)
**Comorbidities**							
Any comorbidity	59655 (93.9)	3870	No	6766 (15.9)	7446 (15.6)	1299 (9.8)	293 (11.8)
			Yes	33497 (78.9)	37930 (79.4)	11309 (85.3)	1378 (55.6)
			(Missing)	2179 (5.1)	2419 (5.1)	643 (4.9)	808 (32.6)
Hypertension	57493 (90.5)	6032	No	20755 (48.9)	23326 (48.8)	5878 (44.4)	821 (33.1)
			Yes	18304 (43.1)	20633 (43.2)	6155 (46.4)	680 (27.4)
			(Missing)	3383 (8.0)	3836 (8.0)	1218 (9.2)	978 (39.5)
Chronic cardiac disease	57800 (91.0)	5725	No	28850 (68.0)	32419 (67.8)	7595 (57.3)	1152 (46.5)
			Yes	10268 (24.2)	11650 (24.4)	4578 (34.5)	406 (16.4)
			(Missing)	3324 (7.8)	3726 (7.8)	1078 (8.1)	921 (37.2)
Non-asthmatic chronic pulmonary disease	57790 (91.0)	5735	No	32593 (76.8)	36506 (76.4)	9766 (73.7)	1288 (52.0)
			Yes	6540 (15.4)	7581 (15.9)	2388 (18.0)	261 (10.5)
			(Missing)	3309 (7.8)	3708 (7.8)	1097 (8.3)	930 (37.5)
Asthma	57731 (90.9)	5794	No	32747 (77.2)	36822 (77.0)	10518 (79.4)	1311 (52.9)
			Yes	6374 (15.0)	7256 (15.2)	1594 (12.0)	230 (9.3)
			(Missing)	3321 (7.8)	3717 (7.8)	1139 (8.6)	938 (37.8)
Diabetes	57039 (89.8)	6486	No diabetes	27117 (63.9)	30626 (64.1)	8519 (64.3)	1076 (43.4)
			Type 1 diabetes	797 (1.9)	894 (1.9)	314 (2.4)	53 (2.1)
			Type 2 diabetes	10737 (25.3)	12035 (25.2)	3131 (23.6)	391 (15.8)
			(Missing)	3791 (8.9)	4240 (8.9)	1287 (9.7)	959 (38.7)
Obesity	51023 (80.3)	12502	No	27885 (65.7)	31324 (65.5)	9267 (69.9)	1147 (46.3)
			Yes	7008 (16.5)	7846 (16.4)	1217 (9.2)	222 (9.0)
			(Missing)	7549 (17.8)	8625 (18.0)	2767 (20.9)	1110 (44.8)
Chronic neurological disease	57442 (90.4)	6083	No	35472 (83.6)	39856 (83.4)	10355 (78.1)	1400 (56.5)
			Yes	3460 (8.2)	3992 (8.4)	1696 (12.8)	143 (5.8)
			(Missing)	3510 (8.3)	3947 (8.3)	1200 (9.1)	936 (37.8)
Dementia	57210 (90.1)	6315	No	35858 (84.5)	40291 (84.3)	9862 (74.4)	1405 (56.7)
			Yes	2989 (7.0)	3472 (7.3)	2058 (15.5)	122 (4.9)

			(Missing)	3595 (8.5)	4032 (8.4)	1331 (10.0)	952 (38.4)
Chronic kidney disease	57615 (90.7)	5910	No	33688 (79.4)	37884 (79.3)	9613 (72.5)	1334 (53.8)
			Yes	5329 (12.6)	6059 (12.7)	2506 (18.9)	219 (8.8)
			(Missing)	3425 (8.1)	3852 (8.1)	1132 (8.5)	926 (37.4)
Moderate to severe liver disease	57231 (90.1)	6294	No	38312 (90.3)	43138 (90.3)	11669 (88.1)	1512 (61.0)
			Yes	490 (1.2)	573 (1.2)	315 (2.4)	24 (1.0)
			(Missing)	3640 (8.6)	4084 (8.5)	1267 (9.6)	943 (38.0)
Mild liver disease	57042 (89.8)	6483	No	38114 (89.8)	42917 (89.8)	11692 (88.2)	1507 (60.8)
			Yes	583 (1.4)	661 (1.4)	238 (1.8)	27 (1.1)
			(Missing)	3745 (8.8)	4217 (8.8)	1321 (10.0)	945 (38.1)
Malignancy	57443 (90.4)	6082	No	35921 (84.6)	40233 (84.2)	10523 (79.4)	1419 (57.2)
			Yes	3017 (7.1)	3627 (7.6)	1518 (11.5)	123 (5.0)
			(Missing)	3504 (8.3)	3935 (8.2)	1210 (9.1)	937 (37.8)
Rheumatologic disease	57281 (90.2)	6244	No	34489 (81.3)	38772 (81.1)	10210 (77.1)	1380 (55.7)
			Yes	4339 (10.2)	4982 (10.4)	1788 (13.5)	149 (6.0)
			(Missing)	3614 (8.5)	4041 (8.5)	1253 (9.5)	950 (38.3)
Pre-admission immunosuppressants, including corticosteroids	57864 (91.1)	5661	No	35105 (82.7)	39050 (81.7)	11056 (83.4)	1285 (51.8)
			Yes	4288 (10.1)	5264 (11.0)	1075 (8.1)	134 (5.4)
			(Missing)	3049 (7.2)	3481 (7.3)	1120 (8.5)	1060 (42.8)
AIDS/HIV	56194 (88.5)	7331	No	37991 (89.5)	42787 (89.5)	11697 (88.3)	1507 (60.8)
			Yes	127 (0.3)	155 (0.3)	45 (0.3)	3 (0.1)
			(Missing)	4324 (10.2)	4853 (10.2)	1509 (11.4)	969 (39.1)
**Level of care**							
Critical care admission	63187 (99.5)	338	No	32675 (77.0)	36415 (76.2)	11950 (90.2)	1913 (77.2)
			Yes	9585 (22.6)	11185 (23.4)	1224 (9.2)	500 (20.2)
			(Missing)	182 (0.4)	195 (0.4)	77 (0.6)	66 (2.7)
Invasive ventilation	62476 (98.3)	1049	No	37959 (89.4)	42346 (88.6)	12453 (94.0)	1947 (78.5)
			Yes	3967 (9.3)	4882 (10.2)	617 (4.7)	231 (9.3)

			(Missing)	516 (1.2)	567 (1.2)	181 (1.4)	301 (12.1)

Any steroid includes patients of whom 89% received dexamethasone 6 mg daily, 9% received dexamethasone other/unknown dose and/or frequency, 1% received hydrocortisone, 1% received prednisolone and 0.1% methylprednisolone.

Table S3. Adult patients admitted to acute care hospital between the 17th of June 2020 and the 14th of April 2021 and receiving oxygen by severity criteria. Missing data is included in the table. Proportions read vertically.

label	Total N (% complete)	Missing N	levels	Mild COVID-19	Moderate COVID-19	Severe COVID-19	Missing severity
Total N (%)				16593 (26.1)	26776 (42.2)	19600 (30.9)	556 (0.9)
PCR testing	57466 (90.5)	6059	PCR negative	147 (0.9)	290 (1.1)	184 (0.9)	3 (0.5)
			PCR positive	14803 (89.2)	23845 (89.1)	17367 (88.6)	464 (83.5)
			Probable	3 (0.0)	9 (0.0)	7 (0.0)	0 (0.0)
			Not tested	90 (0.5)	143 (0.5)	110 (0.6)	1 (0.2)
			(Missing)	1550 (9.3)	2489 (9.3)	1932 (9.9)	88 (15.8)
Age on admission (years)	63525 (100.0)	0	Median (IQR)	73.9 (58.8 to 84.2)	69.3 (56.1 to 80.9)	69.0 (56.8 to 79.4)	67.6 (54.7 to 79.0)
Sex	63435 (99.9)	90	Female	7703 (46.4)	11275 (42.1)	7850 (40.1)	252 (45.3)
			Male	8865 (53.4)	15466 (57.8)	11720 (59.8)	304 (54.7)
			(Missing)	25 (0.2)	35 (0.1)	30 (0.2)	0 (0.0)
Pregnant	6037 (9.5)	57488	No	1235 (7.4)	2165 (8.1)	1432 (7.3)	36 (6.5)
			Yes	136 (0.8)	85 (0.3)	70 (0.4)	12 (2.2)
			Unknown/NA	220 (1.3)	382 (1.4)	249 (1.3)	15 (2.7)
			(Missing)	15002 (90.4)	24144 (90.2)	17849 (91.1)	493 (88.7)
Ethnicity	54995 (86.6)	8530	White	12404 (74.8)	19161 (71.6)	13573 (69.2)	380 (68.3)
			South Asian	767 (4.6)	1658 (6.2)	1461 (7.5)	18 (3.2)
			East Asian	52 (0.3)	125 (0.5)	121 (0.6)	2 (0.4)
			Black	309 (1.9)	541 (2.0)	503 (2.6)	6 (1.1)
			Other ethnic minority	861 (5.2)	1692 (6.3)	1328 (6.8)	33 (5.9)
			(Missing)	2200 (13.3)	3599 (13.4)	2614 (13.3)	117 (21.0)
NHS region	63111 (99.3)	414	East of England	1961 (11.8)	2787 (10.4)	1978 (10.1)	75 (13.5)
			London	780 (4.7)	1630 (6.1)	1920 (9.8)	49 (8.8)
			Midlands	3774 (22.7)	6017 (22.5)	3871 (19.8)	99 (17.8)
			North East and Yorkshire	2592 (15.6)	4187 (15.6)	2945 (15.0)	48 (8.6)
			Northern Ireland	9 (0.1)	24 (0.1)	28 (0.1)	2 (0.4)
			North West	3175 (19.1)	5086 (19.0)	3575 (18.2)	49 (8.8)
			Scotland	277 (1.7)	525 (2.0)	389 (2.0)	93 (16.7)
			South East	2270 (13.7)	3670 (13.7)	2663 (13.6)	83 (14.9)

			South West	1319 (7.9)	1970 (7.4)	1537 (7.8)	48 (8.6)
			Wales	336 (2.0)	722 (2.7)	543 (2.8)	5 (0.9)
			(Missing)	100 (0.6)	158 (0.6)	151 (0.8)	5 (0.9)
IMD quintile	63355 (99.7)	170	1 (least deprived)	2363 (14.2)	3420 (12.8)	2237 (11.4)	43 (7.7)
			2	2892 (17.4)	4745 (17.7)	3272 (16.7)	60 (10.8)
			3	3374 (20.3)	5461 (20.4)	4031 (20.6)	156 (28.1)
			4	3747 (22.6)	6100 (22.8)	4545 (23.2)	142 (25.5)
			5 (most deprived)	4180 (25.2)	6977 (26.1)	5457 (27.8)	153 (27.5)
			(Missing)	37 (0.2)	73 (0.3)	58 (0.3)	2 (0.4)
Clinical frailty	31107 (49.0)	32418	1	646 (3.9)	1407 (5.3)	1055 (5.4)	11 (2.0)
			2	1077 (6.5)	2577 (9.6)	1939 (9.9)	18 (3.2)
			3	1308 (7.9)	2685 (10.0)	1985 (10.1)	7 (1.3)
			4	1085 (6.5)	1718 (6.4)	1397 (7.1)	9 (1.6)
			5	938 (5.7)	1396 (5.2)	989 (5.0)	9 (1.6)
			6	1301 (7.8)	1735 (6.5)	1241 (6.3)	8 (1.4)
			7	1005 (6.1)	1358 (5.1)	1030 (5.3)	3 (0.5)
			8	228 (1.4)	344 (1.3)	330 (1.7)	1 (0.2)
			9	65 (0.4)	99 (0.4)	102 (0.5)	1 (0.2)
			(Missing)	8940 (53.9)	13457 (50.3)	9532 (48.6)	489 (87.9)
**Severity of illness on admission**							
Oxygen saturation	62726 (98.7)	799	Median (IQR)	95.0 (94.0 to 96.0)	93.0 (92.0 to 94.0)	88.0 (83.0 to 89.0)	NA (NA to NA)
Glasgow coma scale (GCS)	60465 (95.2)	3060	Median (IQR)	15.0 (15.0 to 15.0)	15.0 (15.0 to 15.0)	15.0 (15.0 to 15.0)	15.0 (15.0 to 15.0)
Glasgow coma scale (GCS)	60465 (95.2)	3060	15	14334 (91.2)	23531 (91.4)	16634 (88.5)	164 (78.1)
			<15	1381 (8.8)	2224 (8.6)	2151 (11.5)	46 (21.9)
Blood Urea Nitrogen (urea) (mg/dL)	50591 (79.6)	12934	Median (IQR)	6.6 (4.7 to 10.0)	6.5 (4.6 to 9.7)	7.1 (5.0 to 10.9)	6.6 (4.5 to 9.7)
Blood urea nitrogen (mg/dL)	50591 (79.6)	12934	<7	6350 (38.3)	11944 (44.6)	7976 (40.7)	185 (33.3)
			<14	3933 (23.7)	7212 (26.9)	6159 (31.4)	102 (18.3)
			14+	1575 (9.5)	2565 (9.6)	2540 (13.0)	50 (9.0)
			(Missing)	4735 (28.5)	5055 (18.9)	2925 (14.9)	219 (39.4)
C-reactive protein (mg/dL)	50470 (79.4)	13055	Median (IQR)	60.0 (24.0 to 115.0)	82.0 (42.0 to 141.0)	112.0 (59.5 to 182.0)	76.0 (33.1 to 132.0)

C-reactive protein (mg/dL)	50470 (79.4)	13055	<50	5036 (30.4)	6590 (24.6)	3332 (17.0)	115 (20.7)
			50-99	2968 (17.9)	6283 (23.5)	4142 (21.1)	96 (17.3)
			100+	3488 (21.0)	8961 (33.5)	9335 (47.6)	124 (22.3)
			(Missing)	5101 (30.7)	4942 (18.5)	2791 (14.2)	221 (39.7)
Respiratory rate (breaths/min)	59739 (94.0)	3786	Median (IQR)	18.0 (17.0 to 20.0)	22.0 (20.0 to 25.0)	28.0 (22.0 to 35.0)	NA (NA to NA)
Highest FiO2 on day of admission	46496 (73.2)	17029	Median (IQR)	0.2 (0.2 to 0.3)	0.3 (0.2 to 0.5)	0.5 (0.3 to 0.7)	0.3 (0.2 to 0.5)
**Comorbidities**							
Number of comorbidities	63525 (100.0)	0	0	3864 (23.3)	5759 (21.5)	3710 (18.9)	302 (54.3)
			1	3780 (22.8)	6610 (24.7)	4721 (24.1)	81 (14.6)
			2+	8949 (53.9)	14407 (53.8)	11169 (57.0)	173 (31.1)
Any comorbidity	59655 (93.9)	3870	No	2224 (13.4)	4040 (15.1)	2720 (13.9)	54 (9.7)
			Yes	12926 (77.9)	21343 (79.7)	16082 (82.1)	266 (47.8)
			(Missing)	1443 (8.7)	1393 (5.2)	798 (4.1)	236 (42.4)
Hypertension	57493 (90.5)	6032	No	7433 (44.8)	13046 (48.7)	9395 (47.9)	151 (27.2)
			Yes	7079 (42.7)	11490 (42.9)	8752 (44.7)	147 (26.4)
			(Missing)	2081 (12.5)	2240 (8.4)	1453 (7.4)	258 (46.4)
Chronic cardiac disease	57800 (91.0)	5725	No	10044 (60.5)	17774 (66.4)	13134 (67.0)	214 (38.5)
			Yes	4583 (27.6)	6896 (25.8)	5071 (25.9)	84 (15.1)
			(Missing)	1966 (11.8)	2106 (7.9)	1395 (7.1)	258 (46.4)
Non-asthmatic chronic pulmonary disease	57790 (91.0)	5735	No	13010 (78.4)	20601 (76.9)	13698 (69.9)	251 (45.1)
			Yes	1586 (9.6)	4019 (15.0)	4580 (23.4)	45 (8.1)
			(Missing)	1997 (12.0)	2156 (8.1)	1322 (6.7)	260 (46.8)
Asthma	57731 (90.9)	5794	No	12655 (76.3)	20407 (76.2)	15335 (78.2)	254 (45.7)
			Yes	1964 (11.8)	4206 (15.7)	2866 (14.6)	44 (7.9)
			(Missing)	1974 (11.9)	2163 (8.1)	1399 (7.1)	258 (46.4)
Diabetes	57039 (89.8)	6486	No diabetes	10299 (62.1)	17193 (64.2)	12532 (63.9)	197 (35.4)
			Type 1 diabetes	377 (2.3)	544 (2.0)	331 (1.7)	9 (1.6)
			Type 2 diabetes	3740 (22.5)	6609 (24.7)	5122 (26.1)	86 (15.5)
			(Missing)	2177 (13.1)	2430 (9.1)	1615 (8.2)	264 (47.5)

Obesity	51023 (80.3)	12502	No	11194 (67.5)	17753 (66.3)	12603 (64.3)	188 (33.8)
			Yes	1650 (9.9)	4015 (15.0)	3548 (18.1)	72 (12.9)
			(Missing)	3749 (22.6)	5008 (18.7)	3449 (17.6)	296 (53.2)
Chronic neurological disease	57442 (90.4)	6083	No	12743 (76.8)	22119 (82.6)	16475 (84.1)	274 (49.3)
			Yes	1806 (10.9)	2352 (8.8)	1648 (8.4)	25 (4.5)
			(Missing)	2044 (12.3)	2305 (8.6)	1477 (7.5)	257 (46.2)
Dementia	57210 (90.1)	6315	No	12578 (75.8)	22153 (82.7)	16548 (84.4)	279 (50.2)
			Yes	1853 (11.2)	2255 (8.4)	1533 (7.8)	11 (2.0)
			(Missing)	2162 (13.0)	2368 (8.8)	1519 (7.8)	266 (47.8)
Chronic kidney disease	57615 (90.7)	5910	No	11981 (72.2)	20884 (78.0)	15711 (80.2)	255 (45.9)
			Yes	2605 (15.7)	3696 (13.8)	2438 (12.4)	45 (8.1)
			(Missing)	2007 (12.1)	2196 (8.2)	1451 (7.4)	256 (46.0)
Mild liver disease	57042 (89.8)	6483	No	14163 (85.4)	23975 (89.5)	17693 (90.3)	285 (51.3)
			Yes	273 (1.6)	338 (1.3)	307 (1.6)	8 (1.4)
			(Missing)	2157 (13.0)	2463 (9.2)	1600 (8.2)	263 (47.3)
Moderate to severe liver disease	57231 (90.1)	6294	No	14178 (85.4)	24030 (89.7)	17825 (90.9)	286 (51.4)
			Yes	313 (1.9)	357 (1.3)	234 (1.2)	8 (1.4)
			(Missing)	2102 (12.7)	2389 (8.9)	1541 (7.9)	262 (47.1)
Malignancy	57443 (90.4)	6082	No	12895 (77.7)	22366 (83.5)	16638 (84.9)	276 (49.6)
			Yes	1632 (9.8)	2131 (8.0)	1484 (7.6)	21 (3.8)
			(Missing)	2066 (12.5)	2279 (8.5)	1478 (7.5)	259 (46.6)
Rheumatologic disease	57281 (90.2)	6244	No	12586 (75.9)	21534 (80.4)	15993 (81.6)	249 (44.8)
			Yes	1915 (11.5)	2894 (10.8)	2066 (10.5)	44 (7.9)
			(Missing)	2092 (12.6)	2348 (8.8)	1541 (7.9)	263 (47.3)
Pre-admission immunosuppressants, including corticosteroids	57864 (91.1)	5661	No	13271 (80.0)	21877 (81.7)	15956 (81.4)	287 (51.6)
			Yes	1369 (8.3)	2768 (10.3)	2311 (11.8)	25 (4.5)
			(Missing)	1953 (11.8)	2131 (8.0)	1333 (6.8)	244 (43.9)
AIDS/HIV	56194 (88.5)	7331	No	14150 (85.3)	23923 (89.3)	17631 (90.0)	287 (51.6)
			Yes	61 (0.4)	71 (0.3)	70 (0.4)	1 (0.2)

			(Missing)	2382 (14.4)	2782 (10.4)	1899 (9.7)	268 (48.2)
**Treatments, level of care and respiratory support**							
Any steroid	61046 (96.1)	2479	No	6095 (36.7)	4608 (17.2)	2392 (12.2)	156 (28.1)
			Yes	9757 (58.8)	21165 (79.0)	16548 (84.4)	325 (58.5)
			(Missing)	741 (4.5)	1003 (3.7)	660 (3.4)	75 (13.5)
Level of respiratory support	63525 (100.0)		Oxygen alone	13535 (81.6)	18584 (69.4)	9902 (50.5)	353 (63.5)
			HFNC	659 (4.0)	1686 (6.3)	1426 (7.3)	22 (4.0)
			NIV	1536 (9.3)	4625 (17.3)	5391 (27.5)	76 (13.7)
			IMV	863 (5.2)	1881 (7.0)	2881 (14.7)	105 (18.9)
Critical care admission	63187 (99.5)	338	No	14570 (87.8)	22006 (82.2)	13334 (68.0)	368 (66.2)
			Yes	1898 (11.4)	4639 (17.3)	6195 (31.6)	177 (31.8)
			(Missing)	125 (0.8)	131 (0.5)	71 (0.4)	11 (2.0)
If No to critical care admission:	42110 (66.3)	21415	Not indicated	8923 (53.8)	13765 (51.4)	7505 (38.3)	206 (37.1)
			Not appropriate	3091 (18.6)	4677 (17.5)	3871 (19.8)	72 (12.9)
			(Missing)	4579 (27.6)	8334 (31.1)	8224 (42.0)	278 (50.0)
**Outcomes**							
Short length of stay with poor outcome	63525 (100.0)	0	Death or palliative discharge within 2 days of admission	114 (0.7)	325 (1.2)	824 (4.2)	16 (2.9)
			Longer LOS or survival	16479 (99.3)	26451 (98.8)	18776 (95.8)	540 (97.1)
Outcome	61217 (96.4)	2308	Discharged alive	10133 (61.1)	17425 (65.1)	10302 (52.6)	284 (51.1)
			Death	3953 (23.8)	6203 (23.2)	6541 (33.4)	137 (24.6)
			Palliative discharge	221 (1.3)	259 (1.0)	214 (1.1)	8 (1.4)
			Continuing hospitalisation	1022 (6.2)	1085 (4.1)	960 (4.9)	37 (6.7)
			Transfer to other facility	709 (4.3)	853 (3.2)	838 (4.3)	33 (5.9)
			(Missing)	555 (3.3)	951 (3.6)	745 (3.8)	57 (10.3)
Length of stay	60212 (94.8)	3313	Median (IQR)	11.0 (5.0 to 21.0)	9.0 (5.0 to 16.0)	9.0 (5.0 to 17.0)	10.0 (6.0 to 18.5)

Table S4. Adult patients admitted to acute care hospital between the 17th of June 2020 and the 14th of April 2021 with moderate to severe COVID-19 (n=46376). Corticosteroid is shortened to steroid here for space. Missing data is included in the table. Proportions read vertically.

	Total N (% complete)	Missing N		Dexamethasone 6 mg daily	Any steroid	No steroid	Missing steroid
**Total N (%)**				33580 (72.4)	37713 (81.3)	7000 (15.1)	1663 (3.6)
PCR testing	41955 (90.5)	4421	PCR negative	325 (1.0)	375 (1.0)	93 (1.3)	6 (0.4)
			PCR positive	30390 (90.5)	34158 (90.6)	6259 (89.4)	795 (47.8)
			Probable	10 (0.0)	12 (0.0)	4 (0.1)	0 (0.0)
			Not tested	187 (0.6)	216 (0.6)	36 (0.5)	1 (0.1)
			(Missing)	2668 (7.9)	2952 (7.8)	608 (8.7)	861 (51.8)
Age on admission (years)	46376 (100.0)	0	Median (IQR)	67.2 (55.3 to 78.4)	67.3 (55.4 to 78.5)	78.0 (66.0 to 86.0)	69.6 (56.4 to 79.7)
Age	46376 (100.0)	0	<50	5362 (16.0)	6006 (15.9)	619 (8.8)	242 (14.6)
			50-59	6295 (18.7)	7037 (18.7)	626 (8.9)	288 (17.3)
			60-69	7045 (21.0)	7807 (20.7)	922 (13.2)	312 (18.8)
			70-79	7573 (22.6)	8543 (22.7)	1733 (24.8)	414 (24.9)
			80+	7305 (21.8)	8320 (22.1)	3100 (44.3)	407 (24.5)
Sex	46311 (99.9)	65	Female	13425 (40.0)	15141 (40.1)	3317 (47.4)	667 (40.1)
			Male	20111 (59.9)	22518 (59.7)	3674 (52.5)	994 (59.8)
			(Missing)	44 (0.1)	54 (0.1)	9 (0.1)	2 (0.1)
Pregnant	4383 (9.5)	41993	No	2812 (8.4)	3159 (8.4)	328 (4.7)	110 (6.6)
			Yes	61 (0.2)	84 (0.2)	61 (0.9)	10 (0.6)
			Unknown/NA	468 (1.4)	525 (1.4)	75 (1.1)	31 (1.9)
			(Missing)	30239 (90.1)	33945 (90.0)	6536 (93.4)	1512 (90.9)
Ethnicity	40163 (86.6)	6213	White	23338 (69.5)	26261 (69.6)	5458 (78.0)	1015 (61.0)
			South Asian	2510 (7.5)	2793 (7.4)	280 (4.0)	46 (2.8)
			East Asian	193 (0.6)	209 (0.6)	28 (0.4)	9 (0.5)
			Black	811 (2.4)	912 (2.4)	100 (1.4)	32 (1.9)
			Other ethnic minority	2345 (7.0)	2606 (6.9)	298 (4.3)	116 (7.0)
			(Missing)	4383 (13.1)	4932 (13.1)	836 (11.9)	445 (26.8)
NHS region	46067 (99.3)	309	East of England	3164 (9.5)	3728 (9.9)	918 (13.2)	119 (7.2)

			London	2808 (8.4)	3126 (8.3)	331 (4.8)	93 (5.7)
			Midlands	6997 (21.0)	8021 (21.4)	1648 (23.7)	219 (13.3)
			North East and Yorkshire	5288 (15.9)	5997 (16.0)	984 (14.1)	151 (9.2)
			Northern Ireland	48 (0.1)	50 (0.1)	NA	NA
			North West	6476 (19.4)	7146 (19.1)	1269 (18.2)	246 (15.0)
			Scotland	717 (2.1)	759 (2.0)	97 (1.4)	58 (3.5)
			South East	4263 (12.8)	4735 (12.6)	1012 (14.5)	586 (35.7)
			South West	2676 (8.0)	2862 (7.6)	487 (7.0)	158 (9.6)
			Wales	925 (2.8)	1044 (2.8)	210 (3.0)	11 (0.7)
IMD quintile	46245 (99.7)	131	1 (least deprived)	4073 (12.1)	4525 (12.0)	942 (13.5)	190 (11.4)
			2	5751 (17.1)	6465 (17.1)	1297 (18.5)	255 (15.3)
			3	6828 (20.3)	7655 (20.3)	1554 (22.2)	283 (17.0)
			4	7758 (23.1)	8719 (23.1)	1378 (19.7)	548 (33.0)
			5 (most deprived)	9070 (27.0)	10239 (27.1)	1813 (25.9)	382 (23.0)
			(Missing)	100 (0.3)	110 (0.3)	16 (0.2)	5 (0.3)
**Severity of illness at admission**							
Oxygen saturation	46217 (99.7)	159	Median (IQR)	91.0 (88.0 to 93.0)	91.0 (88.0 to 93.0)	92.0 (89.0 to 94.0)	91.0 (88.0 to 93.0)
Respiratory rate on admission	44608 (96.2)	1768	Median (IQR)	24.0 (21.0 to 28.0)	24.0 (21.0 to 28.0)	23.0 (20.0 to 28.0)	24.0 (20.0 to 28.0)
C-reactive protein (mg/dl)	38643 (83.3)	7733	Median (IQR)	99.0 (53.0 to 163.0)	99.0 (53.0 to 164.0)	63.0 (24.3 to 127.0)	96.0 (47.0 to 163.0)
Blood Urea Nitrogen (mg/dl)	38396 (82.8)	7980	Median (IQR)	6.6 (4.7 to 9.8)	6.6 (4.8 to 9.9)	7.7 (5.2 to 12.1)	7.0 (4.9 to 10.6)
Glasgow Coma Scale	44540 (96.0)	1836	Median (IQR)	15.0 (15.0 to 15.0)	15.0 (15.0 to 15.0)	15.0 (15.0 to 15.0)	15.0 (15.0 to 15.0)
Glasgow Coma Scale	44540 (96.0)	1836	15	29771 (91.9)	33136 (91.2)	5643 (84.9)	1386 (88.8)
			<15	2634 (8.1)	3198 (8.8)	1003 (15.1)	174 (11.2)
Highest FiO2 on day of admission	43696 (94.2)	2680	Median (IQR)	0.32 (0.24 to 0.60)	0.32 (0.24 to 0.60)	0.24 (0.21 to 0.32)	0.32 (0.24 to 0.60)
**Comorbidities**							
Any comorbidity	44185 (95.3)	2191	No	5442 (16.2)	5957 (15.8)	587 (8.4)	216 (13.0)
			Yes	26777 (79.7)	30253 (80.2)	6187 (88.4)	985 (59.2)

			(Missing)	1361 (4.1)	1503 (4.0)	226 (3.2)	462 (27.8)
Hypertension	42683 (92.0)	3693	No	16738 (49.8)	18733 (49.7)	3106 (44.4)	602 (36.2)
			Yes	14528 (43.3)	16382 (43.4)	3371 (48.2)	489 (29.4)
			(Missing)	2314 (6.9)	2598 (6.9)	523 (7.5)	572 (34.4)
Chronic cardiac disease	42875 (92.5)	3501	No	23290 (69.4)	26096 (69.2)	3962 (56.6)	850 (51.1)
			Yes	8037 (23.9)	9105 (24.1)	2578 (36.8)	284 (17.1)
			(Missing)	2253 (6.7)	2512 (6.7)	460 (6.6)	529 (31.8)
Non-asthmatic chronic pulmonary disease	42898 (92.5)	3478	No	25599 (76.2)	28562 (75.7)	4817 (68.8)	920 (55.3)
			Yes	5751 (17.1)	6668 (17.7)	1718 (24.5)	213 (12.8)
			(Missing)	2230 (6.6)	2483 (6.6)	465 (6.6)	530 (31.9)
Asthma	42814 (92.3)	3562	No	26083 (77.7)	29245 (77.5)	5548 (79.3)	949 (57.1)
			Yes	5235 (15.6)	5953 (15.8)	947 (13.5)	172 (10.3)
			(Missing)	2262 (6.7)	2515 (6.7)	505 (7.2)	542 (32.6)
Diabetes	42331 (91.3)	4045	No diabetes	21701 (64.6)	24445 (64.8)	4501 (64.3)	779 (46.8)
			Type 1 diabetes	616 (1.8)	693 (1.8)	145 (2.1)	37 (2.2)
			Type 2 diabetes	8628 (25.7)	9645 (25.6)	1798 (25.7)	288 (17.3)
			(Missing)	2635 (7.8)	2930 (7.8)	556 (7.9)	559 (33.6)
Obesity	37919 (81.8)	8457	No	22014 (65.6)	24651 (65.4)	4864 (69.5)	841 (50.6)
			Yes	5963 (17.8)	6674 (17.7)	726 (10.4)	163 (9.8)
			(Missing)	5603 (16.7)	6388 (16.9)	1410 (20.1)	659 (39.6)
Chronic neurological disease	42594 (91.8)	3782	No	28578 (85.1)	32028 (84.9)	5542 (79.2)	1024 (61.6)
			Yes	2592 (7.7)	2985 (7.9)	919 (13.1)	96 (5.8)
			(Missing)	2410 (7.2)	2700 (7.2)	539 (7.7)	543 (32.7)
Dementia	42489 (91.6)	3887	No	28918 (86.1)	32399 (85.9)	5271 (75.3)	1031 (62.0)
			Yes	2185 (6.5)	2559 (6.8)	1144 (16.3)	85 (5.1)
			(Missing)	2477 (7.4)	2755 (7.3)	585 (8.4)	547 (32.9)
Chronic kidney disease	42729 (92.1)	3647	No	27156 (80.9)	30470 (80.8)	5143 (73.5)	982 (59.0)
			Yes	4073 (12.1)	4617 (12.2)	1366 (19.5)	151 (9.1)

			(Missing)	2351 (7.0)	2626 (7.0)	491 (7.0)	530 (31.9)
Mild liver disease	42313 (91.2)	4063	No	30506 (90.8)	34267 (90.9)	6300 (90.0)	1101 (66.2)
			Yes	465 (1.4)	522 (1.4)	105 (1.5)	18 (1.1)
			(Missing)	2609 (7.8)	2924 (7.8)	595 (8.5)	544 (32.7)
Moderate or severe liver disease	42446 (91.5)	3930	No	30695 (91.4)	34474 (91.4)	6276 (89.7)	1105 (66.4)
			Yes	359 (1.1)	421 (1.1)	154 (2.2)	16 (1.0)
			(Missing)	2526 (7.5)	2818 (7.5)	570 (8.1)	542 (32.6)
Malignancy	42619 (91.9)	3757	No	28839 (85.9)	32274 (85.6)	5683 (81.2)	1047 (63.0)
			Yes	2328 (6.9)	2742 (7.3)	795 (11.4)	78 (4.7)
			(Missing)	2413 (7.2)	2697 (7.2)	522 (7.5)	538 (32.4)
Rheumatologic disease	42487 (91.6)	3889	No	27700 (82.5)	31062 (82.4)	5465 (78.1)	1000 (60.1)
			Yes	3377 (10.1)	3869 (10.3)	974 (13.9)	117 (7.0)
			(Missing)	2503 (7.5)	2782 (7.4)	561 (8.0)	546 (32.8)
Pre-admission immunosuppressants, including corticosteroids	42912 (92.5)	3464	No	27964 (83.3)	31073 (82.4)	5826 (83.2)	934 (56.2)
			Yes	3567 (10.6)	4299 (11.4)	678 (9.7)	102 (6.1)
			(Missing)	2049 (6.1)	2341 (6.2)	496 (7.1)	627 (37.7)
AIDS/HIV	41695 (89.9)	4681	No	30400 (90.5)	34156 (90.6)	6299 (90.0)	1099 (66.1)
			Yes	96 (0.3)	117 (0.3)	21 (0.3)	3 (0.2)
			(Missing)	3084 (9.2)	3440 (9.1)	680 (9.7)	561 (33.7)
**Level of care**							
Level of respiratory support	46376 (100.0)	0	No oxygen	0 (0.0)	0 (0.0)	0 (0.0)	0 (0.0)
			Oxygen alone	19646 (58.5)	21890 (58.0)	5498 (78.5)	1098 (66.0)
			HFNC	2198 (6.5)	2444 (6.5)	506 (7.2)	162 (9.7)
			NIV	8263 (24.6)	9146 (24.3)	632 (9.0)	238 (14.3)
			IMV	3473 (10.3)	4233 (11.2)	364 (5.2)	165 (9.9)
Critical care admission	46174 (99.6)	202	No	25093 (74.7)	27868 (73.9)	6223 (88.9)	1249 (75.1)
			Yes	8360 (24.9)	9709 (25.7)	748 (10.7)	377 (22.7)
			(Missing)	127 (0.4)	136 (0.4)	29 (0.4)	37 (2.2)

If No to critical care admission:	29818 (64.3)	16558	Not indicated		17734 (47.0)	3445 (49.2)	91 (5.5)
			Not appropriate		6903 (18.3)	1622 (23.2)	23 (1.4)
			(Missing)		13076 (34.7)	1933 (27.6)	1549 (93.1)

Any steroid includes patients of whom 89% received dexamethasone 6 mg daily, 9% received dexamethasone other/unknown dose and/or frequency, 1% received hydrocortisone, 1% received prednisolone and 0.1% methylprednisolone.

Table S5. Subgroup analysis of adult patients aged 80 or over admitted to acute care hospital between the 17th of June 2020 and the 14th of April 2021 with moderate to severe COVID-19. Corticosteroid is shortened to steroid here for space. Missing data is included in the table. Proportions read vertically.

	Total N (% complete)	Missing N		Any steroid*	No steroid	Missing steroid
Total N (%)				8320 (70.3)	3100 (26.2)	407 (3.4)
PCR testing	10637 (89.9)	1190	PCR negative	67 (0.8)	32 (1.0)	0 (0.0)
			PCR positive	7546 (90.7)	2806 (90.5)	233 (57.2)
			Probable	3 (0.0)	1 (0.0)	0 (0.0)
			Not tested	35 (0.4)	17 (0.5)	1 (0.2)
			(Missing)	669 (8.0)	244 (7.9)	173 (42.5)
Age on admission (years)	11827 (100.0)	0	Median (IQR)	85.7 (82.8 to 89.5)	86.9 (83.5 to 90.8)	86.4 (83.6 to 90.3)
Sex	11810 (99.9)	17	Female	3865 (46.5)	1623 (52.4)	211 (51.8)
			Male	4442 (53.4)	1473 (47.5)	196 (48.2)
			(Missing)	13 (0.2)	4 (0.1)	0 (0.0)
Ethnicity	10519 (88.9)	1308	White	6666 (80.1)	2607 (84.1)	283 (69.5)
			South Asian	328 (3.9)	59 (1.9)	2 (0.5)
			East Asian	26 (0.3)	4 (0.1)	1 (0.2)
			Black	97 (1.2)	24 (0.8)	2 (0.5)
			Other ethnic minority	323 (3.9)	85 (2.7)	12 (2.9)
			(Missing)	880 (10.6)	321 (10.4)	107 (26.3)
NHS region	11827 (100.0)	0	East of England	1026 (12.3)	436 (14.1)	28 (6.9)
			London	416 (5.0)	96 (3.1)	6 (1.5)
			Midlands	1900 (22.8)	734 (23.7)	58 (14.3)
			North East and Yorkshire	1379 (16.6)	491 (15.8)	31 (7.6)
			Northern Ireland	NA	NA	NA
			North West	1462 (17.6)	490 (15.8)	52 (12.8)
			Scotland	126 (1.5)	34 (1.1)	8 (2.0)
			South East	1071 (12.9)	508 (16.4)	177 (43.5)
			South West	649 (7.8)	211 (6.8)	45 (11.1)
			Wales	227 (2.7)	82 (2.6)	1 (0.2)
			(Missing)	61 (0.7)	18 (0.6)	1 (0.2)
IMD quintile	11805 (99.8)	22	1 (least deprived)	1304 (15.7)	525 (16.9)	59 (14.5)
			2	1601 (19.2)	658 (21.2)	68 (16.7)
			3	1839 (22.1)	716 (23.1)	66 (16.2)
			4	1779 (21.4)	561 (18.1)	144 (35.4)
			5 (most deprived)	1780 (21.4)	635 (20.5)	70 (17.2)

			(Missing)	17 (0.2)	5 (0.2)	0 (0.0)
Clinical frailty	6558 (55.4)	5269	1	46 (0.6)	7 (0.2)	3 (0.7)
			2	118 (1.4)	19 (0.6)	3 (0.7)
			3	480 (5.8)	87 (2.8)	7 (1.7)
			4	727 (8.7)	213 (6.9)	25 (6.1)
			5	889 (10.7)	278 (9.0)	23 (5.7)
			6	1193 (14.3)	475 (15.3)	27 (6.6)
			7	943 (11.3)	455 (14.7)	32 (7.9)
			8	254 (3.1)	157 (5.1)	6 (1.5)
			9	53 (0.6)	34 (1.1)	4 (1.0)
			(Missing)	3617 (43.5)	1375 (44.4)	277 (68.1)
**Severity of illness at admission**						
Oxygen saturation	11809 (99.8)	18	Median (IQR)	92.0 (88.0 to 94.0)	92.0 (89.0 to 94.0)	92.0 (88.0 to 93.0)
Respiratory rate on admission	11409 (96.5)	418	Median (IQR)	24.0 (20.0 to 28.0)	22.0 (20.0 to 27.0)	24.0 (20.0 to 27.0)
C-reactive protein (mg/dl)	9346 (79.0)	2481	Median (IQR)	89.0 (45.0 to 152.0)	60.2 (24.0 to 123.0)	77.0 (35.0 to 135.9)
Blood Urea Nitrogen (mg/dl)	9408 (79.5)	2419	Median (IQR)	9.6 (6.9 to 13.9)	9.5 (6.7 to 14.8)	9.4 (6.6 to 14.3)
Glasgow Coma Scale	11356 (96.0)	471	Median (IQR)	15.0 (15.0 to 15.0)	15.0 (15.0 to 15.0)	15.0 (15.0 to 15.0)
Glasgow Coma Scale	11356 (96.0)	471	15	6722 (84.1)	2409 (81.1)	322 (83.0)
			<15	1275 (15.9)	562 (18.9)	66 (17.0)
Highest FiO2 on day of admission	10984 (92.9)	843	Median (IQR)	0.28 (0.24 to 0.40)	0.24 (0.21 to 0.32)	0.32 (0.24 to 0.40)
Severity criteria	11827 (100.0)	0	Mild COVID-19	0 (0.0)	0 (0.0)	0 (0.0)
			Moderate COVID-19	4841 (58.2)	2076 (67.0)	265 (65.1)
			Severe COVID-19	3479 (41.8)	1024 (33.0)	142 (34.9)
**Comorbidities**						
Number of comorbidities	11827 (100.0)	0	0	644 (7.7)	181 (5.8)	158 (38.8)
			1	1360 (16.3)	435 (14.0)	53 (13.0)
			2+	6316 (75.9)	2484 (80.1)	196 (48.2)
Any comorbidity	11322 (95.7)	505	No	343 (4.1)	92 (3.0)	19 (4.7)
			Yes	7697 (92.5)	2921 (94.2)	250 (61.4)
			(Missing)	280 (3.4)	87 (2.8)	138 (33.9)
Hypertension	10902 (92.2)	925	No	2920 (35.1)	1132 (36.5)	108 (26.5)
			Yes	4848 (58.3)	1762 (56.8)	132 (32.4)
			(Missing)	552 (6.6)	206 (6.6)	167 (41.0)
Chronic cardiac disease	10976 (92.8)	851	No	3903 (46.9)	1360 (43.9)	145 (35.6)

			Yes	3908 (47.0)	1551 (50.0)	109 (26.8)
			(Missing)	509 (6.1)	189 (6.1)	153 (37.6)
Non-asthmatic chronic pulmonary disease	10956 (92.6)	871	No	5704 (68.6)	2143 (69.1)	184 (45.2)
			Yes	2095 (25.2)	762 (24.6)	68 (16.7)
			(Missing)	521 (6.3)	195 (6.3)	155 (38.1)
Asthma	10881 (92.0)	946	No	6755 (81.2)	2540 (81.9)	217 (53.3)
			Yes	1000 (12.0)	337 (10.9)	32 (7.9)
			(Missing)	565 (6.8)	223 (7.2)	158 (38.8)
Diabetes	10806 (91.4)	1021	No diabetes	5362 (64.4)	2080 (67.1)	182 (44.7)
			Type 1 diabetes	133 (1.6)	41 (1.3)	7 (1.7)
			Type 2 diabetes	2183 (26.2)	756 (24.4)	62 (15.2)
			(Missing)	642 (7.7)	223 (7.2)	156 (38.3)
Obesity	9310 (78.7)	2517	No	6215 (74.7)	2286 (73.7)	198 (48.6)
			Yes	458 (5.5)	139 (4.5)	14 (3.4)
			(Missing)	1647 (19.8)	675 (21.8)	195 (47.9)
Chronic neurological disease	10826 (91.5)	1001	No	6744 (81.1)	2431 (78.4)	218 (53.6)
			Yes	961 (11.6)	440 (14.2)	32 (7.9)
			(Missing)	615 (7.4)	229 (7.4)	157 (38.6)
Dementia	10716 (90.6)	1111	No	5878 (70.6)	1971 (63.6)	186 (45.7)
			Yes	1755 (21.1)	866 (27.9)	60 (14.7)
			(Missing)	687 (8.3)	263 (8.5)	161 (39.6)
Chronic kidney disease	10922 (92.3)	905	No	5495 (66.0)	2042 (65.9)	180 (44.2)
			Yes	2281 (27.4)	852 (27.5)	72 (17.7)
			(Missing)	544 (6.5)	206 (6.6)	155 (38.1)
Mild liver disease	10765 (91.0)	1062	No	7603 (91.4)	2821 (91.0)	248 (60.9)
			Yes	66 (0.8)	27 (0.9)	0 (0.0)
			(Missing)	651 (7.8)	252 (8.1)	159 (39.1)
Moderate liver disease	10821 (91.5)	1006	No	7647 (91.9)	2828 (91.2)	244 (60.0)
			Yes	62 (0.7)	36 (1.2)	4 (1.0)
			(Missing)	611 (7.3)	236 (7.6)	159 (39.1)
Malignancy	10853 (91.8)	974	No	6716 (80.7)	2454 (79.2)	216 (53.1)
			Yes	1017 (12.2)	414 (13.4)	36 (8.8)
			(Missing)	587 (7.1)	232 (7.5)	155 (38.1)
Rheumatologic disease	10796 (91.3)	1031	No	6391 (76.8)	2299 (74.2)	206 (50.6)

			Yes	1295 (15.6)	561 (18.1)	44 (10.8)
			(Missing)	634 (7.6)	240 (7.7)	157 (38.6)
Pre-admission immunosuppressants, including corticosteroids	10913 (92.3)	914	No	6867 (82.5)	2680 (86.5)	203 (49.9)
			Yes	906 (10.9)	228 (7.4)	29 (7.1)
			(Missing)	547 (6.6)	192 (6.2)	175 (43.0)
AIDS/HIV	10668 (90.2)	1159	No	7589 (91.2)	2811 (90.7)	247 (60.7)
			Yes	14 (0.2)	7 (0.2)	0 (0.0)
			(Missing)	717 (8.6)	282 (9.1)	160 (39.3)
**Level of care**						
Highest level of respiratory support	11827 (100.0)		Oxygen alone	6381 (76.7)	2669 (86.1)	337 (82.8)
			High flow nasal cannula	555 (6.7)	218 (7.0)	43 (10.6)
			Non-invasive ventilation	1320 (15.9)	201 (6.5)	25 (6.1)
			Invasive mechanical ventilation	64 (0.8)	12 (0.4)	2 (0.5)
Critical care admission	11766 (99.5)	61	No	7823 (94.0)	3024 (97.5)	385 (94.6)
			Yes	456 (5.5)	65 (2.1)	13 (3.2)
			(Missing)	41 (0.5)	11 (0.4)	9 (2.2)
If No to critical care admission	9484 (80.2)	2343	Not indicated	3630 (43.6)	1514 (48.8)	32 (7.9)
			Not appropriate	3310 (39.8)	985 (31.8)	13 (3.2)
			(Missing)	1380 (16.6)	601 (19.4)	362 (88.9)
**Patients with short length of stay and poor outcome**						
Short length of stay with poor outcome	11827 (100.0)	0	Death or palliative discharge within 2 days of admission	407 (4.9)	175 (5.6)	18 (4.4)
			Longer length of stay or survival	7913 (95.1)	2925 (94.4)	389 (95.6)

*88% dexamethasone 6 mg daily, 9% dexamethasone other/unknown dose and/or frequency, 1% hydrocortisone, 1% prednisolone and 0% (1/8320) methylprednisolone

Table S6. Subgroup analysis: characteristics of women aged 12-55 years who received any supplementary oxygen, admitted between the 17th of June 2020 and the 14th of April 2021, by pregnancy status.

label	Total N	Missing N	levels	Not pregnant	Pregnant	Unknown/NA
Total N (%)				4868 (80.6)	303 (5.0)	866 (14.3)
PCR testing	5401 (89.5)	636	Not tested	23 (0.5)	4 (1.3)	10 (1.2)
			PCR negative	63 (1.3)	3 (1.0)	9 (1.0)
			PCR positive	4309 (88.5)	255 (84.2)	723 (83.5)
			Probable	2 (0.0)	0 (0.0)	0 (0.0)
			(Missing)	471 (9.7)	41 (13.5)	124 (14.3)
Age on admission (years)	6037 (100.0)	0	Median (IQR)	48.2 (40.2 to 52.7)	31.3 (28.1 to 35.3)	46.2 (37.9 to 51.7)
Asymptomatic/ Symptomatic	6037 (100.0)	0	Symptomatic	4384 (90.1)	238 (78.5)	755 (87.2)
			Unknown	105 (2.2)	10 (3.3)	21 (2.4)
			Asymptomatic	379 (7.8)	55 (18.2)	90 (10.4)
Oxygen saturation	5925 (98.1)	112	Median (IQR)	93.0 (90.0 to 95.0)	96.0 (94.0 to 97.0)	94.0 (90.0 to 96.0)
Respiratory rate on admission	5644 (93.5)	393	Median (IQR)	22.0 (19.0 to 28.0)	20.0 (18.0 to 28.0)	22.0 (20.0 to 27.0)
C-reactive protein	4980 (82.5)	1057	Median (IQR)	77.8 (36.0 to 136.7)	65.0 (28.5 to 95.5)	72.0 (34.0 to 138.0)
Blood Urea Nitrogen (mg/dl)	4899 (81.1)	1138	Median (IQR)	4.1 (3.2 to 5.6)	2.1 (1.6 to 2.8)	4.0 (3.0 to 5.1)
Glasgow Coma Scale	5760 (95.4)	277	Median (IQR)	15.0 (15.0 to 15.0)	15.0 (15.0 to 15.0)	15.0 (15.0 to 15.0)
Glasgow Coma Scale	5760 (95.4)	277	15	4412 (94.3)	279 (97.9)	755 (95.0)
			<15	268 (5.7)	6 (2.1)	40 (5.0)
Highest FiO2	5661 (93.8)	376	Median (IQR)	0.28 (0.24 to 0.50)	0.21 (0.21 to 0.32)	0.28 (0.21) to 0.40)
Severity criteria	5974 (99.0)	63	Mild COVID-19	1235 (25.4)	136 (44.9)	220 (25.4)
			Moderate COVID-19	2165 (44.5)	85 (28.1)	382 (44.1)
			Severe COVID-19	1432 (29.4)	70 (23.1)	249 (28.8)
			(Missing)	36 (0.7)	12 (4.0)	15 (1.7)
Critical care admission	6013 (99.6)	24	No	3480 (71.5)	204 (67.3)	627 (72.4)
			Yes	1380 (28.3)	97 (32.0)	225 (26.0)
			(Missing)	8 (0.2)	2 (0.7)	14 (1.6)
Invasive ventilation	5940 (98.4)	97	No	4241 (87.1)	260 (85.8)	707 (81.6)
			Yes	582 (12.0)	40 (13.2)	110 (12.7)
			(Missing)	45 (0.9)	3 (1.0)	49 (5.7)
Level of respiratory support	6037 (100.0)	0	No oxygen	0 (0.0)	0 (0.0)	0 (0.0)
			Oxygen alone	3002 (61.7)	212 (70.0)	547 (63.2)
			HFNC	331 (6.8)	19 (6.3)	51 (5.9)
			NIV	953 (19.6)	32 (10.6)	158 (18.2)
			IMV	582 (12.0)	40 (13.2)	110 (12.7)

Table S7. Subgroup analysis: characteristics of women aged 12-55 years with moderate to severe COVID-19, admitted between the 17th of June 2020 and the 14th of April 2021, by pregnancy status.

label	Total N	Missing N	levels	Not pregnant	Pregnant*	Unknown/NA
Total N (%)				3597 (82.1)	155 (3.5)	631 (14.4)
PCR testing	5401 (89.5)	636	Not tested	18 (0.5)	NA	9 (1.4)
			PCR negative	45 (1.3)	NA	7 (1.1)
			PCR positive	3183 (88.5)	125 (80.6)	524 (83.0)
			Probable	2 (0.1)	NA	NA
			(Missing)	349 (9.7)	28 (18.1)	91 (14.4)
Age on admission (years)	6037 (100.0)	0	Median (IQR)	48.4 (41.0 to 52.7)	32.0 (28.8 to 35.8)	46.9 (39.0 to 51.9)
Asymptomatic/ symptomatic	6037 (100.0)	0	Symptomatic	3413 (94.9)	147 (94.8)	576 (91.3)
			Unknown	44 (1.2)	2 (1.3)	11 (1.7)
			Asymptomatic	140 (3.9)	6 (3.9)	44 (7.0)
Oxygen saturation	5925 (98.1)	112	Median (IQR)	92.0 (89.0 to 94.0)	94.0 (92.0 to 96.0)	92.0 (89.0 to 94.0)
Respiratory rate on admission	5644 (93.5)	393	Median (IQR)	24.0 (21.0 to 29.0)	28.0 (23.8 to 34.2)	24.0 (22.0 to 30.0)
C-reactive protein	3779 (86.2)	604	Median (IQR)	83.0 (43.8 to 143.0)	75.8 (49.0 to 105.0)	81.0 (41.6 to 150.0)
Blood Urea Nitrogen (mg/dl)	3682 (84.0)	701	Median (IQR)	4.1 (3.2 to 5.6)	2.0 (1.5 to 2.5)	4.0 (3.0 to 5.0)
Glasgow Coma Scale	4211 (96.1)	172	Median (IQR)	15.0 (15.0 to 15.0)	15.0 (15.0 to 15.0)	15.0 (15.0 to 15.0)
Glasgow Coma Scale	4211 (96.1)	172	15	3274 (94.2)	143 (96.6)	554 (94.5)
			<15	203 (5.8)	5 (3.4)	32 (5.5)
Highest FiO2	4177 (95.3)	206	Median (IQR)	0.32 ((0.24 to 0.50)	0.24 (0.21 to 0.38)	0.32 (0.24 to 0.50)
Severity criteria	5974 (99.0)	63	Moderate COVID-19	2165 (60.2)	85 (54.8)	382 (60.5)
			Severe COVID-19	1432 (39.8)	70 (45.2)	249 (39.5)
Critical care admission	6013 (99.6)	24	No	2466 (68.6)	83 (53.5)	440 (69.7)
			Yes	1129 (31.4)	71 (45.8)	183 (29.0)
			(Missing)	NA	NA	8 (1.3)
Invasive ventilation	5940 (98.4)	97	No	3091 (85.9)	126 (81.3)	511 (81.0)
			Yes	480 (13.3)	29 (18.7)	85 (13.5)
			(Missing)	26 (0.7)	NA	35 (5.5)
Level of respiratory support	6037 (100.0)	0	Oxygen alone	2012 (55.9)	85 (54.8)	377 (59.7)
			HFNC	271 (7.5)	16 (10.3)	39 (6.2)
			NIV	834 (23.2)	25 (16.1)	130 (20.6)
			IMV	480 (13.3)	29 (18.7)	85 (13.5)

*Gestational age: 4.5% (7/155) 10-19 weeks, 25.8% (40/155) 20-27 weeks, 25.2% (39/155) 28-32 weeks, 25.8% (40/155), 5.8% (9/155) 37-41 weeks, NA 41-44 weeks, missing gestational age 12.3% (19/155).

Table S8. Multivariable multilevel logistic regression analysis of all adult patients receiving supplementary oxygen with moderate or severe COVID-19 and admitted between the 17th of June 2020 and the 14th of April 2021 (final model n=30670). Here, we have reported the univariable model ("OR (univariable)", multivariable model with all demographic factors and comorbidities included ("OR (multivariable)"), multivariable model where we have selected as few explanatory variables as possible (parsimony) by goodness of fit and their influence on other model variables ("OR (multivariable reduced)") and the final model ("OR (multilevel)"), which includes geographical clustering by random effect of the admitting hospital.

Dependent: Any corticosteroid		No	Yes	OR (univariable)	OR (multivariable)	OR (multivariable reduced)	OR (multilevel)
Illness severity	Moderate COVID-19	5611 (21.0)	21165 (79.0)	-	-	-	-
	Severe COVID-19	3052 (15.6)	16548 (84.4)	1.44 (1.37-1.51, p<0.0001)	1.44 (1.35-1.54, p<0.0001)	1.44 (1.35-1.54, p<0.0001)	1.45 (1.36-1.55, p<0.0001)
Age	<50	861 (12.5)	6006 (87.5)	-	-	-	-
	50-59	914 (11.5)	7037 (88.5)	1.10 (1.00-1.22, p=0.0513)	1.21 (1.05-1.38, p=0.0066)	1.22 (1.07-1.39, p=0.0023)	1.23 (1.08-1.41, p=0.0023)
	60-69	1234 (13.6)	7807 (86.4)	0.91 (0.83-1.00, p=0.0402)	1.12 (0.98-1.27, p=0.0961)	1.13 (1.00-1.28, p=0.0455)	1.09 (0.96-1.23, p=0.2038)
	70-79	2147 (20.1)	8543 (79.9)	0.57 (0.52-0.62, p<0.0001)	0.80 (0.71-0.91, p=0.0008)	0.81 (0.72-0.91, p<0.0005)	0.79 (0.70-0.89, p<0.0001)
	80+	3507 (29.7)	8320 (70.3)	0.34 (0.31-0.37, p<0.0001)	0.53 (0.46-0.60, p<0.0001)	0.53 (0.47-0.60, p<0.0001)	0.52 (0.46-0.58, p<0.0001)
Sex	Female	3984 (20.8)	15141 (79.2)	-	-	-	-
	Male	4668 (17.2)	22518 (82.8)	1.27 (1.21-1.33, p<0.0001)	1.24 (1.16-1.32, p<0.0001)	1.25 (1.17-1.33, p<0.0001)	1.25 (1.17-1.33, p<0.0001)
Ethnicity	White	6473 (19.8)	26261 (80.2)	-	-	-	-
	South Asian	326 (10.5)	2793 (89.5)	2.11 (1.88-2.38, p<0.0001)	1.59 (1.37-1.86, p<0.0001)	1.60 (1.39-1.85, p<0.0001)	1.23 (1.06-1.44, p=0.0079)
	East Asian	37 (15.0)	209 (85.0)	1.39 (0.99-2.01, p=0.0643)	1.04 (0.67-1.69, p=0.8561)	1.04 (0.69-1.63, p=0.8657)	0.98 (0.63-1.52, p=0.9137)
	Black	132 (12.6)	912 (87.4)	1.70 (1.42-2.06, p<0.0001)	1.27 (1.00-1.62, p=0.0559)	1.32 (1.05-1.67, p=0.0214)	1.27 (0.99-1.63, p=0.0599)
	Other ethnic minority	414 (13.7)	2606 (86.3)	1.55 (1.40-1.73, p<0.0001)	1.11 (0.97-1.28, p=0.1295)	1.14 (1.00-1.30, p=0.0518)	1.11 (0.96-1.28, p=0.1480)
IMD quintile	1 (least deprived)	1132 (20.0)	4525 (80.0)	-	-	-	-
	2	1552 (19.4)	6465 (80.6)	1.04 (0.96-1.13, p=0.3447)	0.91 (0.81-1.02, p=0.0944)	0.89 (0.80-1.00, p=0.0451)	0.91 (0.80-1.02, p=0.1033)

	3	1837 (19.4)	7655 (80.6)	1.04 (0.96-1.13, p=0.3241)	0.90 (0.81-1.01, p=0.0740)	0.90 (0.80-1.00, p=0.0470)	0.90 (0.80-1.02, p=0.0980)
	4	1926 (18.1)	8719 (81.9)	1.13 (1.04-1.23, p=0.0028)	1.10 (0.98-1.24, p=0.0898)	1.11 (0.99-1.24, p=0.0634)	0.97 (0.86-1.09, p=0.5954)
	5 (most deprived)	2195 (17.7)	10239 (82.3)	1.17 (1.08-1.26, p=0.0001)	1.01 (0.90-1.13, p=0.8774)	0.99 (0.88-1.10, p=0.7956)	0.90 (0.80-1.02, p=0.0968)
Hypertension	No	3708 (16.5)	18733 (83.5)	-	-	-	-
	Yes	3860 (19.1)	16382 (80.9)	0.84 (0.80-0.88, p<0.0001)	1.00 (0.94-1.07, p=0.9142)	-	-
Chronic cardiac disease	No	4812 (15.6)	26096 (84.4)	-	-	-	-
	Yes	2862 (23.9)	9105 (76.1)	0.59 (0.56-0.62, p<0.0001)	0.85 (0.79-0.92, p<0.0001)	0.84 (0.79-0.91, p<0.0001)	0.83 (0.77-0.90, p<0.0001)
Chronic pulmonary disease	No	5737 (16.7)	28562 (83.3)	-	-	-	-
	Yes	1931 (22.5)	6668 (77.5)	0.69 (0.65-0.74, p<0.0001)	0.80 (0.74-0.87, p<0.0001)	0.81 (0.75-0.87, p<0.0001)	0.79 (0.73-0.86, p<0.0001)
Asthma	No	6497 (18.2)	29245 (81.8)	-	-	-	-
	Yes	1119 (15.8)	5953 (84.2)	1.18 (1.10-1.27, p<0.0001)	1.00 (0.92-1.10, p=0.9681)	-	-
Diabetes	No diabetes	5280 (17.8)	24445 (82.2)	-	-	-	-
	Type 1 diabetes	182 (20.8)	693 (79.2)	0.82 (0.70-0.97, p=0.0210)	0.78 (0.64-0.97, p=0.0209)	-	-
	Type 2 diabetes	2086 (17.8)	9645 (82.2)	1.00 (0.94-1.06, p=0.9634)	1.01 (0.94-1.09, p=0.7246)	-	-
Obesity	No	5705 (18.8)	24651 (81.2)	-	-	-	-
	Yes	889 (11.8)	6674 (88.2)	1.74 (1.61-1.87, p<0.0001)	1.39 (1.26-1.53, p<0.0001)	1.39 (1.27-1.52, p<0.0001)	1.40 (1.27-1.54, p<0.0001)
Chronic neurological disease	No	6566 (17.0)	32028 (83.0)	-	-	-	-
	Yes	1015 (25.4)	2985 (74.6)	0.60 (0.56-0.65, p<0.0001)	0.70 (0.63-0.77, p<0.0001)	0.72 (0.65-0.79, p<0.0001)	0.70 (0.63-0.77, p<0.0001)
Dementia	No	6302 (16.3)	32399 (83.7)	-	-	-	-
	Yes	1229 (32.4)	2559 (67.6)	0.41 (0.38-0.44, p<0.0001)	0.73 (0.65-0.80, p<0.0001)	0.72 (0.66-0.80, p<0.0001)	0.73 (0.66-0.81, p<0.0001)
Chronic kidney disease	No	6125 (16.7)	30470 (83.3)	-	-	-	-

	Yes	1517 (24.7)	4617 (75.3)	0.61 (0.57-0.65, p<0.0001)	0.93 (0.85-1.02, p=0.1250)	-	-
Moderate or severe liver disease	No	7381 (17.6)	34474 (82.4)	-	-	-	-
	Yes	170 (28.8)	421 (71.2)	0.53 (0.44-0.64, p<0.0001)	0.49 (0.38-0.62, p<0.0001)	0.49 (0.39-0.62, p<0.0001)	0.51 (0.40-0.65, p<0.0001)
Mild liver disease	No	7401 (17.8)	34267 (82.2)	-	-	-	-
	Yes	123 (19.1)	522 (80.9)	0.92 (0.75-1.12, p=0.3888)	0.76 (0.59-1.00, p=0.0423)	-	-
Malignancy	No	6730 (17.3)	32274 (82.7)	-	-	-	-
	Yes	873 (24.1)	2742 (75.9)	0.65 (0.60-0.71, p<0.0001)	0.79 (0.72-0.88, p<0.0001)	0.79 (0.72-0.88, p<0.0001)	0.83 (0.75-0.92, p=0.0005)
Rheumatologic disease	No	6465 (17.2)	31062 (82.8)	-	-	-	-
	Yes	1091 (22.0)	3869 (78.0)	0.74 (0.69-0.79, p<0.0001)	0.89 (0.81-0.98, p=0.0197)	-	-
Pre-admission immunosuppressants	No	6760 (17.9)	31073 (82.1)	-	-	-	-
	Yes	780 (15.4)	4299 (84.6)	1.20 (1.11-1.30, p<0.0001)	1.28 (1.15-1.42, p<0.0001)	1.29 (1.16-1.43, p<0.0001)	1.25 (1.13-1.39, p<0.0001)
AIDS/HIV	No	7398 (17.8)	34156 (82.2)	-	-	-	-
	Yes	24 (17.0)	117 (83.0)	1.06 (0.69-1.68, p=0.8085)	0.69 (0.40-1.27, p=0.2089)	-	-
Week admission	Mean (SD)	48.1 (8.3)	50.2 (7.2)	1.04 (1.03-1.04, p<0.0001)	1.04 (1.03-1.04, p<0.0001)	1.04 (1.03-1.04, p<0.0001)	1.04 (1.04-1.05, p<0.0001)
OR multivariable							
Number in dataframe = 46376, Number in model = 29110, Missing = 17266, AIC = 24628.3, C-statistic = 0.687 (95% CI 0.679-0.695), H&L = Chi-sq(8) 8.55 (p=0.382)							
OR multivariable reduced							
Number in dataframe = 46376, Number in model = 31012, Missing = 15364, AIC = 26364.1, C-statistic = 0.686 (95% CI 0.678-0.694), H&L = Chi-sq(8) 10.36 (p=0.240)							
OR multilevel							
Number in model = 30670, Number of groups = 199, AIC = 25092.5, C-statistic = 0.743 (95% CI 0.736-0.751)							

Table S9. Multivariable multilevel logistic regression analysis of all adult patients receiving supplementary oxygen with moderate or severe COVID-19 and admitted between the 17th of June 2020 and the 14th of April 2021, excluding patients with corticosteroid missing (final model n=29974). Here, we have reported the univariable model ("OR (univariable)", multivariable model with all demographic factors and comorbidities included ("OR (multivariable)"), multivariable model where we have selected as few explanatory variables as possible (parsimony) by goodness of fit and their influence on other model variables ("OR (multivariable reduced)") and the final model ("OR (multilevel)"), which includes geographical clustering by random effect of the admitting hospital.

Dependent: Any corticosteroid		No	Yes	OR (univariable)	OR (multivariable)	OR (multivariable reduced)	OR (multilevel)
Illness severity	Moderate COVID-19	4608 (17.9)	21165 (82.1)	-	-	-	-
	Severe COVID-19	2392 (12.6)	16548 (87.4)	1.51 (1.43-1.59, p<0.0001)	1.52 (1.41-1.63, p<0.0001)	1.52 (1.42-1.63, p<0.0001)	1.53 (1.43-1.65, p<0.0001)
Age	<50	619 (9.3)	6006 (90.7)	-	-	-	-
	50-59	626 (8.2)	7037 (91.8)	1.16 (1.03-1.30, p=0.0131)	1.29 (1.11-1.50, p=0.0009)	1.30 (1.12-1.50, p=0.0004)	1.29 (1.11-1.49, p=0.0008)
	60-69	922 (10.6)	7807 (89.4)	0.87 (0.78-0.97, p=0.0128)	1.16 (1.00-1.34, p=0.0481)	1.16 (1.01-1.33, p=0.0361)	1.12 (0.97-1.28, p=0.1229)
	70-79	1733 (16.9)	8543 (83.1)	0.51 (0.46-0.56, p<0.0001)	0.79 (0.68-0.90, p=0.0007)	0.78 (0.69-0.89, p=0.0002)	0.77 (0.67-0.88, p=0.0001)
	80+	3100 (27.1)	8320 (72.9)	0.28 (0.25-0.30, p<0.0001)	0.49 (0.43-0.56, p<0.0001)	0.49 (0.43-0.55, p<0.0001)	0.48 (0.42-0.55, p<0.0001)
Sex	Female	3317 (18.0)	15141 (82.0)	-	-	-	-
	Male	3674 (14.0)	22518 (86.0)	1.34 (1.28-1.41, p<0.0001)	1.29 (1.20-1.38, p<0.0001)	1.31 (1.23-1.40, p<0.0001)	1.30 (1.22-1.40, p<0.0001)
Ethnicity	White	5458 (17.2)	26261 (82.8)	-	-	-	-
	South Asian	280 (9.1)	2793 (90.9)	2.07 (1.83-2.36, p<0.0001)	1.49 (1.27-1.75, p<0.0001)	1.51 (1.30-1.76, p<0.0001)	1.25 (1.07-1.48, p=0.0064)
	East Asian	28 (11.8)	209 (88.2)	1.55 (1.06-2.35, p=0.0295)	1.05 (0.65-1.79, p=0.8446)	1.06 (0.67-1.75, p=0.8158)	1.01 (0.62-1.65, p=0.9614)
	Black	100 (9.9)	912 (90.1)	1.90 (1.55-2.35, p<0.0001)	1.29 (0.99-1.69, p=0.0625)	1.36 (1.06-1.77, p=0.0209)	1.37 (1.04-1.80, p=0.0233)
	Other ethnic minority	298 (10.3)	2606 (89.7)	1.82 (1.61-2.06, p<0.0001)	1.24 (1.06-1.44, p=0.0066)	1.28 (1.11-1.49, p=0.0011)	1.25 (1.06-1.46, p=0.0069)
IMD quintile	1 (least deprived)	942 (17.2)	4525 (82.8)	-	-	-	-

	2	1297 (16.7)	6465 (83.3)	1.04 (0.95-1.14, p=0.4313)	0.91 (0.80-1.03, p=0.1361)	0.90 (0.80-1.02, p=0.0970)	0.91 (0.80-1.03, p=0.1524)
	3	1554 (16.9)	7655 (83.1)	1.03 (0.94-1.12, p=0.5791)	0.90 (0.80-1.02, p=0.1010)	0.89 (0.79-1.00, p=0.0484)	0.90 (0.80-1.03, p=0.1164)
	4	1378 (13.6)	8719 (86.4)	1.32 (1.20-1.44, p<0.0001)	1.13 (1.00-1.28, p=0.0511)	1.12 (1.00-1.26, p=0.0579)	0.98 (0.87-1.12, p=0.8045)
	5 (most deprived)	1813 (15.0)	10239 (85.0)	1.18 (1.08-1.28, p=0.0002)	1.04 (0.93-1.17, p=0.4784)	1.02 (0.91-1.14, p=0.7571)	0.92 (0.81-1.04, p=0.1829)
Hypertension	No	3106 (14.2)	18733 (85.8)	-	-	-	-
	Yes	3371 (17.1)	16382 (82.9)	0.81 (0.76-0.85, p<0.0001)	1.00 (0.93-1.08, p=0.8936)	-	-
Chronic cardiac disease	No	3962 (13.2)	26096 (86.8)	-	-	-	-
	Yes	2578 (22.1)	9105 (77.9)	0.54 (0.51-0.57, p<0.0001)	0.83 (0.76-0.89, p<0.0001)	0.81 (0.76-0.88, p<0.0001)	0.82 (0.75-0.88, p<0.0001)
Chronic pulmonary disease	No	4817 (14.4)	28562 (85.6)	-	-	-	-
	Yes	1718 (20.5)	6668 (79.5)	0.65 (0.62-0.70, p<0.0001)	0.77 (0.71-0.84, p<0.0001)	0.78 (0.72-0.84, p<0.0001)	0.76 (0.70-0.83, p<0.0001)
Asthma	No	5548 (15.9)	29245 (84.1)	-	-	-	-
	Yes	947 (13.7)	5953 (86.3)	1.19 (1.11-1.29, p<0.0001)	1.00 (0.91-1.10, p=0.9531)	-	-
Diabetes	No diabetes	4501 (15.5)	24445 (84.5)	-	-	-	-
	Type 1 diabetes	145 (17.3)	693 (82.7)	0.88 (0.74-1.06, p=0.1681)	0.88 (0.70-1.11, p=0.2647)	-	-
	Type 2 diabetes	1798 (15.7)	9645 (84.3)	0.99 (0.93-1.05, p=0.6841)	1.01 (0.93-1.10, p=0.7457)	-	-
Obesity	No	4864 (16.5)	24651 (83.5)	-	-	-	-
	Yes	726 (9.8)	6674 (90.2)	1.81 (1.67-1.97, p<0.0001)	1.40 (1.26-1.55, p<0.0001)	1.39 (1.26-1.53, p<0.0001)	1.40 (1.26-1.55, p<0.0001)
Chronic neurological disease	No	5542 (14.8)	32028 (85.2)	-	-	-	-
	Yes	919 (23.5)	2985 (76.5)	0.56 (0.52-0.61, p<0.0001)	0.67 (0.60-0.74, p<0.0001)	0.68 (0.62-0.76, p<0.0001)	0.67 (0.60-0.74, p<0.0001)
Dementia	No	5271 (14.0)	32399 (86.0)	-	-	-	-

	Yes	1144 (30.9)	2559 (69.1)	0.36 (0.34-0.39, p<0.0001)	0.70 (0.63-0.77, p<0.0001)	0.69 (0.63-0.77, p<0.0001)	0.71 (0.64-0.78, p<0.0001)
Chronic kidney disease	No	5143 (14.4)	30470 (85.6)	-	-	-	-
	Yes	1366 (22.8)	4617 (77.2)	0.57 (0.53-0.61, p<0.0001)	0.92 (0.84-1.01, p=0.0737)	-	-
Moderate or severe liver disease	No	6276 (15.4)	34474 (84.6)	-	-	-	-
	Yes	154 (26.8)	421 (73.2)	0.50 (0.41-0.60, p<0.0001)	0.46 (0.36-0.59, p<0.0001)	0.46 (0.36-0.59, p<0.0001)	0.48 (0.37-0.61, p<0.0001)
Mild liver disease	No	6300 (15.5)	34267 (84.5)	-	-	-	-
	Yes	105 (16.7)	522 (83.3)	0.91 (0.74-1.13, p=0.4043)	0.74 (0.57-0.98, p=0.0318)	-	-
Malignancy	No	5683 (15.0)	32274 (85.0)	-	-	-	-
	Yes	795 (22.5)	2742 (77.5)	0.61 (0.56-0.66, p<0.0001)	0.75 (0.67-0.84, p<0.0001)	0.75 (0.67-0.83, p<0.0001)	0.79 (0.71-0.89, p<0.0001)
Rheumatologic disease	No	5465 (15.0)	31062 (85.0)	-	-	-	-
	Yes	974 (20.1)	3869 (79.9)	0.70 (0.65-0.75, p<0.0001)	0.87 (0.79-0.96, p=0.0063)	-	-
Pre-admission immunosuppressants	No	5826 (15.8)	31073 (84.2)	-	-	-	-
	Yes	678 (13.6)	4299 (86.4)	1.19 (1.09-1.30, p=0.0001)	1.31 (1.17-1.46, p<0.0001)	1.31 (1.17-1.46, p<0.0001)	1.28 (1.14-1.43, p<0.0001)
AIDS/HIV	No	6299 (15.6)	34156 (84.4)	-	-	-	-
	Yes	21 (15.2)	117 (84.8)	1.03 (0.66-1.68, p=0.9091)	0.74 (0.40-1.46, p=0.3538)	-	-
Week admission	Mean (SD)	47.6 (8.3)	50.2 (7.2)	1.04 (1.04-1.05, p<0.0001)	1.04 (1.04-1.04, p<0.0001)	1.04 (1.04-1.04, p<0.0001)	1.05 (1.04-1.05, p<0.0001)
OR multivariable							
Number in dataframe = 44713, Number in model = 28488, Missing = 16225, AIC = 22122.2, C-statistic = 0.709 (95% CI 0.701-0.717), H&L = Chi-sq(8) 16.15 (p=0.040)							
OR multivariable reduced							
Number in dataframe = 44713, Number in model = 30306, Missing = 14407, AIC = 23547.3, C-statistic = 0.709 (95% CI 0.701-0.717), H&L = Chi-sq(8) 20.09 (p=0.010)							
OR multilevel							
Number in model = 29974, Number of groups = 196, AIC = 22632.8, C-statistic = 0.753 (95% CI 0.745-0.760)							

Table S10. Multivariable multilevel logistic regression analysis of only PCR positive adult patients receiving supplementary oxygen with moderate or severe COVID-19 and admitted between the 17th of June 2020 and the 14th of April 2021 (final model n=27384). Here, we have reported the univariable model ("OR (univariable)", multivariable model with all demographic factors and comorbidities included ("OR (multivariable)"), multivariable model where we have selected as few explanatory variables as possible (parsimony) by goodness of fit and their influence on other model variables ("OR (multivariable reduced)") and the final model ("OR (multilevel)"), which includes geographical clustering by random effect of the admitting hospital.

Dependent: Any corticosteroid		No	Yes	OR (univariable)	OR (multivariable)	OR (multivariable reduced)	OR (multilevel)
Illness severity	Moderate COVID-19	4648 (19.5)	19197 (80.5)	-	-	-	-
	Severe COVID-19	2406 (13.9)	14961 (86.1)	1.51 (1.43-1.59, p<0.0001)	1.51 (1.40-1.62, p<0.0001)	1.52 (1.41-1.63, p<0.0001)	1.52 (1.41-1.64, p<0.0001)
Age	<50	630 (10.5)	5357 (89.5)	-	-	-	-
	50-59	696 (9.8)	6408 (90.2)	1.08 (0.97-1.21, p=0.1705)	1.23 (1.06-1.44, p=0.0074)	1.24 (1.07-1.44, p=0.0042)	1.23 (1.06-1.43, p=0.0073)
	60-69	956 (11.9)	7087 (88.1)	0.87 (0.78-0.97, p=0.0117)	1.11 (0.96-1.29, p=0.1714)	1.12 (0.97-1.29, p=0.1215)	1.08 (0.93-1.25, p=0.3141)
	70-79	1733 (18.3)	7760 (81.7)	0.53 (0.48-0.58, p<0.0001)	0.78 (0.67-0.90, p=0.0006)	0.78 (0.68-0.89, p=0.0002)	0.77 (0.67-0.88, p=0.0002)
	80+	3039 (28.7)	7546 (71.3)	0.29 (0.27-0.32, p<0.0001)	0.49 (0.42-0.57, p<0.0001)	0.48 (0.42-0.55, p<0.0001)	0.48 (0.42-0.55, p<0.0001)
Sex	Female	3310 (19.4)	13709 (80.6)	-	-	-	-
	Male	3737 (15.5)	20403 (84.5)	1.32 (1.25-1.39, p<0.0001)	1.28 (1.20-1.38, p<0.0001)	1.30 (1.21-1.39, p<0.0001)	1.30 (1.21-1.39, p<0.0001)
Ethnicity	White	5341 (18.3)	23825 (81.7)	-	-	-	-
	South Asian	271 (9.7)	2528 (90.3)	2.09 (1.84-2.38, p<0.0001)	1.51 (1.29-1.78, p<0.0001)	1.52 (1.30-1.79, p<0.0001)	1.26 (1.06-1.49, p=0.0072)
	East Asian	29 (13.1)	192 (86.9)	1.48 (1.02-2.24, p=0.0481)	0.95 (0.60-1.60, p=0.8368)	0.98 (0.62-1.63, p=0.9321)	0.95 (0.58-1.55, p=0.8271)
	Black	98 (10.6)	823 (89.4)	1.88 (1.53-2.34, p<0.0001)	1.45 (1.10-1.94, p=0.0097)	1.48 (1.13-1.96, p=0.0052)	1.48 (1.11-1.97, p=0.0076)
	Other ethnic minority	293 (11.3)	2306 (88.7)	1.76 (1.56-2.00, p<0.0001)	1.27 (1.08-1.49, p=0.0034)	1.31 (1.12-1.53, p=0.0007)	1.28 (1.08-1.51, p=0.0035)
IMD quintile	1 (least deprived)	913 (18.4)	4062 (81.6)	-	-	-	-
	2	1204 (17.3)	5757 (82.7)	1.07 (0.98-1.18, p=0.1367)	0.91 (0.80-1.03, p=0.1527)	0.91 (0.80-1.03, p=0.1276)	0.92 (0.80-1.05, p=0.1997)

	3	1509 (17.8)	6951 (82.2)	1.04 (0.95-1.13, p=0.4535)	0.86 (0.76-0.97, p=0.0140)	0.85 (0.75-0.95, p=0.0064)	0.86 (0.76-0.98, p=0.0235)
	4	1619 (17.1)	7832 (82.9)	1.09 (0.99-1.19, p=0.0669)	1.07 (0.95-1.22, p=0.2727)	1.06 (0.94-1.20, p=0.3217)	0.95 (0.83-1.08, p=0.4451)
	5 (most deprived)	1798 (16.0)	9458 (84.0)	1.18 (1.08-1.29, p=0.0002)	1.01 (0.89-1.14, p=0.8604)	0.99 (0.88-1.11, p=0.8725)	0.89 (0.78-1.01, p=0.0783)
Hypertension	No	2996 (14.9)	17120 (85.1)	-	-	-	-
	Yes	3230 (17.7)	14996 (82.3)	0.81 (0.77-0.86, p<0.0001)	1.01 (0.94-1.09, p=0.8347)	-	-
Chronic cardiac disease	No	3781 (13.8)	23688 (86.2)	-	-	-	-
	Yes	2427 (22.6)	8317 (77.4)	0.55 (0.52-0.58, p<0.0001)	0.82 (0.76-0.89, p<0.0001)	0.82 (0.76-0.88, p<0.0001)	0.81 (0.75-0.88, p<0.0001)
Chronic pulmonary disease	No	4584 (15.0)	25970 (85.0)	-	-	-	-
	Yes	1616 (21.1)	6060 (78.9)	0.66 (0.62-0.71, p<0.0001)	0.78 (0.72-0.85, p<0.0001)	0.78 (0.72-0.85, p<0.0001)	0.77 (0.70-0.83, p<0.0001)
Asthma	No	5273 (16.5)	26612 (83.5)	-	-	-	-
	Yes	894 (14.2)	5383 (85.8)	1.19 (1.11-1.29, p<0.0001)	1.02 (0.93-1.13, p=0.6794)	-	-
Diabetes	No diabetes	4269 (16.0)	22446 (84.0)	-	-	-	-
	Type 1 diabetes	123 (16.8)	609 (83.2)	0.94 (0.78-1.15, p=0.5489)	0.90 (0.71-1.15, p=0.4024)	-	-
	Type 2 diabetes	1758 (16.6)	8846 (83.4)	0.96 (0.90-1.02, p=0.1562)	1.00 (0.92-1.09, p=0.9775)	-	-
Obesity	No	4622 (17.1)	22341 (82.9)	-	-	-	-
	Yes	685 (10.1)	6097 (89.9)	1.84 (1.69-2.01, p<0.0001)	1.41 (1.27-1.57, p<0.0001)	1.40 (1.27-1.55, p<0.0001)	1.41 (1.27-1.57, p<0.0001)
Chronic neurological disease	No	5261 (15.3)	29099 (84.7)	-	-	-	-
	Yes	872 (24.1)	2753 (75.9)	0.57 (0.53-0.62, p<0.0001)	0.68 (0.61-0.76, p<0.0001)	0.70 (0.63-0.77, p<0.0001)	0.68 (0.61-0.76, p<0.0001)
Dementia	No	5013 (14.5)	29485 (85.5)	-	-	-	-
	Yes	1078 (31.9)	2305 (68.1)	0.36 (0.34-0.39, p<0.0001)	0.69 (0.62-0.76, p<0.0001)	0.69 (0.62-0.76, p<0.0001)	0.69 (0.62-0.77, p<0.0001)
Chronic kidney disease	No	4879 (15.0)	27669 (85.0)	-	-	-	-

	Yes	1299 (23.4)	4245 (76.6)	0.58 (0.54-0.62, p<0.0001)	0.92 (0.84-1.01, p=0.0779)	-	-
Moderate or severe liver disease	No	5965 (16.0)	31379 (84.0)	-	-	-	-
	Yes	140 (27.2)	375 (72.8)	0.51 (0.42-0.62, p<0.0001)	0.47 (0.36-0.61, p<0.0001)	0.46 (0.36-0.60, p<0.0001)	0.48 (0.37-0.63, p<0.0001)
Mild liver disease	No	5982 (16.1)	31171 (83.9)	-	-	-	-
	Yes	99 (16.9)	488 (83.1)	0.95 (0.76-1.18, p=0.6172)	0.74 (0.57-0.99, p=0.0370)	-	-
Malignancy	No	5397 (15.5)	29313 (84.5)	-	-	-	-
	Yes	752 (22.9)	2538 (77.1)	0.62 (0.57-0.68, p<0.0001)	0.75 (0.67-0.83, p<0.0001)	0.74 (0.66-0.83, p<0.0001)	0.79 (0.71-0.89, p<0.0001)
Rheumatologic disease	No	5206 (15.6)	28249 (84.4)	-	-	-	-
	Yes	907 (20.5)	3524 (79.5)	0.72 (0.66-0.78, p<0.0001)	0.88 (0.80-0.98, p=0.0178)	-	-
Pre-admission immunosuppressants	No	5480 (16.3)	28176 (83.7)	-	-	-	-
	Yes	635 (14.0)	3894 (86.0)	1.19 (1.09-1.30, p=0.0001)	1.29 (1.15-1.45, p<0.0001)	1.29 (1.15-1.44, p<0.0001)	1.26 (1.13-1.42, p=0.0001)
AIDS/HIV	No	5991 (16.2)	31104 (83.8)	-	-	-	-
	Yes	17 (14.3)	102 (85.7)	1.16 (0.71-2.00, p=0.5812)	0.80 (0.42-1.67, p=0.5290)	-	-
Week admission	Mean (SD)	48.4 (7.9)	50.2 (7.0)	1.03 (1.03-1.04, p<0.0001)	1.04 (1.03-1.04, p<0.0001)	1.04 (1.03-1.04, p<0.0001)	1.04 (1.04-1.05, p<0.0001)
OR multivariable							
Number in dataframe = 41212, Number in model = 26465, Missing = 14747, AIC = 20997.5, C-statistic = 0.703 (95% CI 0.694-0.712), H&L = Chi-sq(8) 15.15 (p=0.056)							
OR multivariable reduced							
Number in dataframe = 41212, Number in model = 27696, Missing = 13516, AIC = 22026.3, C-statistic = 0.702 (95% CI 0.694-0.711), H&L = Chi-sq(8) 19.45 (p=0.013)							
OR multilevel							
Number in model = 27384, Number of groups = 195, AIC = 21112.8, C-statistic = 0.751 (95% CI 95% CI: 0.746-0.759)							

Table S11. Multivariable multilevel logistic regression analysis of patients who received supplementary oxygen with moderate or severe COVID-19 and were admitted between the 17th of June 2020 and the 14th of April 2021, excluding participants with outcome of death or palliative discharge recorded within two days of admission (final model n=29979). Here, we have reported the univariable model ("OR (univariable)", multivariable model with all demographic factors and comorbidities included ("OR (multivariable)"), multivariable model where we have selected as few explanatory variables as possible (parsimony) by goodness of fit and their influence on other model variables ("OR (multivariable reduced)") and the final model ("OR (multilevel)"), which includes geographical clustering by random effect of the admitting hospital.

Dependent: Any corticosteroid		No	Yes	OR (univariable)	OR (multivariable)	OR (multivariable reduced)	OR (multilevel)
Illness severity	Moderate COVID-19	5516 (20.9)	20935 (79.1)	-	-	-	-
	Severe COVID-19	2819 (15.0)	15957 (85.0)	1.49 (1.42-1.57, p<0.0001)	1.48 (1.38-1.59, p<0.0001)	1.48 (1.39-1.58, p<0.0001)	1.50 (1.40-1.61, p<0.0001)
Age	<50	855 (12.5)	5979 (87.5)	-	-	-	-
	50-59	905 (11.5)	6996 (88.5)	1.11 (1.00-1.22, p=0.0486)	1.20 (1.05-1.38, p=0.0075)	1.22 (1.07-1.39, p=0.0026)	1.23 (1.08-1.41, p=0.0024)
	60-69	1202 (13.5)	7687 (86.5)	0.91 (0.83-1.00, p=0.0623)	1.13 (0.99-1.29, p=0.0692)	1.15 (1.01-1.30, p=0.0309)	1.10 (0.97-1.25, p=0.1430)
	70-79	2059 (19.8)	8317 (80.2)	0.58 (0.53-0.63, p<0.0001)	0.81 (0.71-0.92, p=0.0015)	0.82 (0.73-0.92, p=0.0011)	0.80 (0.71-0.90, p=0.0004)
	80+	3314 (29.5)	7913 (70.5)	0.34 (0.31-0.37, p<0.0001)	0.53 (0.46-0.60, p<0.0001)	0.53 (0.47-0.60, p<0.0001)	0.52 (0.46-0.59, p<0.0001)
Sex	Female	3835 (20.6)	14802 (79.4)	-	-	-	-
	Male	4490 (16.9)	22036 (83.1)	1.27 (1.21-1.33, p<0.0001)	1.24 (1.16-1.33, p<0.0001)	1.26 (1.18-1.34, p<0.0001)	1.26 (1.18-1.34, p<0.0001)
Ethnicity	White	6234 (19.5)	25663 (80.5)	-	-	-	-
	South Asian	302 (9.9)	2736 (90.1)	2.20 (1.95-2.49, p<0.0001)	1.63 (1.40-1.91, p<0.0001)	1.64 (1.42-1.90, p<0.0001)	1.26 (1.07-1.47, p=0.0045)
	East Asian	35 (14.8)	202 (85.2)	1.40 (0.99-2.04, p=0.0658)	1.03 (0.66-1.70, p=0.8859)	1.03 (0.68-1.63, p=0.9024)	0.96 (0.61-1.51, p=0.8696)
	Black	123 (12.0)	900 (88.0)	1.78 (1.48-2.16, p<0.0001)	1.30 (1.02-1.68, p=0.0367)	1.35 (1.07-1.73, p=0.0136)	1.29 (1.00-1.67, p=0.0476)
	Other ethnic minority	401 (13.5)	2564 (86.5)	1.55 (1.39-1.73, p<0.0001)	1.12 (0.97-1.28, p=0.1211)	1.14 (1.00-1.31, p=0.0495)	1.12 (0.97-1.29, p=0.1241)
IMD quintile	1 (least deprived)	1085 (19.7)	4430 (80.3)	-	-	-	-

	2	1502 (19.1)	6342 (80.9)	1.03 (0.95-1.13, p=0.4494)	0.90 (0.80-1.01, p=0.0704)	0.89 (0.79-0.99, p=0.0388)	0.90 (0.80-1.01, p=0.0798)
	3	1760 (19.0)	7489 (81.0)	1.04 (0.96-1.13, p=0.3368)	0.89 (0.79-1.00, p=0.0491)	0.89 (0.79-0.99, p=0.0367)	0.89 (0.79-1.01, p=0.0672)
	4	1858 (17.9)	8528 (82.1)	1.12 (1.03-1.22, p=0.0058)	1.11 (0.98-1.24, p=0.0957)	1.11 (0.99-1.25, p=0.0605)	0.96 (0.85-1.09, p=0.5626)
	5 (most deprived)	2109 (17.4)	9993 (82.6)	1.16 (1.07-1.26, p=0.0003)	1.00 (0.89-1.12, p=0.9768)	0.98 (0.88-1.09, p=0.7149)	0.89 (0.79-1.01, p=0.0714)
Hypertension	No	3588 (16.3)	18421 (83.7)	-	-	-	-
	Yes	3696 (18.8)	15941 (81.2)	0.84 (0.80-0.88, p<0.0001)	1.00 (0.93-1.07, p=0.9544)	-	-
Chronic cardiac disease	No	4672 (15.4)	25695 (84.6)	-	-	-	-
	Yes	2715 (23.7)	8742 (76.3)	0.59 (0.56-0.62, p<0.0001)	0.86 (0.79-0.92, p=0.0001)	0.85 (0.79-0.91, p<0.0001)	0.83 (0.77-0.90, p<0.0001)
Chronic pulmonary disease	No	5531 (16.5)	28017 (83.5)	-	-	-	-
	Yes	1850 (22.3)	6455 (77.7)	0.69 (0.65-0.73, p<0.0001)	0.80 (0.73-0.86, p<0.0001)	0.80 (0.74-0.87, p<0.0001)	0.79 (0.73-0.85, p<0.0001)
Asthma	No	6249 (17.9)	28607 (82.1)	-	-	-	-
	Yes	1087 (15.7)	5840 (84.3)	1.17 (1.09-1.26, p<0.0001)	1.00 (0.91-1.10, p=0.9713)	-	-
Diabetes	No diabetes	5105 (17.5)	24007 (82.5)	-	-	-	-
	Type 1 diabetes	174 (20.5)	675 (79.5)	0.82 (0.70-0.98, p=0.0259)	0.78 (0.63-0.96, p=0.0184)	-	-
	Type 2 diabetes	1987 (17.5)	9358 (82.5)	1.00 (0.95-1.06, p=0.9594)	1.02 (0.94-1.10, p=0.6474)	-	-
Obesity	No	5499 (18.6)	24111 (81.4)	-	-	-	-
	Yes	868 (11.7)	6574 (88.3)	1.73 (1.60-1.87, p<0.0001)	1.38 (1.25-1.52, p<0.0001)	1.38 (1.26-1.51, p<0.0001)	1.39 (1.26-1.53, p<0.0001)
Chronic neurological disease	No	6321 (16.8)	31373 (83.2)	-	-	-	-
	Yes	974 (25.2)	2888 (74.8)	0.60 (0.55-0.65, p<0.0001)	0.69 (0.62-0.77, p<0.0001)	0.71 (0.65-0.79, p<0.0001)	0.70 (0.63-0.77, p<0.0001)
Dementia	No	6101 (16.1)	31766 (83.9)	-	-	-	-

	Yes	1148 (31.9)	2450 (68.1)	0.41 (0.38-0.44, p<0.0001)	0.74 (0.67-0.82, p<0.0001)	0.74 (0.67-0.82, p<0.0001)	0.75 (0.68-0.83, p<0.0001)
Chronic kidney disease	No	5920 (16.5)	29914 (83.5)	-	-	-	-
	Yes	1435 (24.5)	4419 (75.5)	0.61 (0.57-0.65, p<0.0001)	0.92 (0.84-1.01, p=0.0870)	-	-
Moderate or severe liver disease	No	7102 (17.4)	33735 (82.6)	-	-	-	-
	Yes	167 (29.0)	409 (71.0)	0.52 (0.43-0.62, p<0.0001)	0.49 (0.39-0.63, p<0.0001)	0.49 (0.39-0.63, p<0.0001)	0.52 (0.41-0.66, p<0.0001)
Mild liver disease	No	7121 (17.5)	33533 (82.5)	-	-	-	-
	Yes	120 (19.1)	508 (80.9)	0.90 (0.74-1.10, p=0.2980)	0.75 (0.58-0.99, p=0.0356)	-	-
Malignancy	No	6501 (17.1)	31624 (82.9)	-	-	-	-
	Yes	819 (23.7)	2639 (76.3)	0.66 (0.61-0.72, p<0.0001)	0.80 (0.72-0.89, p=0.0001)	0.80 (0.72-0.89, p<0.0001)	0.84 (0.75-0.93, p=0.0014)
Rheumatologic disease	No	6218 (17.0)	30432 (83.0)	-	-	-	-
	Yes	1056 (22.0)	3753 (78.0)	0.73 (0.67-0.78, p<0.0001)	0.88 (0.80-0.97, p=0.0086)	-	-
Pre-admission immunosuppressants	No	6503 (17.6)	30448 (82.4)	-	-	-	-
	Yes	752 (15.3)	4174 (84.7)	1.19 (1.09-1.29, p<0.0001)	1.27 (1.14-1.42, p<0.0001)	1.28 (1.15-1.42, p<0.0001)	1.24 (1.12-1.38, p=0.0001)
AIDS/HIV	No	7122 (17.6)	33415 (82.4)	-	-	-	-
	Yes	23 (16.5)	116 (83.5)	1.07 (0.70-1.72, p=0.7519)	0.68 (0.39-1.25, p=0.1928)	-	-
Week admission	Mean (SD)	48.1 (8.3)	50.2 (7.2)	1.04 (1.03-1.04, p<0.0001)	1.04 (1.03-1.04, p<0.0001)	1.04 (1.03-1.04, p<0.0001)	1.04 (1.04-1.05, p<0.0001)
OR multivariable							
Number in dataframe = 45227, Number in model = 28462, Missing = 16765, AIC = 23872.2, C-statistic = 0.688 (95% CI 0.680-0.697), H&L = Chi-sq(8) 9.37 (p=0.312)							
OR multivariable reduced							
Number in dataframe = 45227, Number in model = 30317, Missing = 14910, AIC = 25549, C-statistic = 0.688 (95% CI 0.680-0.696), H&L = Chi-sq(8) 14.28 (p=0.075)							
OR multilevel							
Number in model = 29979, Number of groups = 199, AIC = 24277.6, C-statistic = 0.746 (95% CI 0.739-0.754)							

Table S12. Multivariable multilevel logistic regression analysis of patients who received supplementary oxygen with moderate or severe COVID-19 and were admitted between the 17th of June 2020 and the 14th of April 2021, including clinical frailty categories in the model (final model n=16902). Here, we have reported the univariable model ("OR (univariable)", multivariable model with all demographic factors and comorbidities included ("OR (multivariable)"), multivariable model where we have selected as few explanatory variables as possible (parsimony) by goodness of fit and their influence on other model variables ("OR (multivariable reduced)") and the final model ("OR (multilevel)"), which includes geographical clustering by random effect of the admitting hospital.

Dependent: Any corticosteroid		No	Yes	OR (univariable)	OR (multivariable)	OR (multivariable reduced)	OR (multilevel)
Illness severity	Moderate COVID-19	5611 (21.0)	21165 (79.0)	-	-	-	-
	Severe COVID-19	3052 (15.6)	16548 (84.4)	1.44 (1.37-1.51, p<0.0001)	1.48 (1.35-1.62, p<0.0001)	1.48 (1.36-1.62, p<0.0001)	1.51 (1.38-1.66, p<0.0001)
Age	<50	861 (12.5)	6006 (87.5)	-	-	-	-
	50-59	914 (11.5)	7037 (88.5)	1.10 (1.00-1.22, p=0.0513)	1.45 (1.19-1.76, p=0.0002)	1.47 (1.22-1.77, p=0.0001)	1.43 (1.18-1.74, p=0.0003)
	60-69	1234 (13.6)	7807 (86.4)	0.91 (0.83-1.00, p=0.0402)	1.51 (1.25-1.83, p<0.0001)	1.50 (1.25-1.80, p<0.0001)	1.40 (1.16-1.70, p=0.0004)
	70-79	2147 (20.1)	8543 (79.9)	0.57 (0.52-0.62, p<0.0001)	1.11 (0.92-1.33, p=0.2965)	1.11 (0.93-1.33, p=0.2426)	1.06 (0.88-1.28, p=0.5099)
	80+	3507 (29.7)	8320 (70.3)	0.34 (0.31-0.37, p<0.0001)	0.83 (0.68-1.01, p=0.0578)	0.82 (0.68-0.99, p=0.0351)	0.80 (0.66-0.97, p=0.0215)
Sex	Female	3984 (20.8)	15141 (79.2)	-	-	-	-
	Male	4668 (17.2)	22518 (82.8)	1.27 (1.21-1.33, p<0.0001)	1.24 (1.13-1.35, p<0.0001)	1.25 (1.15-1.36, p<0.0001)	1.25 (1.14-1.37, p<0.0001)
Ethnicity	White	6473 (19.8)	26261 (80.2)	-	-	-	-
	South Asian	326 (10.5)	2793 (89.5)	2.11 (1.88-2.38, p<0.0001)	1.58 (1.30-1.94, p<0.0001)	1.60 (1.33-1.95, p<0.0001)	1.18 (0.96-1.45, p=0.1168)
	East Asian	37 (15.0)	209 (85.0)	1.39 (0.99-2.01, p=0.0643)	0.93 (0.53-1.74, p=0.8042)	0.90 (0.53-1.62, p=0.7091)	0.84 (0.48-1.47, p=0.5382)
	Black	132 (12.6)	912 (87.4)	1.70 (1.42-2.06, p<0.0001)	1.18 (0.84-1.71, p=0.3467)	1.24 (0.89-1.77, p=0.2246)	1.08 (0.75-1.55, p=0.6778)
	Other ethnic minority	414 (13.7)	2606 (86.3)	1.55 (1.40-1.73, p<0.0001)	1.31 (1.07-1.62, p=0.0099)	1.27 (1.05-1.56, p=0.0174)	1.14 (0.92-1.42, p=0.2188)
IMD quintile	1 (least deprived)	1132 (20.0)	4525 (80.0)	-	-	-	-

	2	1552 (19.4)	6465 (80.6)	1.04 (0.96-1.13, p=0.3447)	0.94 (0.80-1.10, p=0.4470)	0.93 (0.80-1.08, p=0.3548)	0.95 (0.81-1.12, p=0.5604)
	3	1837 (19.4)	7655 (80.6)	1.04 (0.96-1.13, p=0.3241)	0.93 (0.80-1.09, p=0.3790)	0.93 (0.80-1.08, p=0.3576)	0.91 (0.77-1.07, p=0.2575)
	4	1926 (18.1)	8719 (81.9)	1.13 (1.04-1.23, p=0.0028)	1.22 (1.04-1.43, p=0.0123)	1.20 (1.03-1.40, p=0.0179)	1.02 (0.86-1.20, p=0.8382)
	5 (most deprived)	2195 (17.7)	10239 (82.3)	1.17 (1.08-1.26, p=0.0001)	1.09 (0.93-1.26, p=0.2815)	1.06 (0.92-1.23, p=0.4162)	0.94 (0.80-1.11, p=0.5008)
Clinical frailty	1-2	659 (9.4)	6319 (90.6)	-	-	-	-
	3-4	1020 (13.1)	6765 (86.9)	0.69 (0.62-0.77, p<0.0001)	0.86 (0.75-1.00, p=0.0430)	0.86 (0.75-0.99, p=0.0390)	0.88 (0.76-1.02, p=0.0884)
	5-6	1323 (24.7)	4038 (75.3)	0.32 (0.29-0.35, p<0.0001)	0.52 (0.44-0.61, p<0.0001)	0.52 (0.45-0.61, p<0.0001)	0.53 (0.45-0.63, p<0.0001)
	7-9	1024 (31.4)	2239 (68.6)	0.23 (0.20-0.25, p<0.0001)	0.42 (0.35-0.50, p<0.0001)	0.42 (0.36-0.50, p<0.0001)	0.41 (0.34-0.49, p<0.0001)
Hypertension	No	3708 (16.5)	18733 (83.5)	-	-	-	-
	Yes	3860 (19.1)	16382 (80.9)	0.84 (0.80-0.88, p<0.0001)	0.99 (0.90-1.09, p=0.8491)	-	-
Chronic cardiac disease	No	4812 (15.6)	26096 (84.4)	-	-	-	-
	Yes	2862 (23.9)	9105 (76.1)	0.59 (0.56-0.62, p<0.0001)	0.89 (0.80-0.98, p=0.0213)	0.88 (0.80-0.97, p=0.0131)	0.88 (0.79-0.97, p=0.0120)
Chronic pulmonary disease	No	5737 (16.7)	28562 (83.3)	-	-	-	-
	Yes	1931 (22.5)	6668 (77.5)	0.69 (0.65-0.74, p<0.0001)	0.84 (0.76-0.94, p=0.0023)	0.85 (0.76-0.94, p=0.0018)	0.82 (0.73-0.91, p=0.0004)
Asthma	No	6497 (18.2)	29245 (81.8)	-	-	-	-
	Yes	1119 (15.8)	5953 (84.2)	1.18 (1.10-1.27, p<0.0001)	1.01 (0.89-1.15, p=0.8499)	-	-
Diabetes	No diabetes	5280 (17.8)	24445 (82.2)	-	-	-	-
	Type 1 diabetes	182 (20.8)	693 (79.2)	0.82 (0.70-0.97, p=0.0210)	0.70 (0.53-0.93, p=0.0132)	-	-
	Type 2 diabetes	2086 (17.8)	9645 (82.2)	1.00 (0.94-1.06, p=0.9634)	1.05 (0.94-1.16, p=0.3874)	-	-
Obesity	No	5705 (18.8)	24651 (81.2)	-	-	-	-

	Yes	889 (11.8)	6674 (88.2)	1.74 (1.61-1.87, p<0.0001)	1.31 (1.16-1.49, p<0.0001)	1.32 (1.17-1.49, p<0.0001)	1.35 (1.19-1.54, p<0.0001)
Chronic neurological disease	No	6566 (17.0)	32028 (83.0)	-	-	-	-
	Yes	1015 (25.4)	2985 (74.6)	0.60 (0.56-0.65, p<0.0001)	0.84 (0.74-0.96, p=0.0100)	0.85 (0.75-0.97, p=0.0159)	0.84 (0.73-0.96, p=0.0105)
Dementia	No	6302 (16.3)	32399 (83.7)	-	-	-	-
	Yes	1229 (32.4)	2559 (67.6)	0.41 (0.38-0.44, p<0.0001)	0.82 (0.72-0.94, p=0.0044)	0.84 (0.73-0.96, p=0.0093)	0.82 (0.72-0.95, p=0.0059)
Chronic kidney disease	No	6125 (16.7)	30470 (83.3)	-	-	-	-
	Yes	1517 (24.7)	4617 (75.3)	0.61 (0.57-0.65, p<0.0001)	0.97 (0.86-1.09, p=0.6020)	-	-
Moderate or severe liver disease	No	7381 (17.6)	34474 (82.4)	-	-	-	-
	Yes	170 (28.8)	421 (71.2)	0.53 (0.44-0.64, p<0.0001)	0.48 (0.35-0.66, p<0.0001)	0.47 (0.35-0.64, p<0.0001)	0.51 (0.37-0.70, p<0.0001)
Mild liver disease	No	7401 (17.8)	34267 (82.2)	-	-	-	-
	Yes	123 (19.1)	522 (80.9)	0.92 (0.75-1.12, p=0.3888)	0.73 (0.53-1.04, p=0.0744)	-	-
Malignancy	No	6730 (17.3)	32274 (82.7)	-	-	-	-
	Yes	873 (24.1)	2742 (75.9)	0.65 (0.60-0.71, p<0.0001)	0.89 (0.77-1.03, p=0.1042)	0.87 (0.76-1.00, p=0.0465)	0.90 (0.78-1.04, p=0.1483)
Rheumatologic disease	No	6465 (17.2)	31062 (82.8)	-	-	-	-
	Yes	1091 (22.0)	3869 (78.0)	0.74 (0.69-0.79, p<0.0001)	0.85 (0.75-0.97, p=0.0145)	-	-
Pre-admission immunosuppressants	No	6760 (17.9)	31073 (82.1)	-	-	-	-
	Yes	780 (15.4)	4299 (84.6)	1.20 (1.11-1.30, p<0.0001)	1.28 (1.11-1.48, p=0.0006)	1.28 (1.12-1.47, p=0.0005)	1.28 (1.11-1.48, p=0.0008)
AIDS/HIV	No	7398 (17.8)	34156 (82.2)	-	-	-	-
	Yes	24 (17.0)	117 (83.0)	1.06 (0.69-1.68, p=0.8085)	0.56 (0.28-1.21, p=0.1204)	-	-
Week admission	Mean (SD)	48.1 (8.3)	50.2 (7.2)	1.04 (1.03-1.04, p<0.0001)	1.04 (1.03-1.04, p<0.0001)	1.04 (1.03-1.04, p<0.0001)	1.04 (1.03-1.05, p<0.0001)
OR multivariable							

Number in dataframe = 46376, Number in model = 16177, Missing = 30199, AIC = 13322.5, C-statistic = 0.711 (95% CI 0.700-0.721), H&L = Chi-sq(8) 4.33 (p=0.826)
OR multivariable reduced
Number in dataframe = 46376, Number in model = 17079, Missing = 29297, AIC = 13993.9, C-statistic = 0.709, H&L = Chi-sq(8) 11.00 (p=0.202)
OR multilevel
Number in model = 16902, Number of groups = 184, AIC = 13345.8, C-statistic = 0.767 (0.758-0.776)

Figure S2. Multivariable multilevel regression model of any corticosteroid administration among adult patients receiving supplementary oxygen with moderate or severe COVID-19 and admitted between the 17th of June 2020 and 14th of April, including clinical frailty (n=16902). The vertical line corresponds to OR = 1. Reference levels are omitted from the graph. All variables included in the univariable and multivariable multilevel model are shown in Table S12.

Any corticosteroid: OR (95% CI, p−value)

Illness severity	Severe COVID−19	1.51 (1.38−1.66, p<0.0001)
Age	<50	−
	50−59	1.43 (1.18−1.74, p=0.0003)
	60−69	1.40 (1.16−1.70, p=0.0004)
	70−79	1.06 (0.88−1.28, p=0.5099)
	80+	0.80 (0.66−0.97, p=0.0215)
Sex	Male	1.25 (1.14−1.37, p<0.0001)
Ethnicity	White	−
	South Asian	1.18 (0.96−1.45, p=0.1168)
	East Asian	0.84 (0.48−1.47, p=0.5382)
	Black	1.08 (0.75−1.55, p=0.6778)
	Other ethnic minority	1.14 (0.92−1.42, p=0.2188)
IMD quintile	1 (least deprived)	−
	2	0.95 (0.81−1.12, p=0.5604)
	3	0.91 (0.77−1.07, p=0.2575)
	4	1.02 (0.86−1.20, p=0.8382)
	5 (most deprived)	0.94 (0.80−1.11, p=0.5008)
Clinical frailty	1−2	−
	3−4	0.88 (0.76−1.02, p=0.0884)
	5−6	0.53 (0.45−0.63, p<0.0001)
	7−9	0.41 (0.34−0.49, p<0.0001)
Chronic cardiac disease	Yes	0.88 (0.79−0.97, p=0.0120)
Chronic pulmonary disease	Yes	0.82 (0.73−0.91, p=0.0004)
Obesity	Yes	1.35 (1.19−1.54, p<0.0001)
Chronic neurological disease	Yes	0.84 (0.73−0.96, p=0.0105)
Dementia	Yes	0.82 (0.72−0.95, p=0.0059)
Moderate or severe liver disease	Yes	0.51 (0.37−0.70, p<0.0001)
Malignancy	Yes	0.90 (0.78−1.04, p=0.1483)
Pre−admission immunosuppressants	Yes	1.28 (1.11−1.48, p=0.0008)
Week admission	−	1.04 (1.03−1.05, p<0.0001)

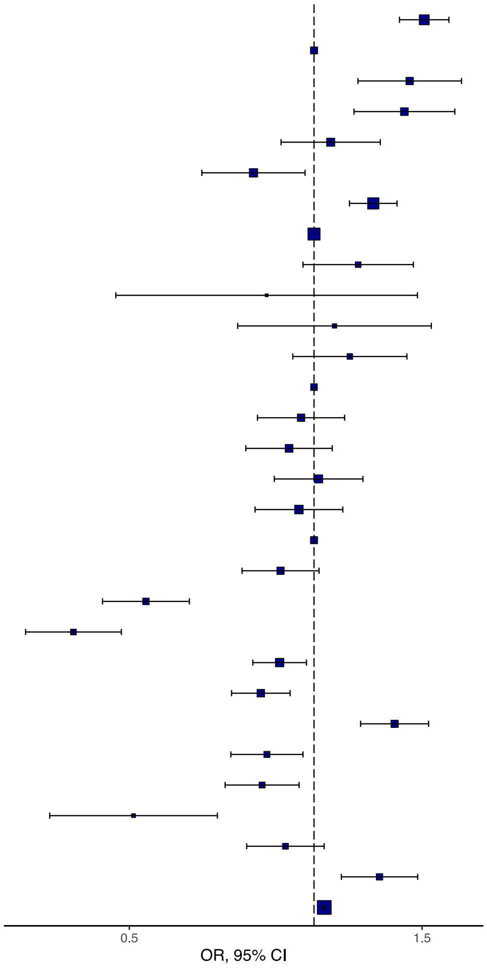

Table S13. Number of adult patients receiving any corticosteroids of all adult patients receiving supplementary oxygen at any point in their admission by week ending from the 16th of March 2020 until the 14th of April 2021.

Week ending	Any corticosteroid (n)	Total (n)	Proportion of patients receiving any corticosteroid	Percentage of patients receiving any corticosteroid
2020-03-22	328	2157	0.1520631	15.2
2020-03-29	940	6693	0.1404452	14.0
2020-04-05	1367	10662	0.1282123	12.8
2020-04-12	1194	9002	0.1326372	13.3
2020-04-19	843	5914	0.1425431	14.3
2020-04-26	533	3910	0.1363171	13.6
2020-05-03	456	2963	0.1538981	15.4
2020-05-10	302	2021	0.1494310	14.9
2020-05-17	263	1573	0.1671964	16.7
2020-05-24	186	1200	0.1550000	15.5
2020-05-31	175	877	0.1995439	20.0
2020-06-07	171	758	0.2255937	22.6
2020-06-14	161	586	0.2747440	27.5
2020-06-21	225	497	0.4527163	45.3
2020-06-28	216	429	0.5034965	50.3
2020-07-05	148	283	0.5229682	52.3
2020-07-12	131	250	0.5240000	52.4
2020-07-19	91	179	0.5083799	50.8
2020-07-26	77	156	0.4935897	49.4
2020-08-02	79	156	0.5064103	50.6
2020-08-09	87	159	0.5471698	54.7
2020-08-16	101	163	0.6196319	62.0
2020-08-23	91	151	0.6026490	60.3
2020-08-30	111	178	0.6235955	62.4
2020-09-06	141	222	0.6351351	63.5
2020-09-13	308	445	0.6921348	69.2
2020-09-20	515	736	0.6997283	70.0
2020-09-27	761	1023	0.7438905	74.4
2020-10-04	968	1353	0.7154472	71.5
2020-10-11	1227	1742	0.7043628	70.4
2020-10-18	1542	2131	0.7236039	72.4
2020-10-25	1879	2604	0.7215822	72.2

2020-11-01	2156	2876	0.7496523	75.0
2020-11-08	2132	2857	0.7462373	74.6
2020-11-15	2355	3050	0.7721311	77.2
2020-11-22	2124	2865	0.7413613	74.1
2020-11-29	1609	2230	0.7215247	72.2
2020-12-06	1392	2097	0.6638054	66.4
2020-12-13	1509	2166	0.6966759	69.7
2020-12-20	1770	2439	0.7257073	72.6
2020-12-27	1937	2639	0.7339901	73.4
2021-01-03	3009	3966	0.7586989	75.9
2021-01-10	3967	4949	0.8015761	80.2
2021-01-17	3743	4586	0.8161797	81.6
2021-01-24	3450	4158	0.8297258	83.0
2021-01-31	2432	2968	0.8194070	81.9
2021-02-07	1488	1829	0.8135593	81.4
2021-02-14	1078	1336	0.8068862	80.7
2021-02-21	914	1183	0.7726120	77.3
2021-02-28	620	779	0.7958922	79.6
2021-03-07	459	573	0.8010471	80.1
2021-03-14	340	437	0.7780320	77.8
2021-03-21	233	307	0.7589577	75.9
2021-03-28	193	254	0.7598425	76.0
2021-04-04	138	177	0.7796610	78.0
2021-04-11	100	124	0.8064516	80.6
2021-04-18	57	71	0.8028169	80.3

Figure S3. Percentage of adult hospitalised patients on supplementary oxygen who received corticosteroids per week between the 16th March 2020 and the 14th April 2021 by NHS region. Each colour represents an NHS region while the size of the circle indicates the total number of patients admitted to the NHS region in that week (total_n).

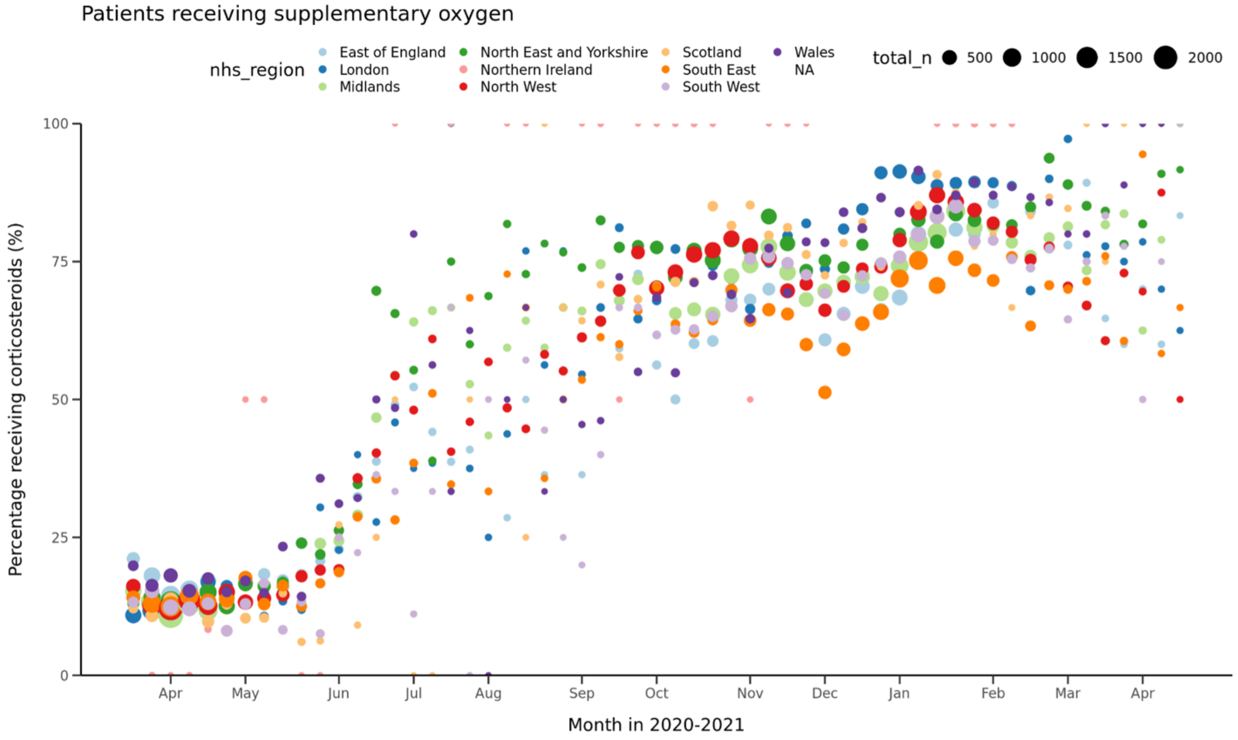

Supplement: Supplementary appendix [file mmc1.pdf]
